# Efficient real-time selective genome sequencing on resource-constrained devices

**DOI:** 10.1093/gigascience/giad046

**Published:** 2023-07-03

**Authors:** Po Jui Shih, Hassaan Saadat, Sri Parameswaran, Hasindu Gamaarachchi

**Affiliations:** School of Computer Science and Engineering, UNSW Sydney, Sydney, NSW 2052, Australia; School of Electrical Engineering and Telecommunications, UNSW Sydney, Sydney, NSW 2052, Australia; School of Electrical and Information Engineering, University of Sydney, Sydney, NSW 2006, Australia; School of Computer Science and Engineering, UNSW Sydney, Sydney, NSW 2052, Australia; Genomics Pillar, Garvan Institute of Medical Research, Sydney, NSW 2010, Australia; Centre for Population Genomics, Garvan Institute of Medical Research and Murdoch Children’s Research Institute, Sydney 2010, Australia

**Keywords:** selective sequencing, adaptive sampling, nanopore, subsequence dynamic time warping, FPGA, hardware acceleration, edge computing

## Abstract

**Background:**

Third-generation nanopore sequencers offer selective sequencing or “Read Until” that allows genomic reads to be analyzed in real time and abandoned halfway if not belonging to a genomic region of “interest.” This selective sequencing opens the door to important applications such as rapid and low-cost genetic tests. The latency in analyzing should be as low as possible for selective sequencing to be effective so that unnecessary reads can be rejected as early as possible. However, existing methods that employ a subsequence dynamic time warping (sDTW) algorithm for this problem are too computationally intensive that a massive workstation with dozens of CPU cores still struggles to keep up with the data rate of a mobile phone–sized MinION sequencer.

**Results:**

In this article, we present Hardware Accelerated Read Until (HARU), a resource-efficient hardware–software codesign-based method that exploits a low-cost and portable heterogeneous multiprocessor system-on-chip platform with on-chip field-programmable gate arrays (FPGA) to accelerate the sDTW-based Read Until algorithm. Experimental results show that HARU on a Xilinx FPGA embedded with a 4-core ARM processor is around 2.5× faster than a highly optimized multithreaded software version (around 85× faster than the existing unoptimized multithreaded software) running on a sophisticated server with a 36-core Intel Xeon processor for a SARS-CoV-2 dataset. The energy consumption of HARU is 2 orders of magnitudes lower than the same application executing on the 36-core server.

**Conclusions:**

HARU demonstrates that nanopore selective sequencing is possible on resource-constrained devices through rigorous hardware–software optimizations. The source code for the HARU sDTW module is available as open source at https://github.com/beebdev/HARU, and an example application that uses HARU is at https://github.com/beebdev/sigfish-haru.

Key Points

- Hardware-accelerated signal-matching Read Until designed for resource-constrained embedded platforms.
- A resource-efficient subsequence dynamic time warping (sDTW) accelerator for selective sequencing.
- Full proposed design (software processing layer, devices drivers, hardware sDTW accelerator): https://github.com/beebdev/HARU
- Example application using HARU and optimized C implementation of RUscripts: https://github.com/beebdev/sigfish-haru.
- Modified RUscripts (supports Python 3.6+, BLOW5 format, ONT’s R9.4 chemistry): https://github.com/beebdev/RUscripts-R9.

## Introduction

The latest third-generation nanopore sequencing technology has revolutionized the field of genomics. The portable palm-sized nanopore sequencer called the MinION produced by Oxford Nanopore Technologies (ONT) can perform direct selective sequencing, which rejects the genomic reads that are not of interest. This technique, also known as Read Until, can vastly reduce the sequencing time and cost for applications such as genetic disease identification [1, 2], cancer detection [3, 4], and the surveillance of viruses (e.g., SARS-CoV-2) and other pathogens [5, 6], as well as sequencing low-abundance species metagenomics samples [7]. However, the real-time analysis of genomic reads involves the complex and time-consuming process of aligning the read to the reference to obtain the position information. Ideally, the real-time analysis should be performed on a low-cost, low-power, and portable device [8–10], which is the aim of this article.

Existing alignment methods for selective sequencing use high-performance computing systems to meet the real-time processing requirement, compromising portability, cost-effectiveness, and power efficiency. The very first nanopore selective sequencing method tackled the alignment problem directly in signal domain [11]. It used *subsequence dynamic time warping* (sDTW) for direct signal mapping for the early R7 nanopore chemistry, which could sequence at a speed of 70 bases/s. However, with the introduction of the R9 nanopore chemistry with a 450 bases/s speed [12], sDTW-based Read Until could not keep up with a portable palm-sized MinION sequencer, even when running on a 22-core high-performance computing (HPC) system. The sDTW computation alone takes more than 98% of the total runtime.

The current base-domain Read Until implementations [13] first convert signal reads to bases using GPU-accelerated basecallers and then map them to the reference base sequence using sequence mapping techniques (e.g., Minimap2 [14]). Although the mapping techniques in the base domain are optimized and matured in the bioinformatics field, the prerequisite basecalling step is compute-intensive and is a significant bottleneck for Read Until implementations. To keep up with the sequencing rate, the execution of basecalling requires high-end GPU hardware (NVIDIA RTX 1080 for simple reference targets [13] and NVIDIA RTX 3090 for more complex targets [1]), which makes selective sequencing expensive, power-hungry, nonportable, and nonscalable. Therefore, researchers have shown significant interest in developing methods to process the raw signals directly (to avoid this compute-intensive basecalling step), and it has become an active and growing research area [11, 15–21].

**Figure 1:**
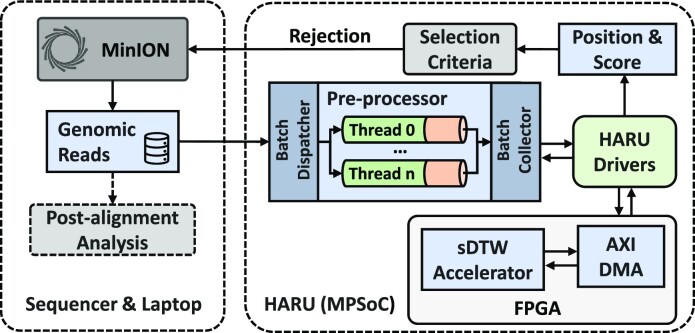
HARU overview.

In this article, to address the lack of portability and costly execution nature of existing solutions, we aim to develop a portable, low-cost, and power-efficient solution for selective sequencing in raw signal domain. We present Hardware Accelerated Read Until (HARU) (Fig. 1), a software–hardware codesign system for raw signal-alignment Read Until that uses the memory-efficient sDTW hardware accelerator for high-throughput signal mapping.

HARU primarily targets low-cost, resource-constrained heterogeneous multiprocessor system-on-chip (MPSoC) devices with on-chip reconfigurable hardware and performs efficient multithreaded batch-processing for signal preparation in conjunction with the sDTW accelerator. HARU tackles the computational bottleneck by accelerating the sDTW algorithm with field-programmable gate arrays (FPGAs). The memory-efficient sDTW accelerator for Read Until is designed by exploiting the fine-grained parallelism offered by the FPGA and has a computational time complexity of *O*(*M* + *N*). The sDTW accelerator is loaded onto the on-chip FPGA and interfaced with the software application through software drivers. Sequenced raw-signal samples are preprocessed in software before streaming into the sDTW accelerator (Fig. 1). Mapping results of the signal are then returned to the application through the software driver for postprocessing.

We demonstrate that HARU gains around 85× speedup against the original software implementation mapping the SARS-CoV-2 sequenced data on a 36-core HPC system. Furthermore, we show that HARU runs around 2.5× faster than an optimized multithreaded software implementation on the same 36-core server and around 6.5× faster than the same software running on a 10-core Intel Core i9-10850K desktop. The energy consumption of HARU is 341.7× lower than the same application executing on the 36-core server.

HARU is a complete system for selective sequencing that works on off-the-shelf devices, as opposed to being a conceptual work limited to simulation. For instance, one may purchase the targeted device used in this article (Xilinx’s Kria AI Starter Kit, which has a quad-core ARM Cortex A53 with 4 GB of RAM and an on-chip FPGA), flash the device, and execute HARU. In its current form, HARU is limited to kilobase-sized genomes. However, this is the first time a selective sequencing work is shown to be able to execute selective sequencing on such a low-power and lightweight device and, more importantly, running on off-the-shelf low-cost hardware. HARU demonstrates that selective sequencing can be performed efficiently on an edge device with an excellent price to performance-per-watt ratio. We believe this work will inspire the possibility of performing selective sequencing directly on a chip within a nanopore sequencer.

HARU can also be used as a framework for other future work intending to explore acceleration for selective sequencing on FPGAs by replacing the sDTW core in HARU. As a stepping stone for such projects, this allows quick verification of the experimental core producing practical results instead of being limited to using software simulation. We have provided step-by-step instructions and documentation on building the overarching system from scratch. In addition, the interface to the accelerator is exposed as a library so that the application layer source code can call the interface and treat the accelerator as a black box. We selected Xilinx’s Kria AI Starter Kit as the target reference device for HARU, with the intention of HARU being used as a framework for future developers focusing on similar genomics FPGA acceleration work. The *xmutil* tool on the Kria platform allows easy access to system performance and information metrics as well as fast loading and replacing of FPGA bitstreams, allowing users to quickly change hardware accelerators for different applications without rebooting the system. Xilinx’s Kria supports tools such as Vitis HLS (C to HDL generation) and PYNQ (Python framework for Zynq MPSoCs), which allows researchers with limited hardware backgrounds to design accelerators for their applications.

## Background

### Nanopore selective sequencing

Nanopore sequencers from ONT are third-generation genomic sequencers that are capable of producing long reads (currently ranging between 1 kilobases to >2 megabases) [22, 23] and are commercially available at an affordable price compared to sequencers of other techniques and generations [24]. These ONT nanopore sequencers provide genomic reads through *flow cells*, which contain a proprietary sensor array over nanopore channels embedded in a synthetic membrane [25]. During the sequencing process, the nanopore channels capture the electric current change caused by the genome molecules’ ionic current when it passes through [25]. This current signal trace is streamed to the sequencer software in real time and can later be basecalled into the corresponding nucleobase representation for later analysis [26].

A feature of ONT nanopore sequencers is the direct selective sequencing capability. These sequencers provide real-time data output streams and allow the rejection of reads at individual nanopore channels [11, 13]. This means the sequenced data can be analyzed during the sequencing and rejected before completion if decided it is not of interest. This selective sequencing process in the nanopore sequencing workflow is known as *Read Until*. ONT provides the Read Until Application Programming Interface (API) interface for software applications to access and reject the sequenced reads in real time. A rejection made through the API call will eventually be passed back to the sequencer. The voltage at the indicated channel will be reversed to eject the genomic molecule out of the nanopore [11].

For the Read Until execution to be effective, the round-trip task latency for read acquisition, analysis, and rejection signal forwarding should be completed before the majority of the subject read is sequenced by the nanopore sequencer [11]. Rejections made after most of the strand is sequenced bring no benefit as no sequencing time is saved. Existing Read Until methods perform analysis by aligning the genomic reads to the target reference and making the rejection decision based on the position and distance score. This alignment can be done using either signal or base alignment [11, 13, 15, 16, 27, 28].

#### Signal-alignment Read Until

**Figure 2:**
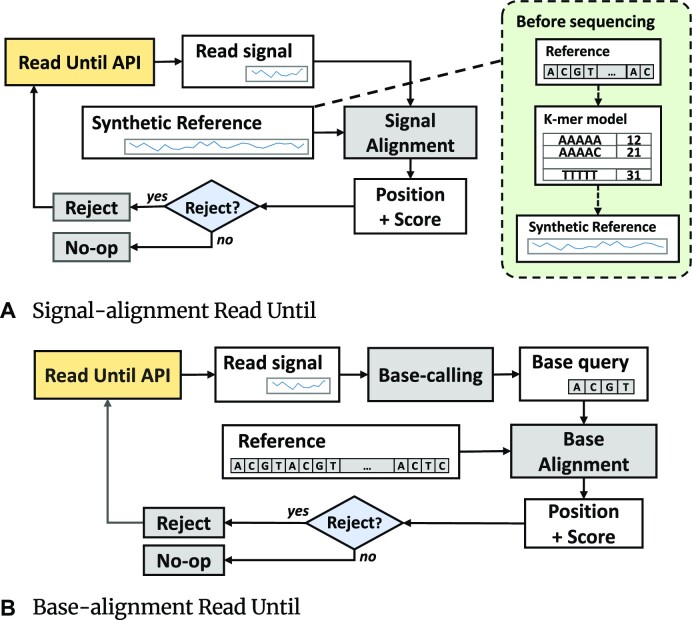
Overview of Read Until workflows.

Signal-alignment Read Until aligns raw signal reads with the reference to obtain the alignment position and distance score, as seen in Fig. 2A. Reference sequences usually are obtained in base representation (in the base equivalent “*ACGT*” characters) and need to be converted to a synthetic signal representation before being used to map the reads. This can be done using the *k*-mer model, which slides a window size of *k* bases over the base reference while the bases in the window are mapped to a value using the *k*-mer model hash-map (see Fig. 2A). The obtained alignment position and score are then used to determine if a rejection should be made, which is custom to application usage. This signal-alignment workflow was first shown by Loose et al. [11] in the RUscripts work, which is also the first Read Until implementation introduced. RUscripts is a Python implementation that uses the sDTW algorithm to align initial segments of the raw signals to the synthetic reference and can match 1 read every 0.3 seconds on a single CPU core [11]. At the time of the proposal, RUscripts could keep up with the 70-bases/s nanopore sequencing rate on a 22-core server [11]. However, as sequencing speed improved over the years, the current 450-bases/s sequencing rate [12] surpassed RUscripts’s capability of performing Read Until during sequencing. We observed that 98% of processing time is spent processing the *O*(*MN*) sDTW algorithm.

#### Base-alignment Read Until

As signal-aligning Read Until could not keep up with improved sequencing rates due to sDTW, researchers turned the focus of Read Until workflows toward base-domain techniques [13, 27]. These techniques align the genomic reads in the base domain as opposed to the signal domain, which requires an extra step of basecalling the signal to base sequences in real time before alignment (see Fig. 2B). Thanks to well-optimized multistate alignment implementations such as Minimap2 [14] and the proprietary GPU-accelerated basecaller Guppy from ONT, it can outspeed the sequencing rate to save time. Recent FPGA acceleration work on Minimap2 [29, 30] could further speed up the base-level alignment. Yet, the extensive power usage and the need for high-performance GPUs and CPUs for basecalling make base-alignment Read Until expensive and nonportable [1].

#### Potential for signal-alignment Read Until

Alignment in the signal domain and alignment in the base domain share high similarities in their algorithms and mainly differ in the sequence representation [31]. Though base-alignment methods are fast and can keep up with current sequencing rates [1, 13], basecalling is a bottleneck in current base-alignment Read Until methods. Thus, we hypothesize that signal-domain Read Until could reach better performance if enough optimization and acceleration work is applied to signal alignment as it does not require the additional basecalling step. In this work, we revitalize the direct signal approach by optimizing and exploiting hardware acceleration for the sDTW alignment methodology targeting low-cost embedded heterogeneous platforms, which also addresses the high cost of Read Until executions.

### Subsequence dynamic time warping

**Figure 3:**
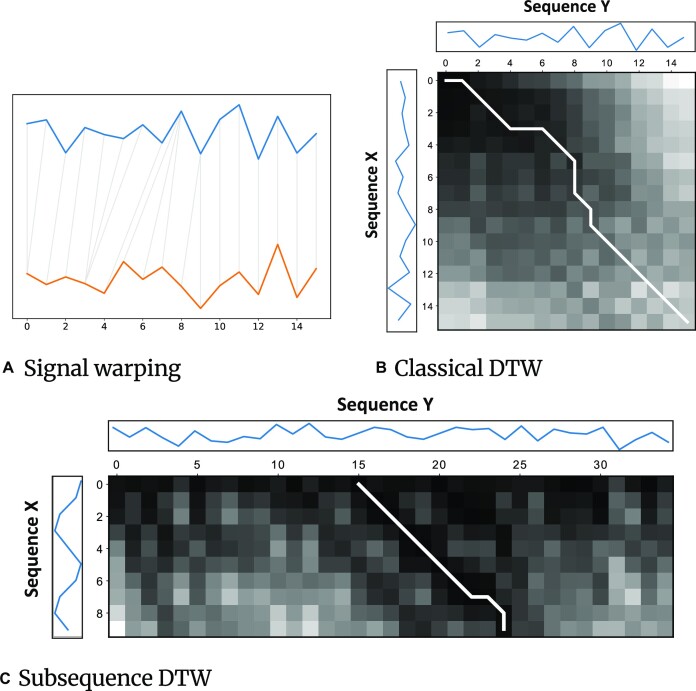
Illustration of DTW.

The dynamic time warping algorithm family includes dynamic programming algorithms that provide optimal alignment and distance metrics between 2 given time series [32] and have been widely used in pattern recognition applications in different fields [33, 34]. This optimal alignment is achieved by warping the time-series samples (see Fig. 3A), which is done by keeping an *M* × *N* sized cost matrix. The classical DTW (cDTW) algorithm performs global alignment of the signals (see Fig. 3B) [32], while the sDTW algorithm performs local alignment of the smaller sequence in the larger sequence (see Fig. 3C) [35]. Read Until attempts to find the local alignment of the query on the reference and thus uses sDTW, which is elaborated as follows.

#### sDTW problem

Given 2 sequences *X* of size *M* and *Y* of size *N* where $1\le M\le N \in \mathbb {N}$, the sDTW distance is the summation of the distance in the optimal warp path *w_optimal_*. The warp paths considered are all the paths that align the sequence *X* with any subsequence of the sequence *Y*. The dynamic programming formulation of sDTW is based on the recurrence relation of the following equation:


(1) \begin{eqnarray*}
\gamma (i, j) = \delta (i, j) + min \left\lbrace \begin{array}{@{}l@{\quad }l@{}}\gamma (i-1, j) \\ \gamma (i-1, j-1) \\ \gamma (i, j-1) \\ \end{array}\right. \end{eqnarray*}

where δ is the distance measure between samples and 1 ≥ *i* > *M*, 1 ≥ *j* > *N*. Distance measure metrics in DTW are not limited to a single method. Popular distance metrics include Euclidean distance, squared Euclidean distance, and Manhattan distance. The boundary conditions for γ(*i, j*) include γ(*i*, 0) = ∞ and γ(0, *j*) = 0, and with a bottom-up memoization, the γ values are stored in a cost matrix *C* of size *M* × *N* (i.e., *C*[*i, j*] ≔ γ(*i, j*)). γ essentially chooses, at each step, the lowest cost move. In Equation 1, γ(*i* − 1, *j*) indicates an *insertion* from sequence *X* into sequence *Y*, whereas γ(*i* − 1, *j* − 1) indicates a *match* and γ(*i, j* − 1) indicates a *deletion*. Once the cost matrix *C* is populated, the cell with the minimum distance value in the last row would be the ending position of the local alignment. Backtracking from the end position by, again, choosing the step with the lowest cost among the same dependency will give the optimal warp path and starting position (see Fig. 3C).

#### Time and space complexity

The sDTW approach is given in Algorithm 1. As shown, sDTW is *O*(*MN*) in time and space complexity due to the 2-dimensional search space. This has led to heavy computational bottlenecks in applications such as RUscripts discussed in the “Nanopore selective sequencing” section. To date, not many sDTW optimization methods exist, and cDTW optimizations such as lower bounding [36, 37] and applying global constraints [38, 39] do not bring many benefits as the necessary search space is much larger than just the diagonal connecting start and end positions of the sequences.

**Figure:**
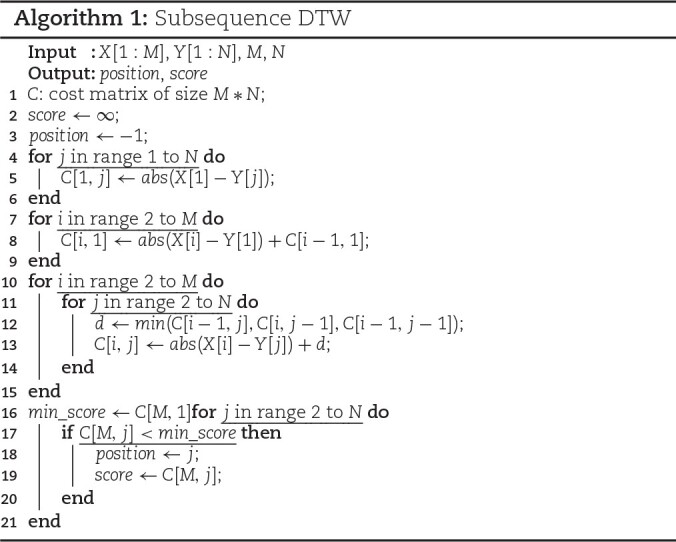


## Results

### Overall system performance

**Figure 4:**
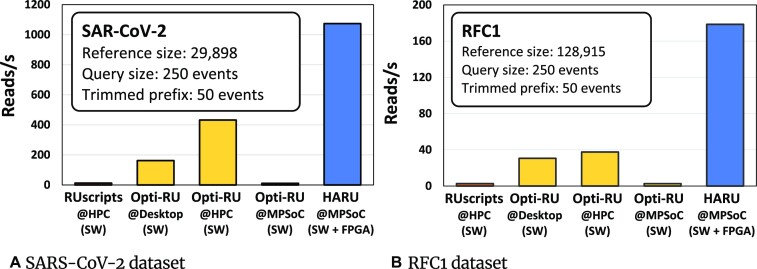
Mapping throughput for the selective sequencing.

Fig. 4A compares the overall performance of HARU for mapping all the 1.382 million reads of the SARS-CoV-2 dataset (see “Datasets” section) with software-only implementations. The y-axis of Fig. 4A is the signal mapping throughput (mapping throughput is the execution time divided by the number of reads in the dataset). The first bar in Fig. 4A represents the original Python-based RUscripts (see “Pure software implementations” section) running on the HPC with all 36 cores (throughput: 12.52 reads/s). The last bar represents our HARU system with a throughput of 1,073.83 reads/s. Thus, our HARU system is ∼85.8× faster than the original RUscripts. The second bar shows the optimized C implementation of RUscripts (see “Pure software implementations” section) on the desktop system with a 10-core i9 processor, and the throughput is 162.29 reads/s (HARU is 6.6× faster). Then, the third bar is for the optimized C implementation run with all 36 Xeon cores on the HPC, and the throughput is 432.06 reads/second. The HARU system being implemented on a low-cost embedded FPGA system is still ∼2.49× faster than the server. The fourth bar in Fig. 4A is for the optimized C implementation on the MPSoC run only on the 4-core ARM CPU, which has a throughput of 11.09 reads/s. Thus, HARU that uses the FPGA is 96.8× faster than running on the ARM processor alone.

Similarly, Fig. 4B compares the overall HARU performance for mapping all the 500,000 reads of the human dataset to the reference containing the RFC1 gene (see “Datasets” section). HARU (last bar) is 64.5× faster than RUscripts on the 36-core HPC (first bar); 5.8× and 4.7× faster than optimized C implementation on the 10-core desktop (second bar) and 36-core HPC (third bar), respectively; and 66.2× faster than the optimized C implementation on a 4-core ARM processor (fourth bar) alone.

Note that time measurement for the above throughput calculation for HARU includes all the overheads, including reading signal data from the disk, raw signal preprocessing on software, and data transfer time to/from FPGA for HARU, with our FPGA implementation running at 100 MHz. The speedups observed for HARU over other systems in Fig. 4A (SARS-CoV-2 reference) are higher compared to those in Fig. 4B (RFC1 reference) because the RFC reads are larger (128 Kb) than the SARS-CoV-2 reads (29 Kb) as explained below.

### Performance of the sDTW over reference length

**Figure 5:**
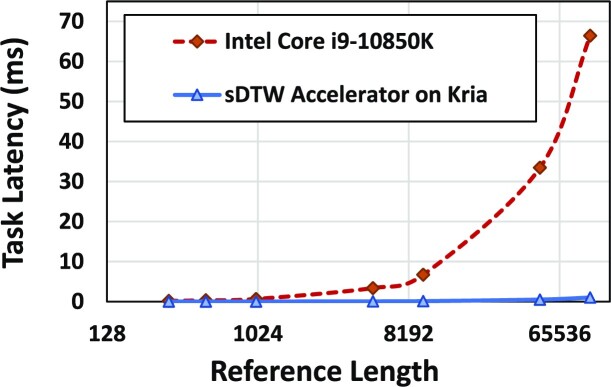
sDTW task latency.

Fig. 5 shows how the performance of our sDTW core in HARU executed on the FPGA (including the overhead for data transfer to/from FPGA) and the pure software version of DTW executed on the CPU varies over the reference length. The x-axis is the reference length on a number of bases on the log scale. The y-axis is the time taken for a single sDTW query. For the CPU (red curve), this y-axis represents the time for executing the sDTW function on a single CPU thread, whereas for the FPGA (blue curve), this is the time for processing on the FPGA plus the data transfer to and from the FPGA. Observe in Fig. 5 how the gap between the 2 curves increases with the reference length, which causes the speedup of HARU over the CPU to increase with increased reference size. This behavior is due to a band of cells being computed in parallel on hardware using a processing elements (PE) chain (see “Resource-efficient sDTW accelerator” section).

### The time breakdown for different processing steps

**Figure 6:**
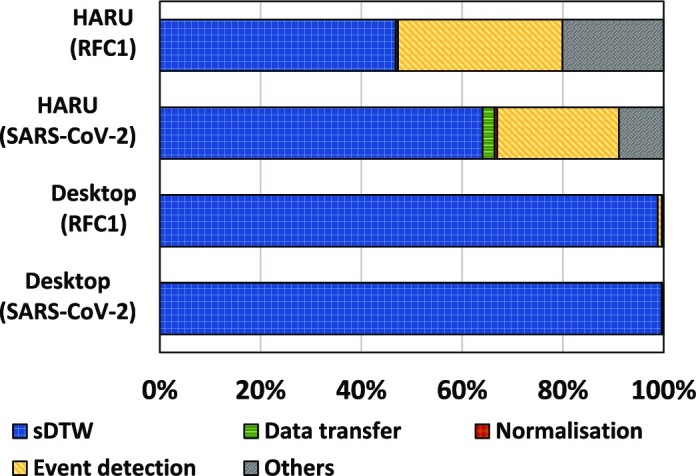
Process time breakdown.

Fig. 6 compares the percentage of time spent on different processing steps for HARU versus the optimized software implementation in percentages. Due to the significant speedup of sDTW, the percentage of runtime spent on sDTW is <64% for the SARS-CoV-2 dataset and >46% for the RFC1 data set (top 2 bars), whereas this was >98% for software (bottom 2 bars). Note that “others” in Fig. 6 is the time spent loading data from the disk, reference preparation, and writing the output.

### Accuracy

**Figure 7:**
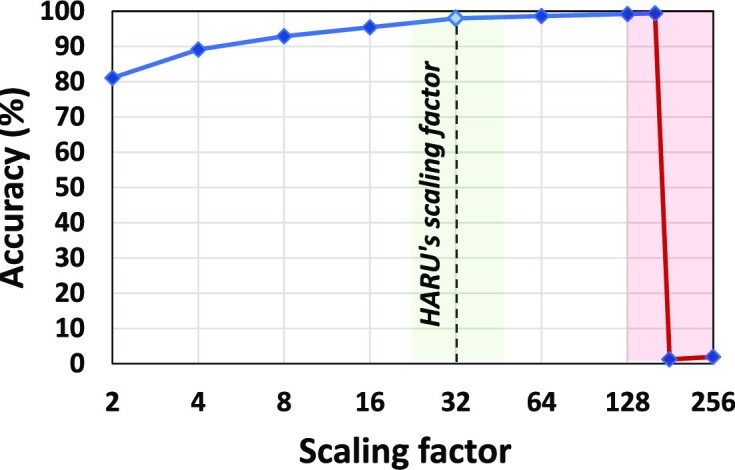
Accuracy against scaling factor.

Fig. 7 shows the accuracy of the accelerator using different scaling factors (discussed in the “Software processing layer” section). Accuracy in Fig. 7 is calculated as a percentage of the number of mapping positions similar to results produced from sDTW computed on software using 32-bit floating points. Observe that a scaling factor of 2 yields a limited accuracy (80%), while increasing the scaling factor gradually converges the accuracy toward 100%. However, when scaled above 128, the distance cost accumulation results in data overflow during sDTW, which largely impacts the alignment accuracy. In HARU, we have used a scaling factor of 32 to prevent overflow while having an accuracy close to 99%. Refer to Supplementary Note 1 for further information on using a fixed-point and a static scaling factor.

### Energy comparison

**Figure 8:**
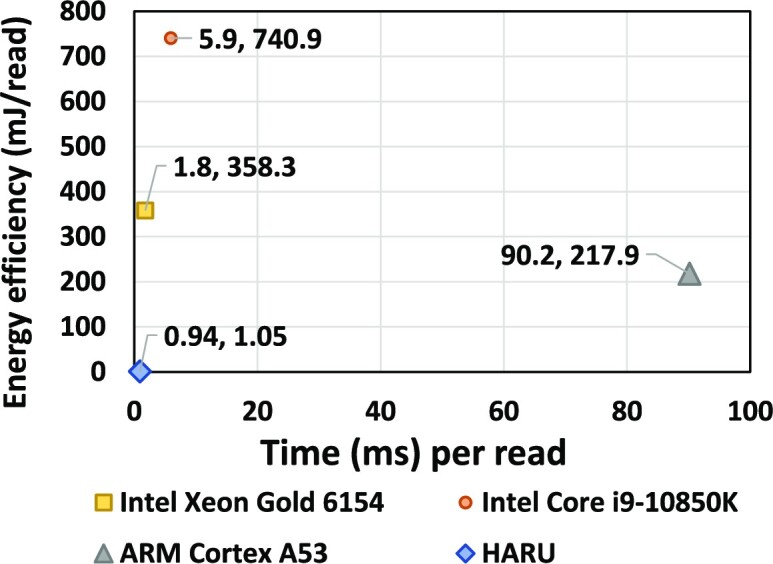
Energy and performance.

Fig. 8 shows the estimated energy efficiency (y-axis) plotted against the execution time (x-axis) for HARU and optimized software-only implementations on different processors. HARU’s overall performance and energy efficiency are considerably lower (close to the origin of the graph: time 0.94 ms/read and energy 1.05 mJ/read) than the optimized version running on ARM (90.2 ms/read, 217.9 mJ/read), Intel Core-i9 (5.9 ms/read, 740.9 mJ/read), and Intel Xeon Gold processor (1.8 ms/read, 358.3 mJ/read). The energy-delay product is 644.94 for the server but 0.987 for HARU. Thus, HARU is 650× better in terms of energy-delay product. The energy consumed for HARU and the ARM processor was estimated using the power estimates reported by Vivado in the synthesis report. In contrast, the thermal design power (TDP) value reported in the processor specification was used for Intel processors. For additional power analysis information for the HARU system on the Kria device, please refer to Supplementary Note 8.

### Resource utilization

The resource utilization for our sDTW accelerator, which loads the reference signal to the on-chip block RAM memory before running, with a single query processor on the Kria board, as reported by the Vivado synthesis report, is shown in Table 1.

**Table 1:** sDTW accelerator resource utilization.

Resource	Available	Used (utilization)
CLB LUT	117,120	21,121 (18.03%)
CLB Registers	234,240	16,798 (7.17%)
CARRY8	14,640	1,787 (12.21%)
F7 Muxes	58,560	9 (0.02%)

Note that we used a single query processor for all the above experiments to show the bare minimum performance on a low-end FPGA platform. As shown in Table 1, the maximum utilization (CLB LUT) is <20%; thus, in theory, the Kria board can fit up to at least 4 parallel query processors with some engineering effort. In fact, we have an experimental branch that does not use on-chip block RAM to store references beforehand and directly streams reference signals together with queries. This means multiple accelerators on the same FPGA will not have critical paths in between accelerators. For the postimplementation resource utilization of 4 accelerators targeting the Xilinx Kria AI Starter Kit, see Supplementary Note 2.

### Comparison with alternate methods

The analysis in the preceding subsections represents the most equitable comparisons possible. In this subsection, we attempt to compare HARU with other existing alternate methods. We must acknowledge that making a direct comparison is challenging as different methods are tailored toward different goals and intended for specific systems. Also, it is important to note that each method possesses its own distinct advantage and could be used complementarily.

#### Comparisons with DeepSelectNet and Guppy+Minimap2

**Figure 9:**
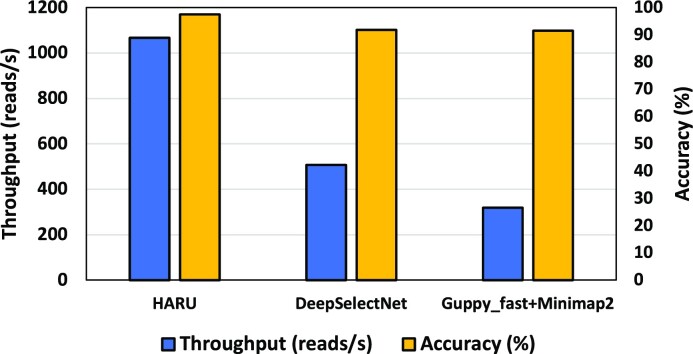
Comparison between HARU and state-of-the-art methods.

To compare HARU with DeepSelectNet [19] (an enhanced neural network–based method based on SquiggleNet [20] to classify reads from 2 classes of species) and the approach used in Readfish [13] (Guppy fast basecalling followed by Minimap2 for mapping), we used a dataset containing reads from 2 species, SARS-CoV-2 and yeast (see Methods, Supplementary Notes 3 and 4). DeepSelectNet was executed on a server with a Tesla V100 GPU (as the proof-of-concept implementation is not supported on an edge GPU). Without a GPU, neural network–based methods will be impractically slow. HARU executing on the Xilinx Kria embedded platform (1,066.3 reads/s) was yet 2.103× faster (Fig. 9) than DeepSelectNet running on the server (507.1 reads/s). As Guppy binaries for ARM processors are available and Minimap2 can be easily compiled for ARM [40], we executed Guppy_fast+Minimap2 on an NVIDIA Jetson Xavier edge GPU device as Guppy is impractically slow without a GPU (see Methods). HARU was still 3.354× faster than Guppy_fast+Minimap2 (317.94 reads/s). In the Guppy_fast+Minimap2 approach, Guppy took 96.4% of the time, demonstrating that in base alignment–based selective sequencing methods, basecalling is the bottleneck. The accuracy of HARU (97.41%, Methods, Supplementary Note 4) was better than DeepSelectNet (91.78%) and Guppy_fast+Minimap2 (91.46%).

Note that Python-based DeepSelectNet is a proof-of-concept design to run on servers and is not optimized for performance. Therefore, the aforementioned numerical values should not be interpreted as definitive, as the method could potentially be optimized for embedded systems. When comparing with Guppy+Minimap2, note that Guppy was executed on a GPU, while HARU is designed for an FPGA architecture. It is possible that implementing Guppy on an FPGA could improve its performance. The accuracy of Guppy+Minimap2 was evaluated using default parameters in Minimap2, and parameter tuning may result in better accuracy. However, such work is beyond the scope of this current study.

#### Comparison with UNCALLED

To compare HARU with UNCALLED, we mapped SARS-CoV-2 reads to the SARS-CoV-2 reference and compared the mapping location of reads reported by UNCALLED and HARU to Minimap2’s mapping (see Methods and Supplementary Note 4). UNCALLED was executed on a Rock64 edge-computing board, which has a quad-core ARM Cortex A53 processor with 4 GB of RAM, similar to the Kria device used for HARU. UNCALLED has many software dependencies and requires a package manager, which is not available on the Kria device running PetaLinux (see Methods). HARU’s throughput (1,066.33 reads/s) is 36.85× higher than UNCALLED on Rock64 (28.94 reads/s). The accuracy of UNCALLED (91.2%) is still lower than HARU’s (97.41%).

Note that when comparing UNCALLED with HARU, UNCALLED is executed on the CPU while HARU runs on the CPU and FPGA heterogeneously. The results above must not be wrongly interpreted that UNCALLED is not lightweight, in fact, UNCALLED is much less CPU demanding than sDTW and scales well for larger references. While it is not in the scope of this work, optimizing UNCALLED and implementing it on FPGA could yield better results.

#### Comparison with SquiggleFilter

SquiggleFilter [18] is a conceptual ASIC design for selective sequencing. As it is a conceptual ASIC design work yet to be fabricated and integrated with the envisioned system on chip (SoC) [18], we are unable to compare the performance throughput and accuracy. However, with the provided HDL source code, the resource utilization of HARU and SquiggleFilter can be compared. We set the SquiggleFilter design to use 2,000 PEs as claimed in [18], set the target device to the Kria AI starter kit, and manually synthesized the individual modules (as the design does not include a synthesizable top-level module orchestrating all submodules). Postsynthesis results show that the PE used in SquiggleFilter requires 2.15× more CLB LUTs (88), 5.81× more CLB Registers (93), and 2.75× more CARRY8 resources than HARU’s PE in the sDTW accelerator (41, 16, and 4, respectively). As SquiggleFilter requires 2,000 PEs for a single tile of accelerator (while HARU requires only 250 as it uses events), the warper in SquiggleFilter requires 8.44× more CLB LUTs (178,553), 11.54× more CLB Registers (191,991), and 12.5× more CARRY8s (22,002) than the total resource utilization of HARU’s sDTW accelerator (21,158, 16,634, and 7,160, respectively). Note that this comparison for SquiggleFilter is excluding the normalizer, mean finder, and mean absolute deviation finder, and its top-level entity. See Supplementary Note 4 for a more detailed resource comparison.

We also note that although claimed to be verified on FPGA, SquiggleFilter is primarily an ASIC design work. The results above target the Kria AI Starter Kit device that HARU uses, and synthesis results may differ based on target devices. Nevertheless, HARU shows to have an advantage over SquiggleFilter when targeting FPGAs for deployment with its much more efficient resource utilization. In addition, HARU is a complete system integrated with off-the-shelf hardware devices with software support.

## Methods

### Design of hardware accelerated Read Until

**Figure 10:**
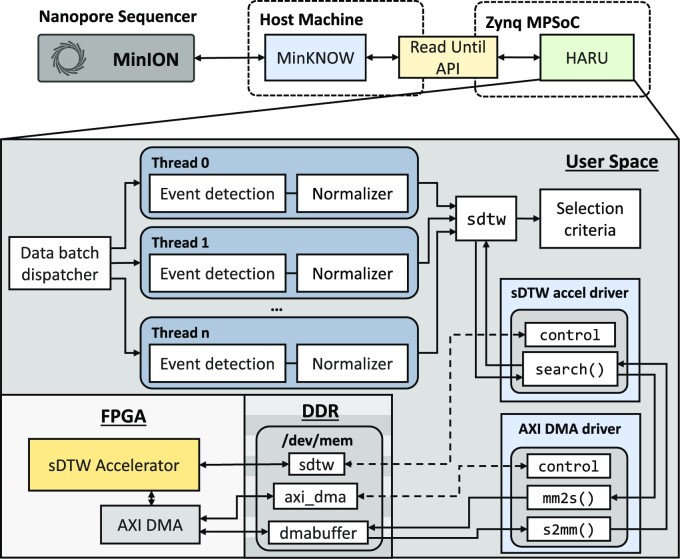
HARU architecture.

HARU targets low-cost MPSoCs with on-chip FPGA to perform selective sequencing processing. Fig. 10 shows the architecture of HARU in an ONT nanopore sequencing workflow. HARU consists of 3 main components: the software processing layer, device drivers for the accelerator and associated hardware, and the hardware sDTW accelerator. The software processing layer, discussed in the “Software processing layer” section, uses a multithreaded batch processing architecture to perform raw read signal preprocessing and is customizable based on the selection criteria. The device drivers, discussed in the “HARU device drivers” section, are designed to provide high-throughput data transferring of query and reference signals. Lastly, the resource-efficient sDTW accelerator, discussed in the “Resource-efficient sDTW accelerator” section, performs high-throughput sDTW for the selective sequencing use case.

#### Software processing layer

The software processing layer of HARU is the front end of the HARU design running on the processing system on the MPSoC. Its main tasks include preprocessing the reference sequence and raw signal reads and the final selection decision. Since references are obtained in base representation as discussed in the “Nanopore selective sequencing” section, the initialization step of the software processing layer forms the synthetic reference signal for the forward and reverse representation of the base reference (this is needed since DNA molecules are double-stranded) using the *k*-mer model for the flowcell type. Then, in preparation for the sDTW computation in hardware, the reference signal is normalized using *z*-score normalization. Since the data types used for the signal and cost matrix in the sDTW accelerator are 16-bit fixed-point types (discussed in the “Resource-efficient sDTW accelerator” section), the normalized values are scaled with a scaling factor to preserve signal resolution.

During the genome sequencing step, the software layer collects sequenced data from the nanopore sequencer in batches, which are then dispatched into multiple threads for efficient computing of preprocessing (see Fig. 10). Each thread performs event detection on the raw signal samples to reduce sample data size for the sDTW accelerator. This is done until enough events are collected. For the R9.4 flowcell, 250 events are typically adequate for mapping and would require roughly 0.4 to 0.8 seconds of data collection, which includes the time to obtain around 50 to 300 events that belong to the read adapter and then followed by the actual 250 events of the query (see Supplementary Note 5 for more information). After the collection, the events are normalized and scaled with the same scaling factor used in the reference signal preparation. When threads finish the preprocessing, the processed data are gathered and sent to the sDTW accelerator for processing using the drivers. After which, the mapping position and the similarity score are used to decide whether the read should be rejected.

#### HARU device drivers

To control and use the hardware accelerator in the software processing layer, we designed the software device drivers to have 2 main data paths (see Fig. 10). The first data path is the control path of the accelerator, which uses the AMBA AXI4-Lite protocol to configure the control registers and read status registers in the software. The accelerator’s physical address is memory-mapped to the virtual address space for user space applications to use.

The second data path is for the query and reference sequences. To prevent data transfer from becoming a bottleneck, we use the AMBA AXI4-Stream protocol to stream query and reference data into the accelerator at a high-throughput rate. This is done by using the AXI direct memory access (DMA) module to point to a physical hardware address to stream data to and from. By calling the driver function for processing the query, the sDTW accelerator driver initiates the transfer from the query and reference buffers to the transfer buffer on double data rate (DDR) memory dedicated to AXI-stream communication and the FPGA. Our benchmarks show that data can be sent to and from the accelerator at a throughput of 330 MB/s.

#### Resource-efficient sDTW accelerator

As discussed in the “Subsequence dynamic time warping” section, the standard sDTW algorithm has *O*(*MN*) time and space complexity due to the computation of the cost matrix. The computation of a cell value in the cost matrix requires comparing 3 neighbor cell values, making the exploitation of available hardware parallelism harder. Also, the preservation of the full cost matrix does not scale well if directly implemented on resource-constrained FPGA devices. We identified that the backtracking of the cost matrix to obtain the warp path is unnecessary for Read Until as the ending position is adequate to make the rejection decision. We provide the following optimizations over sDTW to obtain a resource-efficient high-throughput sDTW accelerator.

##### Cost matrix memory optimization

The need to preserve the *M* × *N* sized matrix for backtracking was discussed in the “Subsequence dynamic time warping” section. However, for selective sequencing, the obtained end position of the alignment is adequate to determine the location of the current query; thus, the backtracking step for obtaining the starting position is unnecessary. Consequently, preserving the whole cost matrix values is unnecessary, and a cost array of *M* + 1 is sufficient. Algorithm 2 shows the sDTW algorithm after the cost matrix size is reduced. The outer loop (line 4 of Algorithm 2) iterates through the whole reference sequence, while the nested inner loop (line 8 of Algorithm 2) iterates through the column at each reference sample. During each iteration of the inner loop, the computation of the recurrence equation is performed, and the computed value is stored in the cost array that is the same size as the query. Once the inner loop completes, the current minimum score and position values are updated if the last cell of the cost matrix is smaller than the current minimum score. As the computation is done in the same way as the original sDTW with the whole cost matrix, there is no impact on accuracy from this optimization.

**Figure:**
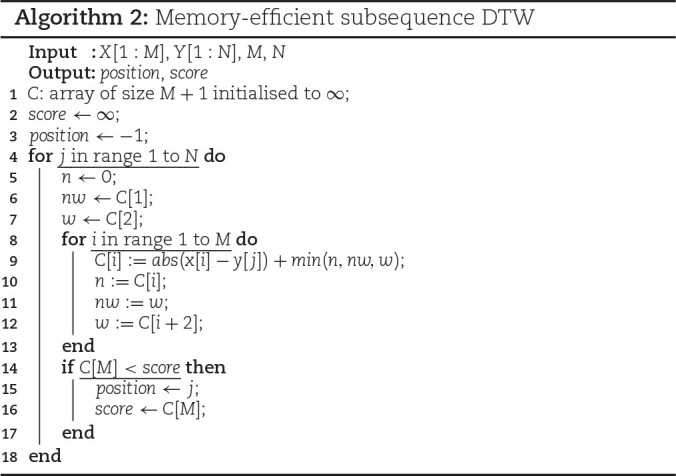


##### Operation pipelining

**Figure 11:**
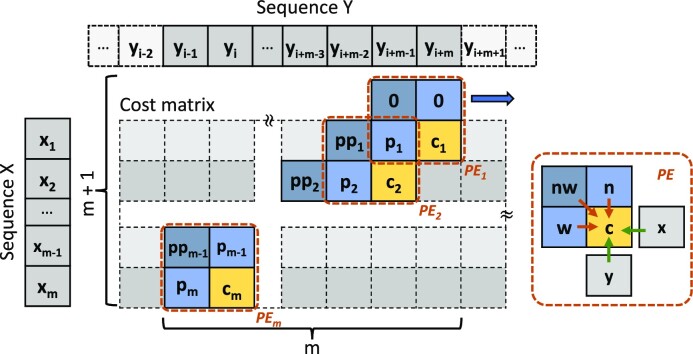
Pipelined execution of Algorithm 2.

The sDTW cost matrix size reduction explained above optimizes the space complexity of the computation for selective sequencing. However, the algorithm’s execution is still sequential and has *O*(*MN*) time complexity. Computing the whole column in parallel by unrolling the inner loop is not feasible due to the data dependency in the recurrence equation that needs waiting until the *n* value is ready (see Algorithm 2). We observe that once the first iteration of the inner loop for the column is completed, all data dependencies for the first inner loop iteration for the next column are ready. By pipelining the outer loop computation, an oblique column is formed that is computed in parallel, as shown in Fig. 11. This oblique column traverses through the reference sequences, reducing the time complexity from *O*(*MN*) to *O*(*N*) since the *N* query size is now computed in parallel. Since all cell computations are computed only after the dependencies are satisfied, pipelining does not affect the accuracy of sDTW.

##### Fixed-point data representation

After the optimization above, the hardware’s computational complexity is *O*(*M*). However, the actual time needed is (*M* + *N* − 1) × *II*, where *II* is the initiation interval (i.e., the number of cycles between loop iterations). In pipelined Algorithm 2, *II* is how fast the reference equation *C*[*i*] ≔ *abs*(*x*[*i*] − *y*[*j*]) + *min*(*n, nw, w*) can be computed. Normally, 32-bit floating-point data types are used for the sDTW computation to preserve the precision after the sequences are normalized. This is expensive to implement in hardware regarding resources and execution time. By using a fixed-point representation with fewer data bits and scaling the sequence values using a scaling factor, the recurrence equation can be computed in hardware rapidly and efficiently while keeping sufficient precision. We chose 16-bit fixed points with a scaling factor of 2^5^ as it gives sufficient precision and keeps *II* at 1 clock cycle (see section Results on accuracy). Using fixed point with a static scaling factor will decrease the accuracy slightly as we are using fewer bits to represent the decimal points compared to floating points. Nevertheless, this data representation will still provide close to zero difference in mapping accuracy compared to using floating points (see Supplementary Notes 1 and 6 for more detail).

##### HARU’s sDTW Accelerator

**Figure 12:**
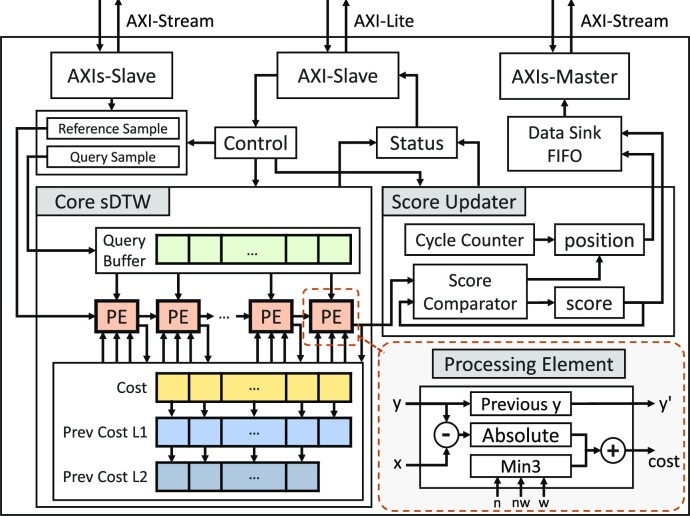
sDTW hardware accelerator design for HARU.

The oblique parallel-computed column mentioned above uses a PE-chain structure where data-dependent neighbor cells are shared among the PEs (Fig. 11). As shown in Fig. 12, the shared values are stored in 2 register arrays of size *M* (L1 being the previous cost array and L2 being the second previous cost array). At each iteration, the costs in the L1 array are shifted into the L2 array, while the current costs are passed onto the L1 array. Each PE computes the recurrence equation, which takes the Manhattan distance (δ = |*x*[*i*] − *y*[*j*]|) and adds the minimum of the 3 neighbor cells (see Equation 1). Samples of the reference sequence are first streamed into the first PE of the chain and are then passed along to successive PEs in each iteration. In the “Software processing layer” section, we discussed that the software processing layer uses multithreaded batch processing to perform event detection and normalization. The event detection decreases the query size to make the *M* term smaller in the algorithm complexity. We choose to use a size of 250 events (see Supplementary Note 5), giving the accelerator a PE chain of 250 PEs. In total, it takes *N* + 250 − 1 clock cycles to complete the full search.

### Experimental setup

The HARU system, proposed in the “Design of hardware accelerated Read Until” section, was implemented on Xilinx’s Kria AI Starter Kit with a Zynq Ultrascale+ XCK26-SFVC784-2LV-C MPSoC. This board contains a processing system with a quad-core ARM Cortex A53 CPU and 4 GB of DDR4 memory (specifications on column “MPSoC” in Table 2). Implementation details of HARU are discussed in the “HARU implementation” section. This HARU implementation is compared to 2 pure software implementations, discussed in the “Pure software implementations” section. These 2 software versions are executed on a desktop computer comprising a 10-core Intel Core-i9 processor and a high-performance computer (server) with a 36-core Intel Xeon processor (specifications are in Table 2). We performed the experiments on 2 representative datasets, detailed in the “Datasets” section.

**Table 2:** Computational platforms.

System	HPC	Desktop	MPSoC
CPU	Intel Xeon Gold 6154	Intel Core i9-10850K	Arm Cortex-A53
CPU cores	36	10	4
Clock rate	3.00 GHz	3.60 GHz	1.5 GHz
RAM	377 GB	32 GB	4 GB
FPGA	No	No	Yes

#### HARU implementation

The operating system running on the processing system of the board is a customized embedded Linux image generated using Xilinx’s Petalinux 2021.1 tool. To show the bare minimum throughput of the accelerator, our sDTW accelerator is synthesized with a single-query processor in the accelerator clocked at 100 MHz. The number of query processors that can fit in the FPGA depends on the available resource on the device; see the “Resource utilization” section for resource utilization of the accelerator with a single-query processor.

##### sDTW hardware accelerator

The sDTW accelerator was implemented using Verilog Hardware Descriptive Language (HDL). Synthesis was performed using Xilinx’s Vivado 2021.1. The control bus interface for the accelerator uses the AMBA AXI-Lite protocol. We use the AMBA AXI-Stream protocol through the AXI DMA hardware in the FPGA for high-throughput data transfer for the query and reference data.

##### HARU driver

Device drivers were implemented for the hardware accelerator and AXI DMA in the C programming language. The accelerator and AXI DMA drivers memory-map the physical address of corresponding devices into the virtual address space for utilization by the user space applications. The shared communication memory buffers between software and FPGA are preserved on the DDR memory, which is allocated during the initialization stage.

##### Software processing layer

The software processing layer that prepares the raw signals and performs the selecting decision was implemented in the C programming language. For benchmarking experiments, the software loads raw signal data in the BLOW5 format [41] from a USB 3 external hard drive connected to the Kria board. Raw signals for a batch of reads are first loaded to the Random Access Memory (RAM) and are preprocessed using multiple threads implemented using POSIX threads. Preprocessing steps include event detection, prefix trimming, and normalization (explained in the “Software processing layer” section). Then, sDTW is performed on each read in hardware by iteratively calling the HARU driver. Once the mapped positions and the DTW scores are available for the whole batch, the software computes the mapping quality (MAPQ) [42] and executes the selection criteria based on this score [11].

#### Pure software implementations

##### RUscripts

Original RUscripts, written by Loose et al. [11] using Python 2.7, has reached end-of-life support and targets ONT’s R7 Nanopore chemistry, which is no longer in use. We modified RUscripts to work on Python 3.6+ and extended it to support BLOW5 format and ONT’s current Nanopore chemistry R9.4. This support for R9.4 chemistry is implemented by integrating the R9.4 pore model and R9.4 event detection parameters [43, 44].

##### Optimized RUscripts in C

As the Python RUscripts is not efficient enough for a fair comparison, we implemented a multithreaded C implementation that follows similar algorithmic steps. This implementation in C is very similar to the software explained above (see the “Software processing layer” section), except that sDTW on the CPU is called with multiple threads instead of using the FPGA accelerator. The sDTW computation on the CPU is performed using the optimized sDTW implementation in the *mlpy* library [35].

#### Datasets

HARU was tested against combinations of software running on the systems mentioned in Table 2 on 2 datasets. The first dataset is the SARS-CoV-2 genomic reads sequenced on a MinION R9.4 flowcell and has a total of 1.382 million reads (Table 3), publicly available at [45]. The SARS-CoV-2 genome (MN908947.3), which is 29,903 bases long, is used as the reference for this experiment. The second dataset is a subset of a NA12878 human genome reference sample containing 500,000 reads sequenced on a PromethION R9.4 flowcell (Table 3), publicly available at [41]. This dataset is mapped to a reference constructed by extracting the region chr4:39262456-39391375 (128 Kb long) of the human genome (hg38). This region includes the RFC1 gene, which contains an important pathogenic variant indicative of hereditary cerebellar ataxia disease, and selective sequencing has been applied [1] for accurate diagnosis.

**Table 3:** Datasets.

Target	SARS-CoV-2	RFC1
Type	Viral genome	Partial human genome
No. of bases	29,903	128,915
Search space size	59,806	257,830
No. of reads	1,382k	500k
SLOW5 file size	5.5 GB	39 GB

#### Performance evaluation

We measure the overall execution time of mapping all reads of the provided datasets by using the *gettimeofday* function in C. This execution time is divided by the number of reads in the dataset to calculate the signal mapping throughput. Note that all our time measurements used in throughput calculation include all the overheads, including reading signal data from the disk, raw signal preprocessing on software, and data transfer time to/from FPGA for HARU.

#### Comparison with alternate methods

To compare HARU with DeepSelectNet and the approach in Readfish (Guppy2 followed by Minimap2), we used the curated test data for SARS-CoV-2 and yeast from [19] that contained 20,000 reads from each species. DeepSelectNet was installed and executed on a workstation with a Tesla V100-16GB GPU, 20 CPU cores, and 384 GB RAM (Supplementary Notes 3 and 4). Guppy 6.1.3 and Minimap2 2.20 were set up and run on an NVIDIA Jetson Xavier AGX board. Note that we activated the 15-W nvpmodel on this device to make the specification closer to what is available on the Kria board used for HARU. The *dna_r9.4.1_450bps_fast.cfg* model was used for Guppy, and a combined reference genome of SARS-CoV-2 and yeast was used for Minimap2 (Supplementary Note 7). For DeepSelectNet, the first 4,500 signal samples were used (default options), with the same number of signal samples used for Guppy+Minimap2. For HARU, we used the default prefix and query size in HARU (50 + 250) that approximately relates to around 1,500 to 3,000 signal samples. The accuracy of each method was calculated as the sum of true positives and true negatives divided by the total reads (Supplementary Note 4). For HARU, where the reads from each species were mapped against the SARS-CoV-2 reference, the cutoff value for sDTW scores to determine if a read mapped to SARS-CoV-2 or not was determined, as explained in Supplementary Note 3.

To compare against UNCALLED, we used 40,000 reads from the SARS-CoV-2 dataset (in subsection Datasets). UNCALLED was installed on a Rock64 embedded device that has a similar computing power (quad-core ARM Cortex A53 with 4 GB RAM) to the Kria board used for HARU. This is because UNCALLED has many dependencies, and enabling support for the Kria platform, which runs a custom PetaLinux distribution, is laborious. Despite the Rock64 board supporting Ubuntu and the apt package manager along with Python/PIP and C/C++ build tools, we still had to manually intervene in the UNCALLED installation scripts to enable support for HDF5 and BWA dependencies to build on ARM. Both HARU and UNCALLED were executed using the SARS-CoV-2 reference, and the accuracy was calculated by using UNCALLED pafstats by comparing mapping locations to Minimap2 mappings as the truth set (Supplementary Note 7). The *–chunk-time* and *–max-chunks 1* parameters in UNCALLED were used to limit the number of signal samples to 3,200 (Supplementary Note 7). For generating the truth set using Minimap2, the complete reads were basecalled and mapped.

## Discussion

### Signal-level versus base-level selective sequencing

The field of selective sequencing is a nascent area, and to date, no definitive solution has emerged as the panacea. Both signal-level and base-level approaches to selective sequencing have advantages and disadvantages, and determining which is the optimal approach at this stage is more of a philosophical debate.

With the methods available to date, basecalling raw signals obtained from the sequencers to convert signals to the base domain, followed by using optimized alignment tools such as Minimap2 (the approach described in Readfish), is the most practical approach if large genomes are involved because base-level aligners have matured over the past decade of research and development and are highly optimized to make base-level selective sequencing practical. However, for basecalling, regardless of the GPU acceleration effort performed by ONT over the years, basecalling is still the major bottleneck for base-domain selective sequencing, taking 96% of the execution time for Guppy fast basecalling + Minimap2. Furthermore, basecalling is not portable or scalable due to the compute power constraints, and if selective sequencing is ever to be done on an integrated chip within the sequencer, basecalling approaches would require a more costly system and possibly come at a much larger form factor.

The goal of signal-level selective sequencing is to completely bypass the basecalling step and, instead, directly map the raw signal to the reference. This is an emerging and immature field and will inevitably require a substantial period of time to achieve the same level of maturity as base-level selective sequencing. Since the concept of nanopore selective sequencing was introduced, a range of different signal-level selective sequencing methods has been explored, including RUscripts [11], cwDTW [17], UNCALLED [15], sigmap [16], and, more recently, RawHash [21], DTWax [46], and RawMap [47].

In addition, directly passing raw signals into neural networks is also being explored as opposed to using classical algorithms for mapping, including works such as SquiggleNet [20], DeepSelectNet [19], and RISER [48]. However, neural network–based approaches are currently limited to classifying reads between 2 target species, and getting mapping coordinates is not yet possible. Moreover, neural network–based methods require training the model for each dataset, which makes it less flexible and requires more preparation than the classical approaches.

The data rate of nanopore sequencers is comparable to modern camera sensors on mobile devices today. Considering the amount of raw signal processing being performed for sensors on mobile devices, it is promising to envision signal-level nanopore selective sequencing done efficiently within nanopore sequencers, if this level of miniaturization is ever reached for selective sequencing compute requirements. In summary, signal-level selective sequencing is an exciting area worth investigating together with base-level selective sequencing.

### Limitations and future work

In our proof-of-concept implementation of HARU, the reference sequence is first loaded onto the FPGA’s on-chip memory (block RAM) at the beginning of the execution. During alignment, the PE chain streams the reference samples from the block RAM to the first PE (Fig. 12). On-chip memory (block RAM) on the Xilinx Kria board used for evaluation is limited to 5.1 MB, thus limiting the maximum reference sequence size to 295 kilo-bases. To eliminate this limitation, future work could directly stream the reference together with the query sequence before each sDTW call (there is currently an experimental branch for this; see Supplementary Note 2). However, even with HARU (linear time complexity for sDTW), performing sDTW of a query on a gigabase-sized genome like the human genome is impractical (estimated to take 3 seconds for a query). Nevertheless, when processing gigabased-sized genomes, HARU is intended to be used in the final refinement step when potential mapping locations (a few reference sequence segments that are small in size) are first found using a heuristic method. Such a heuristic method that can currently map nanopore signals directly to gigabased-sized genomes does not exist. However, methods such as Sigmap [16], UNCALLED [15], and RawHash [21] are already setting the foundation for scalable direct signal mapping.

Future work can also improve the throughput by implementing multiple parallel sDTW cores for coarse-grain parallelism. Our sDTW processor uses less than 20% of the LUT resources of the FPGA, as mentioned in the “Resource utilization” section. Thus, resources are sufficient to fit multiple parallel processors, increasing the theoretical throughput. A high-end FPGA board with a larger area could support even more processors; for instance, Xilinx’s Versal VP2802 FPGA has sufficient resources to theoretically fit 140 parallel processors (see Supplementary Note 2 for experimental explorations of deploying 4 accelerators in HARU). However, such work also would require eliminating other bottlenecks that would arise. For instance, the 30% of execution time currently spent on the signal preprocessing (Fig. 6) would then become a bottleneck and require acceleration.

Our implementation of HARU loads raw signal from the BLOW5 file format because the slow5lib library is lightweight (with minimal dependencies), thus easily allowing the cross-compilation to target the Kria platform. Running MinKNOW on the Kria platform is theoretically possible but is far from practical due to being closed source. Even if MinKNOW were open source, potential issues with hundreds of bulky dependencies would make cross-compilation impractical. Potential workarounds could include a server–client approach where MinKNOW runs on a laptop and communicates with the Kria board using ethernet. However, such workarounds are not ideal due to network communication overheads. Also, latency in the public-facing ReadUntil API provided by ONT (in Python programming language) would negate the massive benefit of hardware acceleration.

Our proof-of-concept HARU implementation is currently limited to DNA on R9.4 chemistry. Future work could focus on extending selective RNA sequencing, the most recent R10.4 chemistry, or upcoming protein sequencing from ONT.

The primary sequencer device targeted for HARU running on resource-constrained devices is the palm-sized MinION nanopore sequencer. Sequencers such as ONT’s PromethION provide a much larger throughput than MinION and will vastly increase the selective sequencing processing requirements. Future work could explore the scalability of HARU on higher-end FPGAs with high bandwidth memory and more resources for fast selective sequencing on high-throughput sequencers such as the PromethION.

### Related hardware acceleration work

Existing hardware acceleration work targeting the subsequence search problem using the DTW algorithm family is rare. Previous FPGA accelerators such as [49, 50] implement a cDTW accelerator to compute the distance score between a query and a window buffer that stores a subset of the reference sequence. The reference sequence is continuously streamed into the window after each cDTW compute iteration completes, shifting older samples out. A distance score that is below a preset threshold indicates a match between the query and the current reference subsequence in the window buffer. Other work [49] focused on exploiting coarse-grain parallelism by computing multiple cDTW in parallel, and [50] introduced a PE-ring structure that computes multiple recurrence equations in parallel where the PEs compute cells that do not share data dependencies. This windowed cDTW approach is suitable for reference sequences of undetermined arbitrary length. Still, it is inefficient (requires *N* × *O*(*M*^2^) for software approaches) for selective sequencing usage where the reference sequence is static with a known length. sDTW, on the other hand, is a data-reusing version of the approach, and our work exploits the fine-grain parallelism that computes the whole *O*(*M*) dimension in parallel, leaving *O*(*M* + *N*) computational time and *O*(*M*) space. Furthermore, there is prior work that accelerates DTW using nonvolatile memories [51] and using GPU acceleration [52, 53].

For the hardware acceleration on signal-alignment Read Until, the only previous attempt was a simulated Application Specific Integrated Circuit (ASIC) design that accelerates the sDTW algorithm [18]. The proposed accelerator uses the unprocessed raw signal reads to map directly with the reference, which requires 2,000 PEs to perform the matching and has a reference limit of 100 KB. The design has extensive resource requirements, making it difficult to fit on lower-cost reconfigurable hardware, thus targeting ASIC. Furthermore, as seen in the history of Read Until [11, 13, 27], Read Until requires implementations to adapt quickly as nanopore sequencing technology improves, and the cost of remanufacturing ASICs would be unsustainable. In contrast, HARU is a complete design with an efficient software processing layer using the sDTW accelerator. Our presented accelerator requires only 250 resource-efficient PEs due to preprocessing, reducing the query size needed in the high-throughput computation of sDTW, and is capable of executing selective sequencing with low-cost embedded MPSoC platforms with on-chip reconfigurable hardware.

## Conclusion

Existing sDTW-based software methods for nanopore selective sequencing are highly computationally intensive, and a large workstation cannot keep up with a portable MinION sequencer. In this article, we present HARU, a resource-efficient design that enables sDTW-based selective sequencing on a low-cost and portable heterogeneous system comprising an ARM processor and an FPGA, which is around 85× faster than the original sDTW-based software implementation and around 2.5× faster than a highly optimized software version running on a server with a 36-core Xeon processor for a complete SARS-CoV-2 dataset. The energy-delay product for the server is around 650× higher than HARU executing on an embedded device.

## Availability of Source Code and Requirements

### HARU

- Project name: HARU
- Description: Source code for the HARU accelerator, including the Verilog HDL core accelerator and user-space device driver
- Project homepage: https://github.com/beebdev/HARU
- Operating system(s): Windows 10/11 (building), Custom Embedded Linux image built with PetaLinux 2021.1 (running)
- Programming language: Verilog, C, Python
- Other requirements: Vivado 2022.2, Petalinux 2021.1
- License: MIT
- biotools:haru
- RRID: SCR_023563

### Sigfish-HARU

- Project name: Sigfish-HARU
- Description: Source code that demonstrates the proof-of-concept integration of HARU accelerator for squiggle mapping. Also contains the optimized RUscripts implementation in C.
- Project homepage: https://github.com/beebdev/sigfish-haru
- Operating system(s): Linux (building), embedded Linux built with PetaLinux 2021.1 (running)
- Programming language: C
- Other requirements: Cross-compilation toolchain for AARCH64
- License: MIT

### RUscripts-R9

- Project name: RUscripts-R9
- Description: The modified RUscripts to support Python 3.6+, BLOW5 format, and ONT’s current nanopore chemistry R9.4
- Project homepage: https://github.com/beebdev/RUscripts-R9
- Operating system(s): Platform independent
- Programming language: Python
- Other requirements: Python 3.6
- License: MIT

## Supplementary Material

giad046_GIGA-D-22-00317_Original_Submission

giad046_GIGA-D-22-00317_Revision_1

giad046_GIGA-D-22-00317_Revision_2

giad046_GIGA-D-22-00317_Revision_3

giad046_Response_to_Reviewer_Comments_Original_Submission

giad046_Response_to_Reviewer_Comments_Revision_1

giad046_Response_to_Reviewer_Comments_Revision_2

giad046_Reviewer_1_Report_Original_Submission Jason Lau -- 12/22/2022 Reviewed

giad046_Reviewer_2_Report_Original_Submission Mohammed Alser -- 12/25/2022 Reviewed

giad046_Reviewer_2_Report_Revision_1 Mohammed Alser -- 4/29/2023 Reviewed

giad046_Reviewer_3_Report_Original_Submission Daichi Fujiki -- 12/26/2022 Reviewed

giad046_Reviewer_3_Report_Revision_1 Daichi Fujiki -- 5/6/2023 Reviewed

giad046_Reviewer_4_Report_Original_Submission Bertil Schmidt -- 12/31/2022 Reviewed

giad046_Supplemental_File

## Data Availability

Datasets used for the benchmarks are available to be directly downloaded via the *Zenodo* repository, *Zenedo* [54], which we curated from publicly available datasets: [55] (associated with publication [45]) and NCBI accession SRX11368475 (associated with publication [41]). An archival copy of the code and supporting data is also available via the *GigaScience* repository, GigaDB [56].

## Additional Files

**Supplementary Fig. S1.1**. DTW score cutoff threshold versus accuracy.

**Supplementary Fig. S1.2**. DTW score distribution of positive and negative mappings.

**Supplementary Fig. S2**. Impact of query length and prefix trim length on accuracy.

**Supplementary Fig. S3**. sDTW accelerator modeled using 32-bit floating point.

**Supplementary Fig. S4**. sDTW accelerator modeled using 32-bit fixed point.

**Supplementary Fig. S5**. sDTW accelerator modeled using 16-bit fixed point.

**Supplementary Fig. S6**. Postimplementation power-analysis information of Kria with a single accelerator HARU.

**Supplementary Table S1**. Post-implementation utilisation for HARU system with single dynamic reference streaming accelerator.

**Supplementary Table S2**. Postimplementation utilization for HARU system with 4 dynamic reference streaming accelerators.

**Supplementary Table S3**. Comparison between HARU and DeepSelectNet.

**Supplementary Table S4**. DeepSelectNet mapping results of SARS-CoV-2 and yeast.

**Supplementary Table S5**. Comparison between HARU and Guppy_fast + Minimap2.

**Supplementary Table S6**. Guppy_fast + Minimap2 mapping results of SARS-CoV-2 and yeast.

**Supplementary Table S7**. Comparison between HARU and UNCALLED.

**Supplementary Table S8**. Resource utilization for SquiggleFilter’s submodules.

**Supplementary Table S9**. Resource utilization for Kria with HARU deployed in programmable logic (PL).

**Supplementary Table S10**. Resource utilization of HARU’s sDTW accelerator (experimental reference streaming).

## Abbreviations

API: Application Programming Interface; ASIC: Application-Specific Integrated Circuit; AXI: Advanced eXtensive Interface; cDTW: classical dynamic time warping; CLB: configurable logic block; CPU: central processing unit; DDR: double data rate; DMA: direct memory access; DTW: dynamic time warping; FPGA: field-programmable gate array; GPU: graphics processing unit; HDL: Hardware Descriptive Language; HPC: high-performance computing; LUT: lookup table; MAPQ: mapping quality; MPSoC: multiprocessor system-on-chip; ONT: Oxford Nanopore Technologies; PE: processing element; RAM: random access memory; sDTW: subsequence dynamic time warping; TDP: thermal design power.
