# Supplementary material for: Efficient real-time selective genome sequencing on resource-constrained devices
Source: Gigascience. 2023 Jul 3;12:giad046. doi: 10.1093/gigascience/giad046 (PMC10316692; doi:10.1093/gigascience/giad046)
Supplement: giad046_GIGA-D-22-00317_Original_Submission [file giad046_giga-d-22-00317_original_submission.pdf]

GigaScience

Efficient Real-Time Selective Genome Sequencing on Resource-Constrained Devices

--Manuscript Draft--

Manuscript Number:	GIGA-D-22-00317
Full Title:	Efficient Real-Time Selective Genome Sequencing on Resource-Constrained Devices
Article Type:	Technical Note
Funding Information:	
Abstract:	Third-generation nanopore sequencers offer a feature called selective sequencing or `Read Until' that allows genomic reads to be analyzed in real-time and abandoned halfway if not belonging to a genomic region of `interest'. This selective sequencing opens the door to important applications such as rapid and low-cost genetic tests. The latency in analyzing should be as low as possible for selective sequencing to be effective so that unnecessary reads can be rejected as early as possible. However, existing methods that employ subsequence Dynamic Time Warping (sDTW) algorithm for this problem are too computationally intensive that a massive workstation with dozens of CPU cores still struggles to keep up with the data rate of a mobile phone-sized MinION sequencer. In this paper, we present Hardware Accelerated Read Until (HARU), a resource-efficient hardware-software co-design-based method that exploits a low-cost and portable heterogeneous Multiprocessor System-on-Chip (MPSoC) platform with on-chip Field-Programmable Gate Arrays (FPGA) to accelerate the sDTW-based Read Until algorithm. Experimental results show that HARU on a Xilinx FPGA embedded with a 4-core ARM processor is around 2.5X faster than a highly optimized multi-threaded software version (around 85X faster than the existing unoptimized multi-threaded software) running on a sophisticated server with 36-core Intel Xeon processor for a SARS-CoV-2 dataset. The energy consumption of HARU is two orders of magnitudes lower than the same application executing on the 36-core server. The source code for HARU sDTW module is available as open-source at https://github.com/beebdev/HARU, and an example application that utilizes HARU is at https://github.com/beebdev/sigfish-haru.
Corresponding Author:	Hasindu Gamaarachchi<br>Garvan Institute of Medical Research<br>Darlinghurst, AU-NSW AUSTRALIA
Corresponding Author Secondary Information:	
Corresponding Author's Institution:	Garvan Institute of Medical Research
Corresponding Author's Secondary Institution:	
First Author:	Po Jui Shih
First Author Secondary Information:	
Order of Authors:	Po Jui Shih
	Hassaan Saadat
	Sri Parameswaran
	Hasindu Gamaarachchi
Order of Authors Secondary Information:	
Additional Information:	
**Question**	**Response**
Are you submitting this manuscript to a special series or article collection?	No
**Experimental design and statistics**	Yes

Powered by Editorial Manager® and ProduXion Manager® from Aries Systems Corporation

Full details of the experimental design and statistical methods used should be given in the Methods section, as detailed in our Minimum Standards Reporting Checklist. Information essential to interpreting the data presented should be made available in the figure legends.<br><br>Have you included all the information requested in your manuscript?	
**Resources**<br><br>A description of all resources used, including antibodies, cell lines, animals and software tools, with enough information to allow them to be uniquely identified, should be included in the Methods section. Authors are strongly encouraged to cite Research Resource Identifiers (RRIDs) for antibodies, model organisms and tools, where possible.<br><br>Have you included the information requested as detailed in our Minimum Standards Reporting Checklist?	Yes
**Availability of data and materials**<br><br>All datasets and code on which the conclusions of the paper rely must be either included in your submission or deposited in publicly available repositories (where available and ethically appropriate), referencing such data using a unique identifier in the references and in the "Availability of Data and Materials" section of your manuscript.<br><br>Have you have met the above requirement as detailed in our Minimum Standards Reporting Checklist?	Yes

Powered by Editorial Manager® and ProduXion Manager® from Aries Systems Corporation

Manuscript

Click here to access/download;Manuscript;HARU_gigascience.pdf

GigaScience, 2022, 1–10

doi: xx.xxxx/xxxx
Manuscript in Preparation
Paper

PAPER

Efficient Real-Time Selective Genome Sequencing on Resource-Constrained Devices

Po Jui Shih[1,*], Hassaan Saadat[1], Sri Parameswaran[1] and Hasindu Gamaarachchi[2,1,†]

[1]School of Computer Science and Engineering, UNSW Sydney, Australia and [2]Kinghorn Centre for Clinical Genomics, Garvan Institute of Medical Research, Sydney, Australia

[*]pojui.shih@unsw.edu.au
[†]hasindu@garvan.org.au

Abstract

Third-generation nanopore sequencers offer a feature called selective sequencing or 'Read Until' that allows genomic reads to be analyzed in real-time and abandoned halfway if not belonging to a genomic region of 'interest'. This selective sequencing opens the door to important applications such as rapid and low-cost genetic tests. The latency in analyzing should be as low as possible for selective sequencing to be effective so that unnecessary reads can be rejected as early as possible. However, existing methods that employ subsequence Dynamic Time Warping (sDTW) algorithm for this problem are too computationally intensive that a massive workstation with dozens of CPU cores still struggles to keep up with the data rate of a mobile phone-sized MinION sequencer. In this paper, we present Hardware Accelerated Read Until (HARU), a resource-efficient hardware-software co-design-based method that exploits a low-cost and portable heterogeneous Multiprocessor System-on-Chip (MPSoC) platform with on-chip Field-Programmable Gate Arrays (FPGA) to accelerate the sDTW-based Read Until algorithm. Experimental results show that HARU on a Xilinx FPGA embedded with a 4-core ARM processor is around 2.5× faster than a highly optimized multi-threaded software version (around 85× faster than the existing unoptimized multi-threaded software) running on a sophisticated server with 36-core Intel Xeon processor for a SARS-CoV-2 dataset. The energy consumption of HARU is two orders of magnitudes lower than the same application executing on the 36-core server. The source code for HARU sDTW module is available as open-source at https://github.com/beebdev/HARU, and an example application that utilizes HARU is at https://github.com/beebdev/sigfish-haru.

Key words: selective sequencing; adaptive sampling; nanopore; subsequence dynamic time warping; FPGA; hardware acceleration; edge computing;

Introduction

The latest third-generation nanopore sequencing technology has revolutionized the field of genomics. The portable palm-sized nanopore sequencer called the MinION produced by Oxford Nanopore Technologies (ONT) can perform direct selective sequencing, which rejects the genomic reads that are not of interest. This technique, also known as Read Until, can vastly reduce the sequencing time and cost for applications such as genetic disease identification [1, 2], cancer detection [3, 4], the surveillance of viruses (e.g. SARS-CoV-2) and other pathogens [5, 6], and sequencing low abundance species metagenomics samples [7]. However, the real-time analysis of genomic reads involves aligning the read to the reference to obtain the position information, which is a complex and time-consuming process. Existing alignment methods used in selective sequencing either directly map the sequenced raw signals using algorithms such as the subsequence dynamic time warping (sDTW) [8] or convert the raw signals into base sequenced before using base-domain alignment methods [9, 10].

Previous sDTW-based Read Until, running on a 22-core High-Performance Computing (HPC) system, [8] could not keep up with

Compiled on: November 19, 2022.
Draft manuscript prepared by the author.

Key Points

- Hardware accelerated signal-matching Read Until designed to perform high throughput sequence mapping on resource-constrained embedded platforms.
- A resource-efficient sDTW accelerator for selective sequencing that reduces space complexity from $O(MN)$ to $O(M)$ while exploiting fine-grain parallelism to reduce the computational time from $O(MN)$ in software to $O(M + N)$ in hardware.
- The full proposed design, including the software processing layer, devices drivers, and the hardware sDTW accelerator developed during this research, is released as open-source at `https://github.com/beebdev/HARU` and an example application that utilizes HARU is at `https://github.com/beebdev/sigfish-haru`.

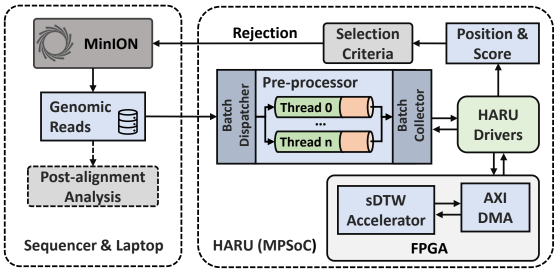

Figure 1. HARU overview.

the current 450 bases/s sequencing rate [11] of the portable palm-sized MinION sequencer and we found that the sDTW computation takes more than 98% of its run time. Current base-aligning Read Until implementations [10] use GPU-accelerated basecallers to convert signal reads to bases and map with the reference base sequence using optimized sequence mapping techniques (e.g. *Minimap2* [12]) that are mature in the bio-informatics field. Although enough to keep up with the sequencing rate, the execution requires high-end GPU hardware (NVIDIA RTX 1080 for simple reference targets [10] and NVIDIA RTX 3090 for more complex targets [1]), which makes selective sequencing expensive and non-portable.

To address the lack of portability and costly execution nature of existing implementations, we return to raw signal mapping Read Until and tackle the computational bottleneck by accelerating the sDTW algorithm with Field-Programmable Gate Arrays (FPGAs). Hardware acceleration work in the past for the sDTW algorithm is rare, and the $O(MN)$[1] space complexity of the algorithm does not naively fit on reconfigurable hardware efficiently. For the use case of selective sequencing, the $M \times N$ memory is unnecessary, and the required results can be obtained with $O(M)$ space complexity. We designed a memory-efficient sDTW accelerator for Read Until and exploited fine-grain parallelism to reduce the computational time complexity to $O(M + N)$.

In this paper, we propose HARU (see Figure 1), a software-hardware co-design for raw signal-alignment Read Until that utilizes the memory-efficient sDTW accelerator for high throughput signal mapping. HARU targets low-cost heterogeneous Multiprocessor System-on-Chip (MPSoC) devices with on-chip reconfigurable hardware and performs efficient multi-threaded batch-processing for the signal preparation in conjunction with the sDTW accelerator. We demonstrate that our design is capable of keeping up with the data rate of the ONT's MinION sequencer and gains around 85× speedup against the original software implementation mapping the SARS-CoV-2 sequenced data on a 36-core HPC system. Furthermore, we show that our work runs around 2.5× faster than an optimized multi-threaded software implementation on the same

1 M is the size of the query and N is the size of the reference sequence

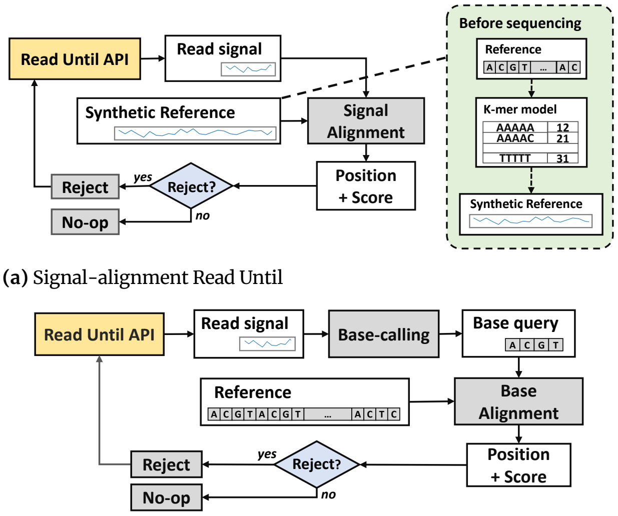

(a) Signal-alignment Read Until

(b) Base-alignment Read Until

Figure 2. Overview of Read Until workflows

36-core server and around 6.5× faster than the same software running on a 10-core Intel Core i9-10850K desktop. Multiple instances of the system running on low-cost MPSoC can be parallelized to potentially scale up processing and run on sequencers of a larger scale (e.g. ONT's PromethION).

Background

Nanopore Selective sequencing

Nanopore sequencers from ONT are third-generation genomic sequencers that are capable of producing long reads (currently ranging between 1 kilo-bases to >2 mega-bases) [13, 14] and are commercially available at an affordable price compared to sequencers of other techniques and generations [15]. These ONT nanopore sequencers provide genomic reads through *flow cells* which contain a proprietary sensor array over nanopore channels embedded in a synthetic membrane [16]. During the sequencing process, the nanopore channels capture the electric current change caused by the genome molecules' ionic current when it passes through [16]. This current signal trace is streamed to the sequencer software in real time and can later be basecalled into the corresponding nucleobase representation for later analysis [17].

A feature of ONT nanopore sequencers is the direct selective sequencing capability. These sequencers provide real-time data output streams and allow the rejection of reads at individual nanopore channels [8, 10]. This means the sequenced data can be analyzed during the sequencing and rejected before completion if decided it is not of interest. This selective sequencing process in the nanopore sequencing workflow is known as *Read Until*. ONT provides the Read Until API interface for software applications to access and re-

ject the sequenced reads in real-time. A rejection made through the Application Programming Interface (API) call will eventually be passed back to the sequencer. The voltage at the indicated channel will be reversed to eject the genomic molecule out of the nanopore [8].

For the Read Until execution to be effective, the round-trip task latency for read acquisition, analysis, and rejection signal forwarding should be completed before the majority of the subject read is sequenced by the nanopore sequencer [8]. Rejections made after most of the strand is sequenced bring no benefit as no sequencing time is saved. Existing Read Until methods performs analysis by aligning the genomic reads to the target reference and making the rejection decision based on the position and distance score. This alignment can be done using either signal or base alignment [8, 18, 19, 10, 9, 20].

Signal-alignment Read Until. Signal-alignment Read Until aligns raw signal reads with the reference to obtain the alignment position and distance score, as seen in Figure 2a. Reference sequences usually are obtained in base representation (in the base equivalent '*ACGT*' characters) and need to be converted to a synthetic signal representation before being used to map the reads. This can be done using the k-mer model, which slides a window size of k bases over the base reference while the bases in the window are mapped to a value using the k-mer model hash-map (see Figure 2a). The obtained alignment position and score are then used to determine if a rejection should be made, which is custom to application usage. This signal-alignment workflow was first shown by Loose et al. [8] in the *RUscripts* work, which is also the first Read Until implementation introduced. *RUscripts* is a Python implementation that uses the sDTW algorithm to align initial segments of the raw signals to the synthetic reference and can match 1 read every 0.3 seconds on a single CPU core [8]. At the time of the proposal, *RUscripts* could keep up with the 70 bases/s nanopore sequencing rate on a 22-core server [8]. However, as sequencing speed improved over the years, the current 450 bases/s sequencing rate [11] surpassed *RUscripts*'s capability of performing Read Until during sequencing. We observed that 98% of processing time is spent processing the $O(MN)$ sDTW algorithm.

Base-alignment Read Until. As signal-aligning Read Until could not keep up with improved sequencing rates due to sDTW, researchers turned the focus of Read Until workflows towards base-domain techniques [9, 10]. These techniques align the genomic reads in the base domain as opposed to the signal domain, which requires an extra step of basecalling the signal to base sequences in real-time before alignment (see Figure 2b). Thanks to well-optimized multi-state alignment implementations such as *Minimap2* [12] and the proprietary GPU-accelerated basecaller *Guppy* from ONT, it can out-speed sequencing rate to save time. Yet, the extensive power usage and the need for high-performance GPUs and CPUs make base-alignment Read Until expensive and non-portable [1].

Potential for signal-alignment Read Until. The above two alignment methods share high similarities in their algorithms and mainly differ in the sequence representation [21]. Since base-alignment Read Until can perform selective sequencing under current sequencing rates [10, 1] with the extra base-calling step, we revitalize the direct signal approach by optimizing and exploiting hardware acceleration for the sDTW alignment methodology targeting low-cost embedded heterogeneous platforms which also addresses the high cost of Read Until executions.

Subsequence Dynamic Time Warping

The dynamic time warping algorithm family are dynamic programming algorithms that provide optimal alignment and distance metrics between two given time series [22] and have been widely used in pattern recognition applications in different fields [23, 24]. This

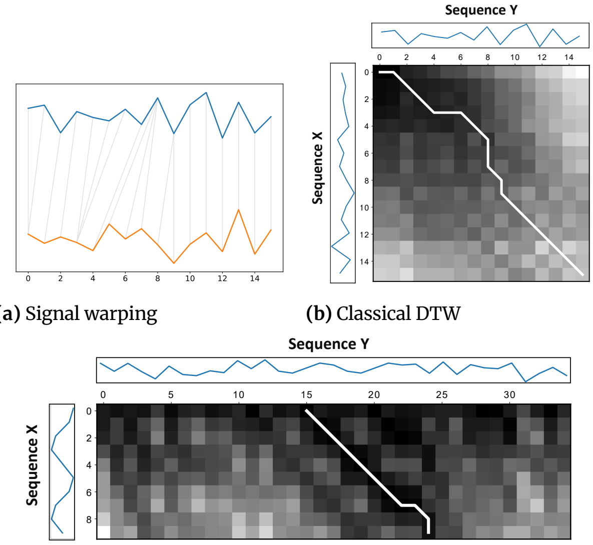

(a) Signal warping **(b)** Classical DTW

(c) Subsequence DTW

Figure 3. Illustration of DTW

optimal alignment is achieved by warping the time series samples (see Figure 3a), which is done by keeping an $M \times N$ sized cost matrix. The classical DTW (cDTW) algorithm performs global alignment of the signals (see Figure 3b) [22], while the sDTW algorithm performs local alignment of the smaller sequence in the larger sequence (see Figure 3c) [25]. Read Until attempts to find the local alignment of the query on the reference and thus utilizes sDTW, which is elaborated below:

sDTW Problem: Given two sequences X of size M and Y of size N where $1 \leq M \leq N \in \mathbb{N}$, the sDTW distance is the summation of the distance in the optimal warp path $w_{optimal}$. The warp paths considered are all the paths that align the sequence X with any subsequence of the sequence Y. The dynamic programming formulation of sDTW is based on the recurrence relation of the equation:

$$\gamma(i,j) = \delta(i,j) + min \begin{cases} \gamma(i-1,j) \\ \gamma(i-1,j-1) \\ \gamma(i,j-1) \end{cases} \quad (1)$$

where δ is the distance measure[2] between samples and $1 \geq i \geq M$, $1 \geq j \geq N$. The boundary conditions for $\gamma(i,j)$ include $\gamma(i,0) = \infty$ and $\gamma(0,j) = 0$ and with a bottom-up memoisation, the γ values are stored in a cost matrix C of size $N \times M$ (i.e., $C[i,j] := \gamma(i,j)$). γ essentially chooses, at each step, the lowest cost move[3]. Once the cost matrix C is populated, the cell with the minimum distance value in the last row would be the ending position of the local alignment. Backtracking from the end position by, again, choosing the step with the lowest cost among the same dependency will give the optimal warp path and starting position (see Figure 3c).

Time and space complexity: The sDTW approach is given in Algorithm 1. As shown, sDTW is $O(MN)$ in time and space complexity due to the 2-dimensional search space. This has led to heavy computational bottlenecks in applications such as *RUscripts* discussed in Section "Nanopore Selective sequencing". To date, not many sDTW optimization methods exist, and cDTW optimizations such as lower bounding [26, 27] and applying global constraints [28, 29] do not bring much benefit as the necessary search space is much larger than just the diagonal connecting start and end positions of the sequences.

2 Distance metrics in DTW are not limited to a single method. Popular distance metrics include Euclidean distance, squared Euclidean distance, and Manhattan distance.

3 In Equation 1, $\gamma(i-1,j)$ indicates an *insertion* from sequence X into sequence Y whereas $\gamma(i-1,j-1)$ indicates a *match* and $\gamma(i,j-1)$ indicates a *deletion*.

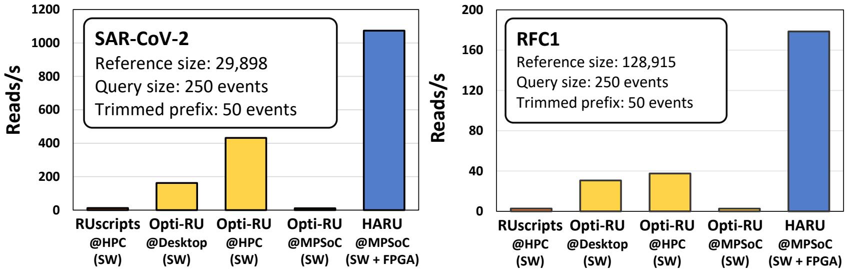

(a) SARS-CoV-2 dataset (b) RFC1 dataset

Figure 4. Mapping throughput for the selective sequencing

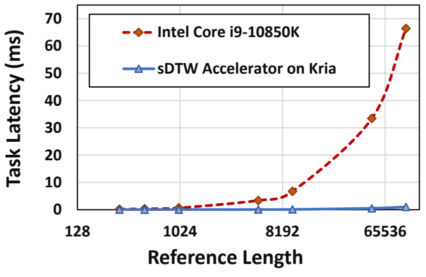

Figure 5. sDTW task latency

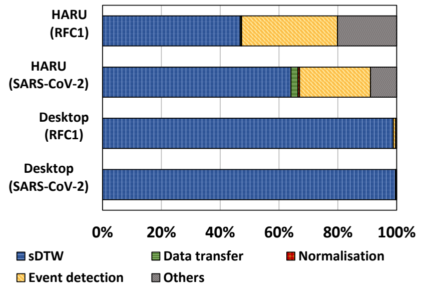

Figure 6. Process time breakdown

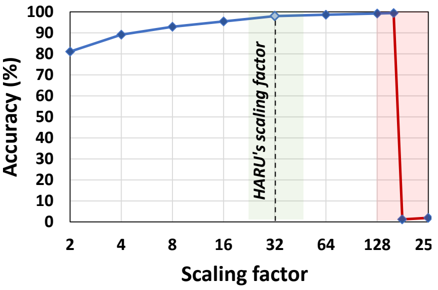

Figure 7. Accuracy against scaling factor

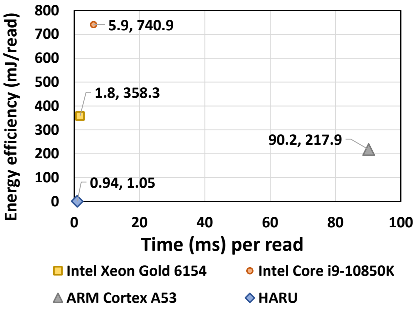

Figure 8. Energy and performance

Algorithm 1: Subsequence DTW

```
Input  : X[1 : M], Y[1 : N], M, N
Output : position, score
C: cost matrix of size N * M;
score ← ∞;
position ← −1;
for j in range 1 to N do
  | C[1, j] ← abs(X[i] − Y[0]);
end
for i in range 1 to M do
  | C[i, 1] ← abs(X[i] − Y[0]) + C[i − 1, 1];
end
for i in range 1 to M do
  | for j in range 1 to N do
  |   | d ← min(C[i − 1, j], C[i, j − 1], C[i − 1, j − 1]);
  |   | C[i, j] ← abs(X[i] − Y[j]) + d;
  | end
end
for j in range 1 to M do
  | if C[N, j] < min_score then
  |   | position ← j;
  |   | score ← C[N, j];
  | end
end
```

Results

Overall system performance

Figure 4a compares the overall performance of HARU for mapping all the 1.382 million reads of the SARS-CoV-2 dataset (see section "Datasets") with software-only implementations. The y-axis of Figure 4a is the signal mapping throughput (mapping throughput is the execution time divided by the number of reads in the dataset). The First bar in Figure 4a represents the original Python-based *RUScripts* (see section "Pure software implementations") run on the HPC with all 36 cores (throughput: 12.52 reads/s). The last bar represents our HARU system with a throughput of 1073.83 reads/s). Thus, our HARU system is ~85.8× faster than the original *RUscripts*. The second bar shows the optimized C implementation of *RUScripts* (see section "Pure software implementations") on the desktop system with a 10-core i9 processor, and the throughput is 162.29 reads/s (HARU is 6.6× faster). Then, the third bar is for the optimized C implementation run with all 36 Xeon cores on the HPC, and the throughput is 432.06 reads/second. HARU system being implemented on a low-cost embedded FPGA system is still ~2.49× faster than the server. The fourth bar in Figure 4a is for the optimized C implementation on the MPSoC run only on the 4-core ARM CPU, which has a throughput of 11.09 read/second. Thus, HARU that utilizes the FPGA is 96.8× faster than running on the ARM processor alone.

Similarly, Figure 4b compares the overall HARU performance for mapping all the 500,000 reads of the human dataset to the reference containing the RFC1 gene (see section "Datasets"). HARU (last bar) is 64.5× faster than *RUScripts* on the 36-core HPC (first bar); 5.8× and 4.7× faster than optimized C implementation on the 10-core desktop (second bar) and 36-core HPC (third bar), respectively; and, 66.2× than the optimized C implementation on a 4-core ARM processor (fourth bar) alone.

Note that time measurement for the above throughput calculation for HARU includes all the overheads, including reading signal data from the disk, raw signal pre-processing on software, and data transfer time to/from FPGA for HARU and our FPGA implementation is running at 100 MHz. The speedups observed for HARU over other systems in Figure 4a (SARS-CoV-2 reference) are higher compared to those in Figure 4b (RFC1 reference) because the RFC reference is larger (128Kbases) than the SARS-CoV-2 reference (29Kbases) as explained below.

Performance of the sDTW over reference length

Figure 5 shows how the performance of our sDTW core in HARU executed on the FPGA (including the overhead for data transfer to/from FPGA), and the pure software version of DTW executed on the CPU varies over the reference length. The X-axis is the reference length on a number of bases on the log scale. Y-axis is the time taken for a single sDTW query. For the CPU (red curve), where this y-axis represents the time for executing the sDTW function on a single CPU thread, whereas, for the FPGA (blue curve), this is the time for processing on the FPGA plus the data transfer to and from the FPGA. Observe in Figure 5 how the gap between the two curves increases with the reference length, which causes the speed up of HARU over CPU to increase with increased reference size. This behavior is due to a band of cells being computed in parallel on hardware using a Processing Elements (PE) chain (see section "Resource-Efficient

Table 1. sDTW Accelerator resource utilization

Resource	Available	Used (utilisation)
**CLB LUT**	117,120	21,570 (18.42%)
**CLB Registers**	234,240	16,796 (7.17%)
**F7 Muxes**	58,560	9 (0.02%)

sDTW Accelerator").

The time breakdown for different processing steps

Figure 6 compares the percentage of time spent on different processing steps for HARU vs the optimized software implementation in percentage. Due to the significant speedup of sDTW, the percentage of run time spent on sDTW is <64% for the SARS-CoV-2 dataset and >46% for the RFC1 data set (top two bars), whereas this was >98% for software (bottom two bars). Note that 'others' in Figure 6 is the time spent loading data from the disk, reference preparation, and writing the output.

Accuracy

Figure 7 shows the accuracy of the accelerator using different scaling factors (discussed in section "Software Processing Layer"). Accuracy in Figure 7 is calculated as a percentage of the number of mapping positions similar to results produced from sDTW computed on software using 32-bit floating-points. Observe that a scaling factor of 2 yields a limited accuracy (80%), while increasing the scaling factor gradually converges the accuracy towards 100%. However, when scaled above 128, the distance cost accumulation results in data overflow during sDTW, which largely impacts the alignment accuracy. In HARU, we have used a scaling factor of 32 to prevent overflow while having an accuracy close to 99%.

Energy comparison

Figure 8 shows the estimated energy efficiency (y-axis) plotted against the execution time (x-axis) for HARU and optimized software-only implementations on different processors. HARU's overall performance and energy efficiency are considerably lower (close to the origin of the graph: time 0.94 ms/read and energy 1.05 mJ/read) than the optimized version running on ARM (90.2 ms/read, 217.9 mJ/read), Intel Core-i9 (5.9 ms/read, 740.9 mJ/read), and Intel Xeon Gold processor (1.8 ms/read, 358.3 mJ/read). The energy-delay product for the server is 644.94, whereas 0.987 for HARU. Thus, HARU is 650X better in terms of energy-delay products. The energy consumed for HARU and the ARM processor was estimated using the power estimates reported by Vivado in the synthesis report. In contrast, the Thermal Design Power (TDP) value reported in the processor specification was used for Intel processors.

Resource utilization

Resource utilization for our sDTW accelerator with a single query processor on the Kria board, as reported by the Vivado synthesis report, is shown in Table 1. Note that we used a single query processor for all the above experiments to show the bare minimum performance on a low-end FPGA platform. As shown in Table 1, the maximum utilization (CLB LUT) is <20%; thus, the Kria board can support up to five parallel query processors if necessary.

Methods

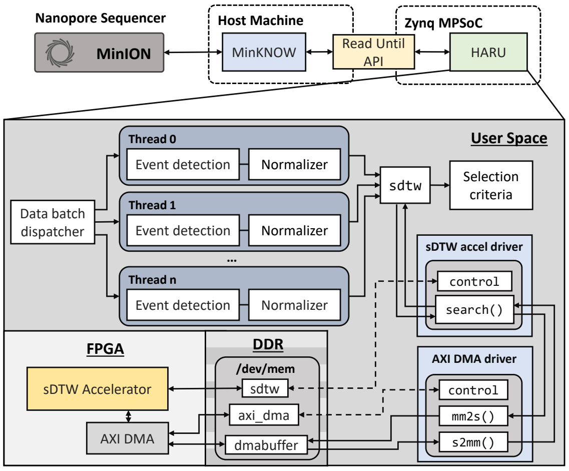

Figure 9. HARU architecture.

Design of Hardware Accelerated Read Until

HARU targets low-cost MPSoCs with on-chip FPGA to perform selective sequencing processing. Figure 9 shows the architecture of HARU in an ONT nanopore sequencing workflow. HARU consists of three main components: the software processing layer, device drivers for the accelerator and associated hardware, and the hardware sDTW accelerator. The software processing layer, discussed in subsection "Software Processing Layer", uses a multi-threaded batch processing architecture to perform raw read signal pre-processing and is customizable based on the selection criteria. The device drivers, discussed in subsection "HARU Device Drivers", are designed to provide high-throughput data transferring of query and reference signals. Lastly, the resource-efficient sDTW accelerator, discussed in subsection "Resource-Efficient sDTW Accelerator", performs high throughput sDTW for the selective sequencing use case.

Software Processing Layer

The software processing layer of HARU is the front end of the HARU design running on the processing system on the MPSoC. Its main tasks include pre-processing the reference sequence and raw signal reads and the final selection decision. Since references are obtained in base representation as discussed in subsection "Nanopore Selective sequencing", the initialization step of the software processing layer forms the synthetic reference signal for the forward and reverse representation of the base reference[4] using the k-mer model for the flowcell type. Then, in preparation for the sDTW computation in hardware, the reference signal is normalized using z-score normalization. Since the data type used for the signal and cost matrix in the sDTW accelerator are 16-bit fixed-point types (discussed in section "Resource-Efficient sDTW Accelerator"), the normalized values are scaled with a scaling factor to preserve signal resolution.

During the genome sequencing step, the software layer collects sequenced data from the nanopore sequencer in batches which is then dispatched into multiple threads for efficient computing of pre-processing (see Figure 9). Each thread performs event detection on the raw signal samples to reduce sample data size for the sDTW accelerator. This is done until enough events are collected. We find that for the R9.4 flowcell, 250 events are adequate for mapping and would require roughly 0.67 seconds of data collection[5]. After the collection, the events are normalized and scaled with the same scaling factor used in the reference signal preparation. When threads finish the pre-processing, the processed data are gathered

4 This is needed since DNA molecules are double-stranded.
5 time to obtain around 50 events that belong to the read adaptor and then actual 250 events of the query.

Algorithm 2: Memory-efficient subsequence DTW

```
   Input  : X[1 : M], Y[1 : N], M, N
   Output : position, score
1  C: array of size M + 1 initialised to ∞;
2  score ← ∞;
3  position ← −1;
4  for j in range 1 to N do
5      n ← 0;
6      nw ← C[1];
7      w ← C[2];
8      for i in range 1 to M do
9          C[i] := abs(x[i] − y[j]) + min(n, nw, w);
10         n := C[i];
11         nw := w;
12         w := C[i + 2];
13     end
14     if C[M] < score then
15         position ← j;
16         score ← C[M];
17     end
18 end
```

and sent to the sDTW accelerator for processing using the drivers. After which, the mapping position and the similarity score are used to decide whether the read should be rejected.

HARU Device Drivers

To control and utilize the hardware accelerator in the software processing layer, we designed the software device drivers to have two main data paths (see Figure 9). The first data path is the control path of the accelerator, which uses the AMBA AXI4-Lite protocol to configure the control registers and read status registers in the software. The accelerator's physical address is memory-mapped to the virtual address space for user space applications to utilize.

The second data path is for the query and reference sequences. To prevent data transfer from becoming a bottleneck, we use the AMBA AXI4-Stream protocol to stream query and reference data into the accelerator at a high-throughput rate. This is done by using AXI Direct Memory Access (DMA) module to point to a physical hardware address to stream data to and from. By calling the driver function for processing the query, the sDTW accelerator driver initiates the transfer from the query and reference buffers to the transfer buffer on Double Data Rate (DDR) memory dedicated to AXI-stream communication and the FPGA. Our benchmarks show that data can be sent to and from the accelerator at a throughput of 330 MB/s.

Resource-Efficient sDTW Accelerator

As discussed in section "Subsequence Dynamic Time Warping", the standard sDTW algorithm has $O(MN)$ time and space complexity due to the computation of the cost matrix. The computation of a cell value in the cost matrix requires comparing three neighbor cell values, making the exploitation of available hardware parallelism harder. Also, the preservation of the full cost matrix does not scale well if directly implemented on resource-constrained FPGA devices. We identified that the backtracking of the cost matrix to obtain the warp path is unnecessary for Read Until as the ending position is adequate to make the rejection decision. We provide the following optimizations over sDTW to obtain a resource-efficient high-throughput sDTW accelerator.

Cost matrix memory optimization. The need to preserve the $M \times N$ sized matrix for backtracking was discussed in section "Subsequence Dynamic Time Warping". However, for selective sequencing, the obtained end position of the alignment is adequate to determine the location of the current query; thus, the backtracking step for obtaining the starting position is unnecessary. Consequently, preserving the whole cost matrix values is unnecessary, and a cost array of $M + 1$ is sufficient. Algorithm 2 shows the sDTW algorithm after the cost matrix size is reduced. The outer loop (line 4 of Algorithm 2) iterates through the whole reference sequence, while the nested inner loop (line 8 of Algorithm 2) iterates through the

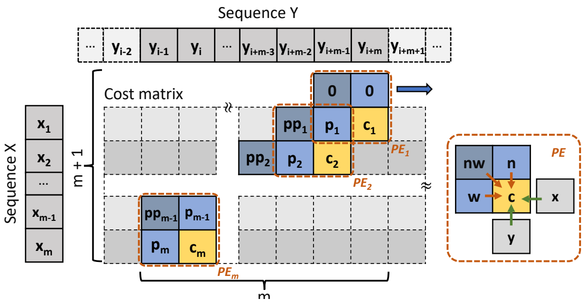

Figure 10. Pipelined execution of Algorithm 2

column at each reference sample. During each iteration of the inner loop, the computation of the recurrence equation is performed, and the computed value is stored in the cost array that is of the same size as the query. Once the inner loop completes, the current minimum score and position values are updated if the last cell of the cost matrix is smaller than the current minimum score.

Operation pipelining. The sDTW cost matrix size reduction explained above optimizes the space complexity of the computation for selective sequencing. However, the algorithm's execution is still sequential and has $O(MN)$ time complexity. Computing the whole column in parallel by unrolling the inner loop is not feasible due to the data dependency in the recurrence equation that needs waiting until the n value is ready (see Algorithm 2). We observe that once the first iteration of the inner loop for the column is completed, all data dependencies for the first inner loop iteration for the next column are ready. By pipelining the outer loop computation, an oblique column is formed that is computed in parallel, as shown in Figure 10. This oblique column traverses through the reference sequences, reducing the time complexity from $O(MN)$ to $O(N)$ since the N query size is now computed in parallel.

Fixed-point data representation. After the optimisation above, the hardware's computational complexity is $O(M)$. However, the actual time needed is $(M + N - 1) \times II$, where II is the initiation interval (i.e. the number of cycles between loop iterations). In pipelined Algorithm 2, II is how fast the reference equation $C[i] := abs(x[i] - y[j]) + min(n, nw, w)$ can be computed. Normally, 32-bit floating-point data types are used for the sDTW computation to preserve the precision after the sequences are normalized. This is expensive to implement in hardware regarding resources and execution time. By using a fixed-point representation with fewer data bits and scaling the sequence values using a scaling factor, the recurrence equation can be computed in hardware rapidly and efficiently while keeping sufficient precision. We chose 16-bit fixed-points with a scaling factor of 2^5 as it gives sufficient precision and keeps II at one clock cycle (see section 21 on accuracy). Note that a 32-bit data type is used to accumulate distance scores in cost arrays to avoid overflow.

HARU's sDTW Accelerator. The oblique parallel-computed column mentioned above uses a PE-chain structure where data-dependent neighbor cells are shared amongst the PEs (Figure 10). As shown in Figure 11, the shared values are stored in two register arrays of size M (L1 being the previous cost array and L2 being the second previous cost array). At each iteration, the costs in the L1 array are shifted into the L2 array, while the current costs are passed onto the L1 array. Each PE computes the recurrence equation, which takes the Manhattan distance ($\delta = |x[i] - y[j]|$) and adds the minimum of the three neighbor cells (see equation 1). Samples of the reference sequence are first streamed into the first PE of the chain and are then passed along to successive PEs in each iteration. In the subsection "Software Processing Layer", we discussed that the software processing layer uses multi-threaded batch processing to perform event detection and normalization. The event detection decreases the query size to make the M term smaller in the algorithm complexity. We choose to use a size of 250 events, giving the accelerator a PE chain of 250 PEs. In total, it takes $N + 250 - 1$ clock

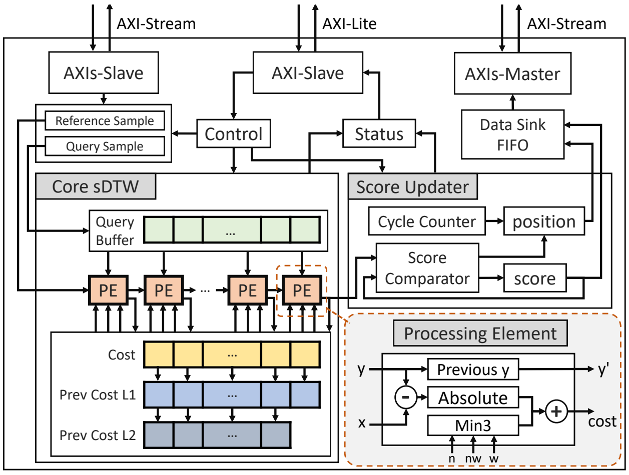

Figure 11. sDTW hardware accelerator design for HARU

Table 2. Computational platforms

System	HPC	Desktop	MPSoC
**CPU**	Intel Xeon Gold 6154	Intel Core i9-10850K	Arm Cortex-A53
**CPU cores**	36	10	4
**Clock rate**	3.00 GHz	3.60 GHz	1.5 GHz
**RAM**	377 GB	32 GB	4 GB
**FPGA**	No	No	Yes

Table 3. Datasets

Target	SARS-CoV-2	RFC1
**Type**	Viral genome	Partial human genome
**No. of bases**	29,903	128,915
**Search space size**	59,806	257,830
**No. of reads**	1,382k	500k
**SLOW5 file size**	5.5 GB	39 GB

cycles to complete the full search.

Experimental Setup

The HARU system, proposed in section "Design of Hardware Accelerated Read Until", was implemented on Xilinx's Kria AI Starter Kit with a Zynq Ultrascale+ XCK26-SFVC784-2LV-C MPSoC. This board contains a processing system with a quad-core ARM Cortex A53 CPU and 4GB of DDR4 memory (specifications on column 'MPSoC' in Table 2). Implementation details of HARU will be discussed in the section "HARU implementation". This HARU implementation is compared to two pure software implementations discussed in section "Pure software implementations". These two software versions are executed on a desktop computer comprising of a 10-core Intel Core-i9 processor and a high-performance computer (server) with a 36-core Intel Xeon processor (specifications are in Table 2). We performed the experiments on two representative datasets detailed in section "Datasets.

HARU implementation

The operating system running on the processing system of the board is a customized embedded Linux image generated using Xilinx's Petalinux 2021.1 tool. To show the bare minimum throughput of the accelerator, our sDTW accelerator is synthesized with a single query processor in the accelerator clocked at 100 MHz. The number of query processors that can fit in the FPGA depends on the available resource on the device; see section "Resource utilization" for resource utilization of the accelerator with a single query processor.

sDTW Hardware Accelerator: The sDTW accelerator was implemented using Verilog Hardware Descriptive Language (HDL). Synthesis was performed using Xilinx's Vivado 2021.1. The control bus interface for the accelerator utilizes the AMBA AXI-Lite protocol. We utilize the AMBA AXI-Stream protocol through the AXI DMA hardware in the FPGA for high-throughput data transfer for the query and reference data.

HARU Driver: Device drivers were implemented for the hardware accelerator and AXI DMA in the C programming language. The accelerator and AXI DMA's drivers memory map the physical address of corresponding devices into the virtual address space for utilization by the user space applications. The shared communication memory buffers between software and FPGA are preserved on the DDR memory, which is allocated during the initialization stage.

Software Processing Layer: The software processing layer that prepares the raw signals and performs the selecting decision was implemented in the C programming language. For benchmarking experiments, the software loads raw signal data in the BLOW5 format [30] from a USB 3 external hard drive connected to the Kria board. Raw signals for a batch of reads are first loaded to the Random Access Memory (RAM) and are pre-processed using multiple threads implemented using POSIX threads. Pre-processing steps include event detection, prefix trimming, and normalization (explained in section "Software Processing Layer"). Then, sDTW is performed on each read in hardware by iteratively calling the HARU driver. Once the mapped positions and the DTW scores are available for the whole batch, the software computes the mapping quality (MAPQ) [31] and executes the selection criteria based on this score [8].

Pure software implementations

RUscripts: Original *RUscripts* written by Loose et al. [8] using Python 2.7 has reached end-of-life support and target ONT's R7 Nanopore chemistry, which is no longer in use. We modified *RUscripts* to work on Python 3.6+ and extended it to support BLOW5 format and ONT's current Nanopore chemistry R9.4. This support for R9.4 chemistry is implemented by integrating the R9.4 pore model and R9.4 event detection parameters [32, 33].

Optimised *RUscripts* in C: As the Python *RUscripts* is not efficient enough for a fair comparison, we implemented a multi-threaded C implementation that follows similar algorithmic steps. This implementation in C is very similar to the software explained above (section "Software Processing Layer") except that sDTW on the CPU is called with multiple threads instead of using the FPGA accelerator. The sDTW computation on the CPU is performed using the optimized sDTW implementation in the *mlpy* library [25].

Datasets

HARU was tested against combinations of software running on the systems mentioned in Table 2 on two datasets. The first dataset is the SARS-CoV-2 genomic reads sequenced on a MinION flowcell and has a total of 1.382 million reads (Table 3), publicly available at [34]. SARS-CoV-2 genome (MN908947.3), which is 29903 bases long, is used as the reference for this experiment. The second dataset is a subset of a NA12878 human genome reference sample containing 500,000 reads sequenced on a PromethION flowcell (Table 3), publicly available at [30]. This dataset is mapped to a reference constructed by extracting the region chr4:39262456-39391375 (128 Kbases long) of the human genome (hg38). This region includes the RFC1 gene, which contains an important pathogenic variant indicative of hereditary cerebellar ataxias disease and selective sequencing has been applied [1] for accurate diagnosis.

Discussion

In our proof-of-concept implementation of HARU, the reference sequence is first loaded onto the FPGA's on-chip memory (block RAM) at the beginning of the execution. During alignment, the PE

chain streams the reference samples from the block RAM to the first PE (Fig. 11). On-chip memory (block RAM) on the Xilinx Kria board used for evaluation is limited to 5.1 Mb, thus limiting the maximum reference sequence size to 295 kilobases. To eliminate this limitation, future work could directly stream the reference together with the query sequence before each sDTW call (the query sequence is already streamed in the current HARU implementation). However, even with HARU (linear time complexity for sDTW), performing sDTW of a query on a giga-base-sized genome like the human genome is impractical (estimated to take 3 seconds for a query). Nevertheless, when processing giga-based sized genomes, HARU is intended to be used in the final refinement step when potential mapping locations (a few reference sequence segments that are small in size) are first found using a heuristic method. Such a heuristic method that can currently map nanopore signals directly to giga-based sized genomes does not exist. However, methods such as Sigmap [18] and UNCALLED [19] are already setting the foundation for scalable direct signal mapping.

Future work can also improve the throughput by implementing multiple parallel sDTW cores for coarse-grain parallelism. Our sDTW processor uses less than 20% of the LUT resources of the FPGA, as mentioned in Section "Resource utilization". Thus, resources are sufficient to implement five parallel processors, increasing the theoretical throughput by five times. A high-end FPGA board with a larger area could support even more processors; for instance, Xilinx's Versal VP2802 FPGA has sufficient resources to theoretically support 140 parallel processors. However, such work also would require eliminating other bottlenecks that would arise. For instance, the 30% of execution time currently spent on the signal pre-processing (Fig. 6) would then become a bottleneck and require acceleration.

Our implementation of HARU loads raw signal from BLOW5 file format because the slow5lib library is lightweight (with minimal dependencies), thus, easily allowing the cross-compilation to target the Kria platform. Running MinKNOW on the Kria platform is theoretically possible but is far from practicality due to being closed source. Even if MinKNOW were open source, potential issues with hundreds of bulky dependencies would make cross-compilation impractical. Potential workarounds could include a server-client approach where MinKNOW runs on a laptop and communicates with the Kria board using ethernet. However, such workarounds are not ideal due to network communication overheads. Also, latency in the public-facing ReadUntil API provided by ONT (in Python programming language) would negate the massive benefit of hardware acceleration.

Our proof-of-concept HARU implementation is currently limited to DNA on R9.4 chemistry. Future work could focus on extending selective RNA sequencing in future or upcoming protein sequencing from ONT. Supporting the most recent R10.4 will be possible when a pore model for R10.4 becomes available.

Related Hardware Acceleration Work

Existing hardware acceleration work targeting the subsequence search problem using the DTW algorithm family is rare. Previous FPGA accelerators such as [35, 36] implement a cDTW accelerator to compute the distance score between a query and a window buffer that stores a subset of the reference sequence. The reference sequence is continuously streamed into the window after each cDTW compute iteration completes, shifting older samples out. A distance score that is below a preset threshold indicates a match between the query and the current reference subsequence in the window buffer. [35] focused on exploiting coarse-grain parallelism by computing multiple cDTW in parallel. [36] introduced a PE-ring structure that computes multiple recurrence equations in parallel where the processing elements (PEs) compute cells that do not share data dependencies. This windowed cDTW approach is suitable for reference sequences of undetermined arbitrary length. Still, it is inefficient (requires $N\times O(M^2)$ for software approaches) for selective sequencing usage where the reference sequence is static with a known length. sDTW, on the other hand, is a data-reusing version of the approach and our work exploits the fine-grain parallelism that computes the whole $O(M)$ dimension in parallel, leaving $O(M+N)$ computational time and $O(M)$ space.

For the hardware acceleration on signal-alignment Read Until, the only previous attempt was a simulated Application Specific Integrated Circuit (ASIC) design that accelerates the sDTW algorithm [37]. The proposed accelerator uses the unprocessed raw signal reads to map directly with the reference, which requires 2000 PEs to perform the matching and has a reference limit of 100KB. The design has extensive resource requirements making it difficult to fit on lower-cost reconfigurable hardware, thus targeting ASIC. Furthermore, as seen in the history of Read Until [8, 9, 10], Read Until requires implementations to adapt quickly as nanopore sequencing technology improves, and the cost of re-manufacturing ASICs would be unsustainable. In contrast, HARU is a complete design with an efficient software processing layer utilizing the sDTW accelerator. Our presented accelerator requires only 250 resource-efficient PEs due to pre-processing reducing the query size needed in the high-throughput computation of sDTW and is capable of executing selective sequencing with low-cost embedded MPSoC platforms with on-chip reconfigurable hardware.

Conclusion

Existing sDTW-based software methods for nanopore selective sequencing are highly computationally intensive, and a large workstation cannot keep up with a portable MinION sequencer. In this paper, we present HARU, a resource-efficient design that enables sDTW-based selective sequencing on a low-cost and portable heterogeneous system comprised of an ARM processor and an FPGA, which is around 85× faster than the original sDTW-based software implementation and around 2.5× faster than a highly optimized software version running on a server with a 36-core Xeon processor for a complete SARS-CoV-2 dataset. The energy-delay product for the server is around 650× higher than HARU executing on an embedded device.

Availability of source code and requirements

HARU

- Project name: HARU
- Description: Source code for the HARU accelerator, including the Verilog HDL core accelerator and user-space device driver
- Project home page: https://github.com/beebdev/HARU
- Operating system(s): Windows 10/11 (building), Custom Embedded Linux image built with PetaLinux 2021.1 (running)
- Programming language: Verilog, C, Python
- Other requirements: Vivado 2021.1, Petalinux 2021.1
- License: MIT

Sigfish-HARU

- Project name: Sigfish-HARU
- Description: Source code that demonstrates the proof-of-concept integration of HARU accelerator for squiggle mapping
- Project home page: https://github.com/beebdev/sigfish-haru
- Operating system(s): Linux (building), embedded Linux built with PetaLinux 2021.1 (running)
- Programming language: C
- Other requirements: Cross-compilation toolchain for AARCH64

- License: MIT

RUScripts-R9

- Project name: RUScripts-R9
- Description: The modified RUscripts to support Python 3.6+, BLOW5 format and ONT's current Nanopore chemistry R9.4
- Project home page: https://github.com/beebdev/RUscripts-R9
- Operating system(s): Platform Independent
- Programming language: Python
- Other requirements: Python 3.6
- License: MIT

Availability of supporting data and materials

Datasets used for the benchmarks are available to be directly downloaded from https://doi.org/10.5281/zenodo.7314838, which we curated from publicly available datasets:

- https://community.artic.network/t/links-to-raw-fast5-fastq-data-for-artic-protocol/17 associated with publication [34]
- https://www.ncbi.nlm.nih.gov/sra/SRX11368475 associated with publication [30]

Declarations

List of abbreviations

API: Application Programming Interface
ASIC: Application-Specific Integrated Circuit
AXI: Advanced eXtensive Interface
cDTW: Classical Dynamic Time Warping
CLB: Configurable Logic Block
CPU: Central Processing Unit
DMA: Direct Memory Access
DTW: Dynamic Time Warping
FPGA: Field-Programmable Gate Arrays
GPU: Graphics Processing Unit
HDL: Hardware Descriptive Language
HPC: High-Performance Computing
LUT: Lookup Table
MAPQ: Mapping Quality
MPSoC: Multi-Processor System-on-Chip
ONT: Oxford Nanopore Technologies
PE: Processing Element
RAM: Random Access Memory
sDTW: Subsequence Dynamic Time Warping
TDP: Thermal Design Power

Consent for publication

Not applicable.

Competing Interests

H.G. has received travel and accommodation expenses to speak at Oxford Nanopore Technologies conferences. The authors declare no other competing interests.

Author's Contributions

H.G., H.S. and S.P. conceived the work. P.S. and H.S. designed and implemented the hardware accelerator. P.S. designed and implemented the device driver. H.G. designed and implemented the optimised multi-threaded signal mapping software (*sigfish*). P.S. drafted the manuscript. H.G. and S.P. revised the manuscript. H.G. and P.S. devised the experiments and benchmarks. P.S. conducted the experiments and benchmarks. All authors read and approved the manuscript.

References

1. Stevanovski I, Chintalaphani SR, Gamaarachchi H, Ferguson JM, Pineda SS, Scriba CK, et al. Comprehensive genetic diagnosis of tandem repeat expansion disorders with programmable targeted nanopore sequencing. Science Advances 2022;8(9):eabm5386. https://www.science.org/doi/abs/10.1126/sciadv.abm5386.
2. Miller DE, Sulovari A, Wang T, Loucks H, Hoekzema K, Munson KM, et al. Targeted long-read sequencing identifies missing disease-causing variation. The American Journal of Human Genetics 2021;108(8):1436–1449.
3. Djirackor L, Halldorsson S, Niehusmann P, Leske H, Capper D, Kuschel LP, et al. Intraoperative DNA methylation classification of brain tumors impacts neurosurgical strategy. Neuro-Oncology Advances 2021;3(1):vdab149.
4. Yamaguchi K, Kasajima R, Takane K, Hatakeyama S, Shimizu E, Yamaguchi R, et al. Application of targeted nanopore sequencing for the screening and determination of structural variants in patients with Lynch syndrome. Journal of Human Genetics 2021;66(11):1053–1060.
5. Wang M, Fu A, Hu B, Tong Y, Liu R, Liu Z, et al. Nanopore targeted sequencing for the accurate and comprehensive detection of SARS-CoV-2 and other respiratory viruses. Small 2020;16(32):2002169.
6. Marquet M, Zöllkau J, Pastuschek J, Viehweger A, Schleußner E, Makarewicz O, et al. Evaluation of microbiome enrichment and host DNA depletion in human vaginal samples using Oxford Nanopore's adaptive sequencing. Scientific reports 2022;12(1):1–10.
7. Martin S, Heavens D, Lan Y, Horsfield S, Clark MD, Leggett RM. Nanopore adaptive sampling: a tool for enrichment of low abundance species in metagenomic samples. Genome Biology 2022;23(1):1–27.
8. Loose M, Malla S, Stout M. Real-time selective sequencing using nanopore technology. Nature methods 2016;13(9):751–754.
9. Edwards HS, Krishnakumar R, Sinha A, Bird SW, Patel KD, Bartsch MS. Real-time selective sequencing with RUBRIC: read until with basecall and reference-informed criteria. Scientific reports 2019;9(1):1–11.
10. Payne A, Holmes N, Clarke T, Munro R, Debebe BJ, Loose M. Readfish enables targeted nanopore sequencing of gigabase-sized genomes. Nature biotechnology 2021;39(4):442–450.
11. Wang Y, Zhao Y, Bollas A, Wang Y, Au KF. Nanopore sequencing technology, bioinformatics and applications. Nature biotechnology 2021;39(11):1348–1365.
12. Li H. Minimap2: pairwise alignment for nucleotide sequences. Bioinformatics 2018;34(18):3094–3100.
13. Jain M, Koren S, Miga KH, Quick J, Rand AC, Sasani TA, et al. Nanopore sequencing and assembly of a human genome with ultra-long reads. Nature biotechnology 2018;36(4):338–345.
14. Deamer D, Akeson M, Branton D. Three decades of nanopore sequencing. Nature biotechnology 2016;34(5):518–524.
15. Petersen LM, Martin IW, Moschetti WE, Kershaw CM, Tsongalis GJ. Third-generation sequencing in the clinical laboratory: ex-

ploring the advantages and challenges of nanopore sequencing. Journal of clinical microbiology 2019;58(1):e01315–19.
16. Logsdon GA, Vollger MR, Eichler EE. Long-read human genome sequencing and its applications. Nature Reviews Genetics 2020;21(10):597–614.
17. Wick RR, Judd LM, Holt KE. Performance of neural network basecalling tools for Oxford Nanopore sequencing. Genome biology 2019;20(1):1–10.
18. Zhang H, Li H, Jain C, Cheng H, Au KF, Li H, et al. Real-time mapping of nanopore raw signals. Bioinformatics 2021;37(Supplement_1):i477–i483.
19. Kovaka S, Fan Y, Ni B, Timp W, Schatz MC. Targeted nanopore sequencing by real-time mapping of raw electrical signal with UNCALLED. Nature biotechnology 2021;39(4):431–441.
20. Ulrich JU, Lutfi A, Rutzen K, Renard BY. ReadBouncer: Precise and Scalable Adaptive Sampling for Nanopore Sequencing. bioRxiv 2022;.
21. Kruskal JB. An overview of sequence comparison: Time warps, string edits, and macromolecules. SIAM review 1983;25(2):201–237.
22. Müller M. Dynamic time warping. Information retrieval for music and motion 2007;p. 69–84.
23. Juang BH. On the hidden Markov model and dynamic time warping for speech recognition—A unified view. AT&T Bell Laboratories Technical Journal 1984;63(7):1213–1243.
24. Tuzcu V, Nas S. Dynamic time warping as a novel tool in pattern recognition of ECG changes in heart rhythm disturbances. In: 2005 IEEE international conference on systems, man and cybernetics, vol. 1 IEEE; 2005. p. 182–186.
25. Albanese D, Visintainer R, Merler S, Riccadonna S, Jurman G, Furlanello C. mlpy: Machine learning python. arXiv preprint arXiv:12026548 2012;.
26. Keogh E, Wei L, Xi X, Lee SH, Vlachos M. LB_Keogh supports exact indexing of shapes under rotation invariance with arbitrary representations and distance measures. In: Proceedings of the 32nd international conference on Very large data bases Citeseer; 2006. p. 882–893.
27. Lemire D. Faster retrieval with a two-pass dynamic-time-warping lower bound. Pattern recognition 2009;42(9):2169–2180.
28. Sakoe H, Chiba S. Dynamic programming algorithm optimization for spoken word recognition. IEEE transactions on acoustics, speech, and signal processing 1978;26(1):43–49.
29. Itakura F. Line spectrum representation of linear predictor coefficients of speech signals. The Journal of the Acoustical Society of America 1975;57(S1):S35–S35.
30. Gamaarachchi H, Samarakoon H, Jenner SP, Ferguson JM, Amos TG, Hammond JM, et al. Fast nanopore sequencing data analysis with SLOW5. Nature biotechnology 2022;p. 1–4.
31. Li H, Handsaker B, Wysoker A, Fennell T, Ruan J, Homer N, et al. The sequence alignment/map format and SAMtools. Bioinformatics 2009;25(16):2078–2079.
32. Gamaarachchi H, Lam CW, Jayatilaka G, Samarakoon H, Simpson JT, Smith MA, et al. GPU accelerated adaptive banded event alignment for rapid comparative nanopore signal analysis. BMC bioinformatics 2020;21(1):1–13.
33. Simpson JT, Workman RE, Zuzarte P, David M, Dursi L, Timp W. Detecting DNA cytosine methylation using nanopore sequencing. Nature methods 2017;14(4):407–410.
34. Rodriguez-Morales AJ, Gallego V, Escalera-Antezana JP, Méndez CA, Zambrano LI, Franco-Paredes C, et al. COVID-19 in Latin America: The implications of the first confirmed case in Brazil. Travel medicine and infectious disease 2020;35:101613.
35. Sart D, Mueen A, Najjar W, Keogh E, Niennattrakul V. Accelerating dynamic time warping subsequence search with GPUs and FPGAs. In: 2010 IEEE International Conference on Data Mining IEEE; 2010. p. 1001–1006.
36. Wang Z, Huang S, Wang L, Li H, Wang Y, Yang H. Accelerating subsequence similarity search based on dynamic time warping distance with FPGA. In: Proceedings of the ACM/SIGDA international symposium on Field programmable gate arrays; 2013. p. 53–62.
37. Dunn T, Sadasivan H, Wadden J, Goliya K, Chen KY, Blaauw D, et al. SquiggleFilter: An Accelerator for Portable Virus Detection. In: MICRO-54: 54th Annual IEEE/ACM International Symposium on Microarchitecture; 2021. p. 535–549.
