# Supplementary material for: Efficient real-time selective genome sequencing on resource-constrained devices
Source: Gigascience. 2023 Jul 3;12:giad046. doi: 10.1093/gigascience/giad046 (PMC10316692; doi:10.1093/gigascience/giad046)
Supplement: giad046_GIGA-D-22-00317_Revision_1 [file giad046_giga-d-22-00317_revision_1.pdf]

GigaScience

Efficient Real-Time Selective Genome Sequencing on Resource-Constrained Devices

--Manuscript Draft--

**Manuscript Number:**	GIGA-D-22-00317R1
**Full Title:**	Efficient Real-Time Selective Genome Sequencing on Resource-Constrained Devices
**Article Type:**	Technical Note
**Funding Information:**	Australian Research Council (DE230100178) — Dr Hasindu Gamaarachchi
**Abstract:**	Background: Third-generation nanopore sequencers offer selective sequencing or `Read Until' that allows genomic reads to be analyzed in real-time and abandoned halfway if not belonging to a genomic region of `interest'. This selective sequencing opens the door to important applications such as rapid and low-cost genetic tests. The latency in analyzing should be as low as possible for selective sequencing to be effective so that unnecessary reads can be rejected as early as possible. However, existing methods that employ subsequence Dynamic Time Warping (sDTW) algorithm for this problem are too computationally intensive that a massive workstation with dozens of CPU cores still struggles to keep up with the data rate of a mobile phone-sized MinION sequencer.<br>Results: In this paper, we present Hardware Accelerated Read Until (HARU), a resource-efficient hardware-software co-design-based method that exploits a low-cost and portable heterogeneous Multiprocessor System-on-Chip (MPSoC) platform with on-chip Field-Programmable Gate Arrays (FPGA) to accelerate the sDTW-based Read Until algorithm. Experimental results show that HARU on a Xilinx FPGA embedded with a 4-core ARM processor is around 2.5X faster than a highly optimized multi-threaded software version (around 85X faster than the existing unoptimized multi-threaded software) running on a sophisticated server with 36-core Intel Xeon processor for a SARS-CoV-2 dataset. The energy consumption of HARU is two orders of magnitudes lower than the same application executing on the 36-core server.<br>Conclusions: HARU demonstrates that nanopore selective sequencing is possible on resource-constrained devices through rigorous hardware/software optimizations. The source code for HARU sDTW module is available as open-source at https://github.com/beebdev/HARU, and an example application that utilizes HARU is at https://github.com/beebdev/sigfish-haru.
**Corresponding Author:**	Hasindu Gamaarachchi<br>Garvan Institute of Medical Research<br>Darlinghurst, AU-NSW AUSTRALIA
**Corresponding Author Secondary Information:**	
**Corresponding Author's Institution:**	Garvan Institute of Medical Research
**Corresponding Author's Secondary Institution:**	
**First Author:**	Po Jui Shih
**First Author Secondary Information:**	
**Order of Authors:**	Po Jui Shih<br>Hassaan Saadat<br>Sri Parameswaran<br>Hasindu Gamaarachchi
**Order of Authors Secondary Information:**	
**Response to Reviewers:**	The nicely formatted PDF containing the reviewer's response is attached as a supplementary material.

Powered by Editorial Manager® and ProduXion Manager® from Aries Systems Corporation

Editor's comments

- Overall, the reviewers are supportive of publication. One major concern mentioned by several reviewers is that the evaluation and comparisons to similar approaches need to be improved, also to highlight differences and improvements compared to existing work.

Initially, we did not provide comparisons with other methods because none of them was designed to be executed on a portable, low-cost and resource-constrained device like the one HARU runs on, which has only 4-ARM cores, 4GB RAM, and consumes less than 3W of power. Nonetheless, to address the reviewers comments in this revised manuscript, we executed those other methods (DeepSelectNet, Minimap2+Guppy, etc) on high-end devices and compared them to HARU executed on a low-end embedded system. Although the difference in execution platform makes the comparison biased in favour of other methods, our results show that HARU still has better performance.

- Reviewer #2 mentions that "the source code for both implementations of the optimized RUscripts in C and Python is not open nor available". Please note it is a requirement for publication in GigaScience that all supporting code is available under an OSI-approved open licence.

The links to these source codes were already provided as open-source under “Availability of source code and requirements” section. Now we have also added the links to all repositories as key-points in the manuscript so those links may not be accidentally overlooked by a reader.

- On a minor note, please also structure your abstract ("Background", "Findings", "Conclusions").

We have structured the abstract as per your suggestion.

Reviewer #1:

This submission is a solid application work to accelerate low-latency, signal-level genome alignment on FPGAs. It provides an end-to-end design for portable sequencers to analyze input genome and discard unnecessary reads in real-time. By providing an open-source RTL design with an integration example, the work brings 2.5× performance with impressive energy efficiency to the community. I would expect this work could facilitate future research.

This paper is well-written, with an extensive explanation of the background. I found it entertaining to read, even with little background knowledge of signal-level processing of genomes. The research goal of low-cost targets and low-latency processing are well motivated. The experimental results are solid and extensive, with an optimized baseline for comparison and end-to-end performance analysis, including overhead time. The authors combine knowledge from different fields and co-optimize the design, for example, the data scaling with acceptable accuracy reduction.

We thank the reviewer for these encouraging comments.

Q1. Although well-written, my main concern about this work is that it seems incremental and needs to exhibit more research innovation. The authors successfully combine multiple optimizations, but most optimization approaches are well-known. For example, the 2D dynamic programming (DP) algorithm without backtrace requirement is well-studied for not only FPGA accelerations but also from the space reduction perspective. Pipelining and data reusing are both standard practices in the FPGA community. I would appreciate it if the authors could highlight the improvement and difference from the regular 2D DP problem and elaborate more on the novelty of their work at the system integration level.

We appreciate the reviewer's feedback and agree that the initial manuscript did not emphasize the novelty of the system-level integration work sufficiently. Our system-

Powered by Editorial Manager® and ProduXion Manager® from Aries Systems Corporation

level integration of our end-to-end overarching architecture involves several novel components, such as optimized squiggle preparation for normalization using multithreading, the custom driver for high-throughput transferring via the AMBA AXI4-Stream, and a portable and efficient hardware accelerator. We can also use Kria's xmutil utility tool to swap bitstreams without needing to reboot the system, which makes it easy to adapt to changes or customize towards applications if needed. The hardware control interface uses AXI-Lite, while data streaming (query and reference) uses AXI stream, which enables high throughput on low-cost MPSoC platforms.
We have updated the introduction of the manuscript to highlight the novelty of our system-level integration (page 2).

Q1.2. Another concern is that this work needs to discuss its related work sufficiently. Although there are a few paragraphs on the acceleration of signal alignment, only a few base-alignment approaches are referred to. Although different in the format and semantics of the input data, the algorithm is similar, and I believe the authors could reuse many of the existing accelerator designs. Besides, the discussion on Minimap2 on page 2 is not accurate, as there is at least one published FPGA acceleration (Hardware Acceleration of Long Read Pairwise Overlapping in Genome Sequencing: A Race Between FPGA and GPU), along with GPU implementations. This inaccuracy also undermines the motivation of processing at the signal level. I would appreciate it if the authors further discussed the reason for using a signal-based approach.

Thank you for your feedback on related work. Currently, when performing base-level alignment-based selective sequencing, the bottleneck is basecalling and not the alignment (Minimap2). CPU-based Minimap2 is still very fast and takes only 2-5% of the time in a base-level alignment-based selective sequencing workflow. 95-98% of the time is spent on basecalling, despite using GPU (fast basecalling model). Therefore, those accelerations on Minimap2 are less relevant unless the basecalling in significantly accelerated. We have clarified on page 2 of our report that Minimap2 is not the bottleneck and that the basecalling process is the cause of the bottleneck.

We have also discussed recent acceleration on Minimap2 on page 3.

In fact, the observation that alignment in the base-space is very fast compared to basecalling is what makes signal alignment a potentially better method. We have clarified this on page 3.

Q1.3. There are a few minor comments I would appreciate if the authors could address before publication:

(1.3.1) It seems that as M is implemented as the pipeline depth, it is set to a constant. Does M changes as the data changes? Or does the detection accuracy change if M does not change with the data?

We conducted experiments investigating the impact of query length (denoted by M) on mapping accuracy and found that accuracy increased with longer query lengths. However, trimming the prefix sequence (which belongs to the adaptor and barcode, if present) proved more crucial for maintaining high accuracy. This prefix is pre-determinable and is more desired than having a varying query length. A query length of 250 was chosen to balance speed and accuracy. If for other reasons a different query length is needed our hardware can be updated to accommodate varying query lengths, allowing users to dynamically choose the appropriate bitstream using the Kria platform's xmutil tool. Longer query lengths, though, may require increased squiggle collection wait time and risk pore damage from clogging. We have discussed this in Supplementary Note 5: “Selection of query length” associated with the updated manuscript.

1.3.2 Does the data scaling method work for all data? How about using dynamic scaling instead of a set scale?

The data scaling method we used involves converting the 13-bit MinION integer data and 11-bit PromethION integer data to real numbers using picoampere conversion and z-score normalization. These real numbers are then represented and processed in fixed-point representation with a scaling factor of 32 (5 fractional bits) on the HARU

Powered by Editorial Manager® and ProduXion Manager® from Aries Systems Corporation

hardware to achieve resource efficiency.

Does the data scaling method work for all data?
We found that the normalized data typically follow a Gaussian distribution, and after normalization, only a very small fraction of samples (0.0027%) have a magnitude greater than 3. We accumulated such values over 512 samples, so the probability of the magnitude exceeding 1024 is negligible. Based on this, we determined that using 16 bits (11 integer bits and 5 fractional bits) is sufficient to represent all types of data without significant loss of precision.

We also performed experiments to empirically validate this method for multiple target genomes, including SARS-CoV-2, Ebola, Ecoli, and Lambda datasets publicly available. Supplementary Note 1, associated with the revised mansuscript, now discusses these experiments and the mapping accuracy percentage difference between sigfish using 32-bit floating points and sigfish-HARU which uses 16-bit fixed-point with scaling method. The accuracy of each version of sigfish measured against the results obtained from Minimap2 (if the mapping coordinate is within 200 bases proximity they are considered correct) only differed by 0-0.05% for all the genomes tested.

How about using dynamic scaling instead of a set scale?
Dynamic scaling can be useful when the dynamic range of the input data is not known in advance, as it allows for the scaling factor to be adjusted on-the-fly to accommodate varying input magnitudes. However, in the case of our sDTW algorithm, the input data comes from a well-defined and consistent range, as explained earlier, making a static scaling factor a more appropriate and efficient choice for hardware implementation. Additionally, fixed-point arithmetic with a set scaling factor can often be faster and more power-efficient than using dynamic scaling or floating-point arithmetic, which requires more complex hardware and consumes more power as discussed in Supplementary Note 6.

1.3.3 In Figure 7, "25" should be "256".

We have updated the manuscript.

Nevertheless, this work is publishable, and the audiences will benefit from its results.

We again thank the reviewer for the positive and constructive feedback that allowed us to improve the quality of the manuscript.

Reviewer #2:
The authors observe that existing Read Until approaches that employ subsequence Dynamic Time Warping (sDTW) algorithm are computationally intensive such that a capable workstation with dozens of CPU cores struggles to keep up with the data rate of a mobile phone-sized MinION sequencer.

The authors present Hardware Accelerated Read Until (HARU), a resource-efficient hardware-software co-design-based method that exploits a low-cost and portable heterogeneous Multiprocessor System-on-Chip (MPSoC) platform with on-chip Field-Programmable Gate Arrays (FPGA) to accelerate the sDTW-based Read Until algorithm. Experimental results show that HARU on a Xilinx FPGA embedded with a 4-core ARM processor is around 2.5× faster than a highly optimized multi-threaded software version (around 85× faster than the existing unoptimized multi-threaded software) running on a sophisticated server with 36-core Intel Xeon processor for a SARS-CoV-2 dataset.

I like this work. The paper appears to provide significant research contributions. It targets tackling an important problem in the genome sequencing pipeline. The following points need to be addressed.

We thank the reviewers for these positive comments.

Powered by Editorial Manager® and ProduXion Manager® from Aries Systems Corporation

Q2.1 The authors make several optimization techniques that I appreciate and agree with. However, the effect of each of these techniques on the execution time, FPGA resource allocation, and more importantly the accuracy of Read Until decisions are not provided.

We thank the reviewer for the suggestion to include more details about the effect of our optimization techniques on execution time, FPGA resource allocation, and accuracy of Read Until decisions.

Accuracy:
The pipelining optimization does not change how sDTW is computed and thus does not affect the accuracy. For operations to be pipelined, each operation can only start after its dependent data are ready, and all computations are done exactly the same as non-pipelined computations. Thus, accuracy is not affected by pipelining. We have now briefly stated this in the subsection "Operation pipelining" (page 8) of the updated manuscript.
The cost matrix memory optimization is applicable in the selective sequencing application because we only need either the start or end of the mapping of squiggles in the reference, which means backtracking is not needed as long as we keep track of the minimum value in the last row of the cost matrix. The cost matrix memory optimization still returns the same end position of the mapping as is without the optimization. We have briefly stated this in the subsection "Cost matrix memory optimization" (page 8) of the updated manuscript.
However, we also acknowledge that using fixed-point representation may result in some loss of precision, and we already explored this in detail in the subsection "Fixed-point data representation" of the original manuscript. In addition, in the supplementary Note 1 of the updated manuscript, we have included experimental results that the accuracy of the fixed point representation is nearly identical to using floating point (see the response in reviewer#1's question)
FPGA resource allocation and Execution time

Different optimizations can interact with one another, potentially resulting in varying execution times and resource allocations when different combinations are applied. As such accurately measuring the effect due to individual optimizations poses a significant difficulty. Therefore, we believe that, ultimately, it's the final execution time and the FPGA resource allocated with all optimizations in place that matter most which we had reported and discussed extensively in the original manuscript. Nonetheless, we have further added Supplementary Note 6 to highlight the impact of different optimizations as best as we can.

Q2.2 I would like to understand more about the effect of choosing 250 events to decide on Read Until. Is sampling more than 250 events needed? The length of read is also an important factor as the length of the ONT reads varies from tens to a few million bases.

We have now discussed this in Supplementary Note 5: "Selection of query length" associated with the updated manuscript. The same question was also raised by reviewer 1. The reviewer is requested to kindly refer to the response to Q1.3.1 - query made by reviewer 1 on M being implemented as the pipeline depth.

Q2.3 The source code for both implementations of the optimized RUscripts in C and Python is not open nor available. I would highly encourage the authors to make them available on github.

We apologize for any confusion caused. The links to these source codes were already provided as open-source under "Availability of source code and requirements" in the original manuscript. The Optimised RUScripts implementation in C is in the same repository as sigfish-haru which can be specified as an option to make:

```
# Building sigfish without hardware acceleration
make PROCESSOR=aarch64
# Building sigfish with hardware acceleration
make fpga=1 PROCESSOR=aarch64
```

Powered by Editorial Manager® and ProduXion Manager® from Aries Systems Corporation

To avoid such confusion, we added the following as a key point under the manuscript (page 2).
Q2.4 The evaluation of how HARU scales with a different number of threads is not provided. Both the front software interface for HARU and the RUscripts are multithreaded, but the number of CPU threads that makes RUscripts faster than HARU needs to be examined.

The experiments in our paper were conducted with RUScripts executed with all threads available on the system, which included 36 cores when executing on the HPC. Although RUScripts was originally written in Python and was not optimized for performance or multithreading efficiency, we re-implemented it in C and optimized it for performance and multithreaded efficiency to provide a fair comparison with HARU. The runtime for the optimized multithreaded implementation of RUScripts was also provided in the paper. As stated in the paper, our HARU system on the edge computing board (costing around $300) is ~85.8× faster than the original RUscripts running with 36 cores on a server and still ~2.49× faster than the 36-core server (costing around $30,000). In the following extract from the original manuscript (now page 4 of the updated manuscript), the fact that all threads were used was stated:

Q2.5 I wonder if the authors also evaluate the benefits of using HARU for base-domain Read Until. The basecalling step contributes significantly to the read accuracy and performing operations in the signal space is still challenging. This is true for most of the Read Until applications. Showing the pros and cons through evaluation of discussion for using HARU for signal domain Read Until versus base domain Read Until can be very useful.

We agree with the reviewer's suggestion that evaluating the benefits of using HARU for base-domain Read Until would be useful. We acknowledge that basecalling is a challenging step in nanopore sequencing and that performing operations in the signal space is still a developing field. However, we believe that signal processing holds great potential for nanopore adaptive sampling on-chip. While piggybacking on existing sequence alignment techniques is easier, signal processing advancements in other fields such as image, video, and audio signal processing have led to the development of miniaturized devices with comparable data rates to nanopore sensors. We have now discussed the pros and cons of each approach under the discussion on page 9 and 10 of the updated manuscript.

Q2.6 For Figure 4, the throughput of the target sequencing machine needs to be added to have a clear comparison with the current need for acceleration.

The relationship between the throughput of the target sequencing machine and the throughput required by the accelerator for effective selective sequencing is very complex. The effectiveness of selective sequencing is determined by whether the reject or sequence decision can be made before the nanopore channel in the sequencer finishes sequencing the current squiggle. The average length of squiggles in nanopore sequencing applications may vary across different targets and can be affected by sample read length distribution. There are also other factors involved in the effectiveness of selective sequencing, including the proportion of on-target and off-target reads in the sample, pore-clogging when a rejection signal is passed back when the nanopore channel has sequenced a majority of the squiggle, software complexities and limitations introduced by MinKNOW (0.4s is the minimum wait time before any data is available). Thus, it is unrealistic to provide any sequencer throughput quantization and discuss its relation with selective sequencing processing throughput to determine Read Until effectiveness.

Q2.7 The authors mention that "Our implementation of HARU loads raw signal from BLOW5 file format because the slow5lib library is lightweight". As the default file format provided by ONT devices is FAST5, I wonder if the execution for format conversion is included in the total execution time.

To clarify, by "lightweight," we meant avoiding the use of bulky libraries. The conversion time is not included because direct loading from RAM through MinKNOW API would eliminate such conversion. However, as the MinKNOW is closed-source and only available in limited settings, we opted to use BLOW5 for this proof-of-concept

Powered by Editorial Manager® and ProduXion Manager® from Aries Systems Corporation

implementation. It's important to note that BLOW5 is also lightweight in terms of runtime, as demonstrated in https://www.nature.com/articles/s41587-021-01147-4. However, the default file format is controlled by the company, which presents a separate issue.

Q2.8 The reference genome needs to be first loaded to FPGA's block RAM. What if the reference genome doesn't fit into the block RAM? What is the reference genome size that is used in practice for Read Until applications?

In our proof-of-concept implementation, we used block RAMs to load the reference genome, but this is not the only approach. A more flexible way to handle reference genomes is to stream them together with the query signal during runtime, allowing for arbitrarily long references. We have implemented this approach in a separate branch of our code (https://github.com/beebdev/HARU/tree/dynamic-reference), and it allows the accelerator to compute the mappings on the fly while the reference is being streamed into the accelerator.
While the reference streaming branch doesn’t limit the reference size, in practice, sDTW-based methods still will not scale for giga-base references. However, we plan to integrate methods such as sigmap and UNCALLED to first perform fast mapping using seeds, followed by running sDTW on potential matches to refine the results. The sDTW mapping operation will involve a much smaller mapping reference size than the full target reference. Currently, these index/seeding-based methods have limitations and require optimizations before they can be fully integrated into our system. We have briefly discussed this under the discussion on page 10 of the updated manuscript.
Q2.9 I understand that the use of low-cost MPSoC is to run the software interface and the hardware accelerator on the same portable device. However, is it possible to use high-end FPGA devices with HBM memories? Is it needed to cope with the throughput of more capable sequencing machines such as PromethION?

While our current implementation targets low-cost MPSoCs, scaling up the hardware implementation to parallelize more PE chain accelerators to handle higher throughputs from more capable sequencing machines such as PromethION is certainly a possibility. Our current design of the accelerator only supports an AXI-stream and AXI-Lite interface, which means that platforms with sufficient resources can parallelize multiple accelerators using multi-channel AXI DMAs. However, efficiently streaming data to a large number of parallel accelerators to achieve the necessary throughput will require faster data buses such as PCIe. Although we have not explored the use of HBM memories or higher-end FPGAs at this point, we appreciate your suggestion for future work and have discussed this possibility under the discussion on page 10 of the updated manuscript.
Q2.10 The command lines for evaluating the existing tools are missing.

We thank the reviewer for reminding this which was overlooked by us. We have added this under Supplementary Note 7.

Q2.11 A few typos:
* page 3, on the left down side, range of i and j have typo.
* On Algorithm 1, first for loop has typo i and j

Thanks. We updated the manuscript and the algorithm.

Reviewer #3:
This paper proposes a hardware-assisted subsequence Dynamic Time Warping solver for selective genome sequencing from raw signal traces from hand-held nanopore sequencers. To improve the efficiency of ReadUntil framework, the proposed framework introduces a systolic array DTW accelerator deployed on an FPGA based SoC, which is integrated into and driven from a host and driver software. Avoiding implementing the full cost matrix allows their design to scale. Improved sDTW processing has achieved large speedup and energy efficiency over a server system with Xeon CPUs.

+ The authors have performed a system-wide study with a full-stack proof-of-concept.
+ Compact PE design that allows it to be implemented on a small FPGA or an edge

device.

In this paper, the authors propose a hardware software co-design for selective genome sequencing using sDTW deployed on FPGA. This paper is well written and easy to read. Notably, one of the strengths of this paper is that they demonstrated the full stack implementation of the proposed FPGA solution, and the hardware and software stacks are open-sourced. I guess non-trivial implementation and verification efforts have been made, and I think this can be one of the reasons for this paper to be accepted.

We thank the reviewer for this positive feedback.

Q3.1 Incremental work to SquiggleFilter [37]. The architecture itself is a deployment of a well-known systolic array architecture for dynamic programming including DTW and Smith-Waterman.

The main focus of HARU is the end-to-end integrated system architecture and it is not limited to the sDTW core. We agree that our sDTW core is built upon well known optimizations in hardware (so does the SquiggleFilter) and the primary innovation lies in the overarching system architecture as appreciated by reviewer 1 and 2.

The sDTW core in HARU started as an undergraduate honours thesis project in 2020 and the design was complete by mid 2021, during which the SquiggleFilter publication did not exist. The designs happened to be similar despite being developed independently, because well-known optimizations have been used in both designs. However, as our sDTW core itself was not a complete system and we realised that there is limited utility of such a hypothetical design, we pursued to develop an end-to-end system closer to integration with a sequencer. In fact, the overall system integration turned out to be a more challenging problem than designing the sDTW core itself. We re-iterated our system for overall performance and robustness, leading to the architecture we presented in our paper. While our architecture is based on well-known systolic array designs for dynamic programming, its novelty and practical usefulness comes from its application-oriented end-to-end system-level design and integration with the broader bioinformatics ecosystem.

As suggested by reviewer 1, we have elaborated on the system design more deeply in the introduction of the updated manuscript.

Q3.2 The design is only compared with a single type of target genome (SARS-CoV-2), and the sensitivity to the sample property is not explored deeply.

The computational method of using event detection followed by sDTW for alignment was originally shown in the RUScripts paper by Loose et al, which has already demonstrated its effectiveness through a series of experiments. HARU is built on top of this Loose et al's work, as acknowledged in the original manuscript. Thus, we believe that repeating multiple such experiments is redundant.

The only optimization in HARU that can cause a deviation from the original sDTW algorithm used in RUscripts is the scaling optimization, all other optimizations guarantee the same answer as sDTW used in Loose et al's work (see the response to Q2.1). To evaluate the impact of this scaling optimization, we have now performed more experiments using different samples such as Ebola, Ecoli, and yeast in the supplementary note. We found out that the accuracy mapping location reported by HARU differs from the accuracy from the original sDTW by only 0-0.05% (see Supplementary Note 1).
In terms of "the sensitivity to the sample property", please refer to the Supplementary Not), to see how the sDTW scores can be used to discriminate between two samples.

Q3.3 Comparisons with the state-of-the-art are not provided.

We have added comparisons to Supplementary Note 4 and a brief summary to section Results (page 6) and Methods (Page 9) of the revised manuscript. Please also refer to

the response for the related Q3.9 for comparisons between HARU and SquiggleFilter, DeepSelectNet, UNCALLED, Guppy + Minimap2.

____

Q3.4 The motivation for this paper lies in the fact that direct read mapping (or adaptive sampling) using Read Until cannot keep up with the sequencing throughput without a full-fledged GPU. While direct mapping using the raw current signals in fast5 admittedly has heavy computation loads, it is not clear to me why direct mapping from squiggles is a good option in the first place. MinION's fast basecalling can be done with an edge GPU or mobile devices (e.g., Mk1C can do fast realtime basecalling using its embedded GPU, and Mk1D is announced to work with iPad Pro's M1 processor and neural engines), and aligning basecalled reads looks much efficient as it needs to deal with less data (fastq is a lot smaller (1/5 ~ 1/10 in size), compared to fast5). While they try to "revitalize the direct signal approach," the motivation and the goal of this work, especially why it should start from the raw current signals, do not seem to be clear. Also, the accuracy is only compared with
RUscripts with sDTW but not with a pipeline with basecalling that many MinION practices follow.

We acknowledge that basecalling from squiggles is a popular and effective approach for many MinION applications, and we did not intend to suggest that it should be replaced. Rather, our goal was to investigate the feasibility and potential benefits of direct signal mapping, which has been previously explored and is an ongoing research area. We believe that direct mapping from squiggles can provide a more complete and accurate representation of the underlying signal, which may be particularly useful for applications that require higher sensitivity or resolution than current basecalling methods can achieve. This is more of a philosophical discussion and we have added a section called “Signal-level vs base-level selective sequencing” on page 9 of the discussion of the updated manuscript.

Reading raw-signals has no such heavy computational bottlenecks if efficient formats such as BLOW5 [https://www.nature.com/articles/s41587-021-01147-4] are used, the problem is in FAST5 rather than with the raw signal. If the raw signal is directly loading from RAM through MinKNOW API, that would anyway eliminate such loads. However, as the MinKNOW is closed-source and only available in limited settings, we opted to use BLOW5 for this proof-of-concept implementation.

Mk1C can do some limited fast realtime basecalling, however, the best-case throughput mentioned on the manufacture’s website cannot be not achieved in practice when we tested. When using Mk1C for selective sequencing, however, enrichment observed is very limited. This is why we currently use Mk1B connected to a workstation with a 3090 GPU for our other in-house selective sequencing experiments that perform redfish. While the Mk1D is announced, it doesn’t yet exist and thus we cannot reach any conclusions.

Regarding the comparison with existing methods, we agree that it would be useful to evaluate the performance of our approach against a pipeline that includes basecalling which we have addressed in the response to Q3.9.

Q3.5 Fundamentally, the sDTW-based approach lacks the seeding step which is critical in the general sequence alignment to reduce search space in the reference genome, so every time all possible positions in the reference need to be searched, and the applicability of this approach looks limited.

We agree that the seeding step is a common technique used in the sequence alignment to reduce the search space in the reference genome, and it can be especially helpful when dealing with long-read sequences. As mentioned in the discussion of our original manuscript, other tools such as sigmap and UNCALLED have already implemented a seeding step and indexing and these tools are continually evolving. Our work with HARU is intended to complement these tools by being used as an accurate refinement step after approximate search methods such as chaining is performed. In fact, general sequence alignment tools such as Minimap2 perform a variant of Smith-Waterman called Suzuki's formulation on potential mapping locations

found by chaining.

Q3.6 SquiggleFilter [37] has introduced a very similar approach for raw signal (squiggle) filtering, using a wide sDTW systolic array on ASIC (their work has also verified with FPGA), in order to quickly find viral genomes. The main difference between SquiggleFilter and this work lies in the input granularity: SquiggleFilter uses 2000 sample points while this work uses 250 events, where each sample point or event needs one PE. I think the authors' approach of using a small number of systolic PEs makes sense to reduce resource amount, however, similar systolic array approaches using the partial diagonal PE band for dynamic programming algorithms such as sDTW and Smith Waterman have been well-known from decades ago [ref1, ref2], and I personally did not find their hardware design has much new insights or novelty compared to the prior works. I believe integrating them into one system with a host software for sure requires non-trivial engineering work, but as an academic paper I think this work is a bit too incremental.

In addition to our response for Q3.1 about the squiggleFilter and novelty, we also like to remind that this article is submitted as a technical note rather than a research article.

Regarding the SquiggleFilter being verified on FPGA, it seems that the authors of SquiggleFilter, despite stating the need for 2000 PEs for 2000 sample points, used only 2 PEs to synthesise for the FPGA implementation results (and only synthesised on AWS instance) while using 100 PEs for behavioural simulation (functional verification). Furthermore, the source code they provided does not contain a synthesizable top-level module that collectively connects all submodules. We opened a GitHub issue on these topics and have received confirmation that the synthesis verification for FPGA was done individually for each submodule (as they do not have the full system and only envision it) and the number of PEs used for synthesis was indeed done on a smaller number that is not 2000 (see https://github.com/TimD1/SquiggleFilter/issues/1). In addition, SquiggleFilter is an ASIC design that is expensive to manufacture and integrate with a full SoC and is vulnerable to selective sequencing requirement changes, while the effort needed to update HARU is much easier. As mentioned in the manuscript, HARU is a low-cost and highly efficient end-to-end Read Until solution that runs on off-the-shelf low-cost FPGA SoCs, which we believe will highly benefit the ongoing improvements in signal-level alignment research in genomic sequencing. Overall, SquiggleFilter does not provide a fully integrated working system (as re-stated by the author in the issue linked above) which is the main difference between HARU and SquiggleFilter. Please refer to the response for Q3.9 for comparison with SquiggleFilter.

Q3.7 Recent work, SquiggleNet [ref3], also proposes a similar pre-filtering approach of raw squiggles using a machine learning model. This model is very lightweight and shown to have good accuracy. It also needs a GPU for fast filtering, but because it is based on the well-known ResNet model, it can be easily accelerated by an accelerator such as an edge TPU. I wonder how the sDTW-based approach compares with such ML-based ones.

While neural network-based methods have some advantages, they also have their own limitations. One significant issue is that they require training for every new dataset, which can be time-consuming and computationally expensive. Moreover, they may not be suitable for applications that require per-position mapping or depletion/enrichment analysis, as they typically require both positive and negative training data from both samples.
Regarding the use of edge TPUs to accelerate SquiggleNet, please note that typical edge TPUs are designed to support int8 types for energy- and power efficiency (e.g., https://cloud.google.com/edge-tpu), while SquiggleNet reports experimentation and implementation on workstation GPUs and does not mention the use of int8. While it is possible to convert the model to int8, it will require additional research and optimization effort, and it is unclear how it would affect the accuracy and efficiency of the model. Thus, using edge TPUs for NN-based selective sequencing is yet a hypothesis.
As for the comparison with neural network-based methods, see the response for Q3.9 that compares with state-of-the-art methods, where we compare HARU with

Powered by Editorial Manager® and ProduXion Manager® from Aries Systems Corporation

	DeepSelectNet, which is a work recently published that optimizes SquiggleNet. Comparing throughput results, HARU exceeds the throughput of DeepSelectNet and the accuracy is slightly better, despite the fact that DeepSelectNet was ran on a HPC system with a high-end Tesla V100 GPU, whereas the HARU runs on a low-power, resource constrained device.<br><br>Q3.8 The evaluation can be improved to have more depth. They only compared a single type of viral genome (SARS-CoV-2) and a narrow region of a human genome. The sensitivity or robustness to the property of input genome, such as repetitions, variants, mutations, and read length, and to the diversity of the mixes of the genome being sequenced (e.g. respiratory metagenome has about 1000:1 human to viral genome ratio) are not explored.<br><br>Please see the response to Q3.2 on experiments using different input genomes. Also, see Q3.12 for the effect of mutations. For experiments on the effect of the query length, please refer to the response to Q1.3 - query made by reviewer 1 on M being implemented as the pipeline depth and also Supplementary Note 5.<br><br>Q3.9 The comparisons have been made based on the RUscripts (and sDTW), but it might be a bit narrow-scoped. It would be helpful if authors can include quantitative comparisons with the state-of-the-art work including SquiggleFilter and SquiggleNet, and other approaches not based on RUscripts or sDTW (like Minimap2 or UNCALLED). I would also make this paper insightful if the authors can provide a discussion on signal alignment and base alignment.<br><br>They reason why we did not provide comparisons was because none of the existing methods could execute on a resource-constrained device like the one HARU runs on, which has only 4-ARM cores, 4GB RAM, and consumes less than 10W of power (power analysis from post-implementations reports 2.941W for the whole system. See Supplementary Note 8). However, in this revised manuscript, we executed those other methods on high-end devices where possible and compared them to HARU executed on a low-end embedded system. Although the difference in execution platform makes the comparison biased in favour of other methods, our results show that HARU still has better performance.<br><br>We have added those comparisons to Supplementary Note 4 and a brief summary to section Results (page 6) and Methods (Page 9) of the manuscript.<br><br>Q3.10 The authors claim up to 5 parallel query processors can be supported by the tested board, but given the LUT utilization for a single processor, I doubt if 5 instances can actually be placed & routed.<br><br>We thank the reviewer for pointing this out. The doubt regarding whether 5 instances of accelerators can be placed and routed for the target device is valid. To verify the ability to fit multiple accelerators in parallel, we have now included a separate branch (https://github.com/beebdev/HARU/tree/dynamic-reference) that allows for streaming the query and reference signals together, which eliminates the need for storing the reference signal in the FPGA's block RAM. This makes each accelerator independent of each other and does not contain critical paths between the accelerators. Through synthesis and implementation, we see that the most used resource for a single instance of the accelerator is the CLB LUT, taking up to 22% of availability post-synthesis (see Supplementary Note 2, Supplementary Table 1). With this information, we connected four accelerators with a multi-channel AXI DMA and ran synthesis and implementation. Post-implementation resource utilization is shown in Supplementary Table 2 of Supplementary Note 2 and all timing constraints were satisfied. Based on the results we agree that having five accelerators in parallel might be a little bit too tight and require some effort to fit and we have updated the manuscript under "Resource utilization" to change the claim from 5 to 4.<br><br>Q3.11 What observation has led you to conclude that "250 events are adequate for mapping"?<br><br>See the response to Reviewer#1's question Q1.3.1 on this topic and also the newly

Powered by Editorial Manager® and ProduXion Manager® from Aries Systems Corporation

added Supplementary Note 5.

Q3.12 What would you do if the input reads need more than 250 events (e.g. targeting only a specific variant of a virus) to do the selective read alignment?

Targeting a specific variant does not necessarily require more events. In selective sequencing, we take the beginning of the read and not the whole read. The SARS-CoV-2 reference we used was MN908947.3 [https://www.ncbi.nlm.nih.gov/nuccore/MN908947], which is the original Wuhan lineage B. The SARS-CoV-2 SP1 data set we used is B.1 lineage that contained several variants from the reference, however, sDTW could map with >95% accuracy (see Supplementary Note 4). We additionally tested on a sequencing run containing 10 barcoded SARS-CoV-2 samples (a mix of A2.2, B.1 and B.28 lineages) and sDTW could map with >95% accuracy (see Supplementary Note 3). In fact, it is not the query size used for sDTW that matters, but it is the length of the prefix trimmed that is important to properly eliminate the adaptor (and barcode if present) which can be deduced using information available at library preparation (see Supplementary Note 5).

Having said they, if there a specific reasons that require the query length to be larger than 250, users can compile and synthesize HARU with their desired query length and load the system using the xmutil tool on the Kria platform (will consume more area and can be increased up to 1000 events in theory for the Kria FPGA board). However, if we increase it, the accuracy of mapping will increase at cost of adaptive sampling efficiently (number of bases sequenced from unwanted regions; for example rejecting unwanted reads at 250 events at 87% accuracy vs 500 events at 95% accuracy). Also, note that rejecting reads after too many bases have passed through the pore can be detrimental, as long strands passed through the pore when rejected can get clogged and destroy the pore.

Q3.13 Any quantification of the scalability?
For throughput scalability, our hardware architecture is designed with consideration for synthesizing and implementing multiple accelerator instances on the FPGA fabric, which can be streamed with the ARM processor on the MPSoC through the multichannel AXI DMA device as long as the overall resource utilization is below the available resources (approximately 4 on the Kria board). With more parallel instances of the accelerator running on the MPSoC device, we can achieve higher throughput. Additionally, our software implementation uses multithreading for the normalization, and with Kria having quad-core, we can have 4 threads each owning one HARU accelerator. Moreover, since the MPSoC devices are edge platforms, we can deploy clusters of the MPSoC to provide more devices.
Regarding target reference length scalability, we acknowledge that the performance of DTW-based implementations do not scale well with target reference lengths. However, if our work is combined with existing seeding/indexing methods in sigmap or UNCALLED it could be possible to first possible locations on large references through index searching and then use sDTW for refinement. While sDTW remains linear complexity against the reference length, our hardware design and software implementation provide significant acceleration in the search process.
In summary, our work provides insights into both throughput scalability and target reference length scalability through our hardware design and software implementation. While there are limitations to the scalability of DTW-based implementations, we believe that our work provides significant acceleration in the search process, and we are constantly exploring new ways to improve scalability in our future work.

Reviewer #4:
Subsequence Dynamic Time Warping (sDTW) is an important algorithm for processing signals arising from third-generation genomic sequencers. In particular, certain applications with real-time requirements can benefit from efficient computational platforms for this task. The paper adresses this need by presenting a new FPGA-based solution called HARU. It is demonstrated that the approach is energy-efficient and can outperform a self-written C implementation. In addition, HARU is made publicly

Powered by Editorial Manager® and ProduXion Manager® from Aries Systems Corporation

	available which can be beneficial to the bioinformatics community.<br><br>We thank the reviewer for the feedback.<br><br>A weakness of the paper is the limited comparison to the state-of-the-art in the DTW and sDTW area. There are several newer GPU-based and NVM-based approaches that should be considered, such as<br>- Fernandez, et al. "Accelerating Time Series Analysis via Processing using Non-Volatile Memories." arXiv preprint arXiv:2211.04369 (2022).<br>- Schmidt, et al. "cuDTW++: Ultra-Fast Dynamic Time Warping on CUDA-Enabled GPUs." European Conference on Parallel Processing. Springer, Cham, 2020.<br>- Hundt, et al. "Cuda-accelerated alignment of subsequences in streamed time series data." 2014 43rd International Conference on Parallel Processing. IEEE, 2014.<br><br>In particular, I suggest using a measure such as GCUPS (Giga Cell Updates per second) that is commonly used to compare the performance of dynamic programming algorithms on various platforms.<br><br>We appreciate your suggestions for comparison to other state-of-the-art DTW and sDTW implementations. Even though GCUPS or TCUPS is a commonly used performance metric to compare the performance of generic DTW implementations, our work is highly application-specific and focuses on optimizing the selective nanopore sequencing problem for resource-constrained devices. Our work is not meant to be a generic sDTW processor but rather an overarching system architecture composed of an application-specific sDTW processor for selective sequencing purposes. Therefore, the real metric that is relevant to our work is the number of genomic reads processed per second, which we have included in our experimental results. In the updated manuscript we have included comparisons with state-of-the-art solutions exclusively focused on selective sequencing (see the section “Comparison with alternate methods” on page 6 of the manuscript and Supplementary Note 4) We have also added the above papers to the related work section on page 10 of the updated manuscript.
**Additional Information:**	
**Question**	**Response**
Are you submitting this manuscript to a special series or article collection?	No
**Experimental design and statistics**<br><br>Full details of the experimental design and statistical methods used should be given in the Methods section, as detailed in our Minimum Standards Reporting Checklist. Information essential to interpreting the data presented should be made available in the figure legends.<br><br>Have you included all the information requested in your manuscript?	Yes
**Resources**<br><br>A description of all resources used, including antibodies, cell lines, animals and software tools, with enough	Yes

information to allow them to be uniquely identified, should be included in the Methods section. Authors are strongly encouraged to cite Research Resource Identifiers (RRIDs) for antibodies, model organisms and tools, where possible.<br><br>Have you included the information requested as detailed in our Minimum Standards Reporting Checklist?	
**Availability of data and materials**<br><br>All datasets and code on which the conclusions of the paper rely must be either included in your submission or deposited in publicly available repositories (where available and ethically appropriate), referencing such data using a unique identifier in the references and in the "Availability of Data and Materials" section of your manuscript.<br><br>Have you have met the above requirement as detailed in our Minimum Standards Reporting Checklist?	Yes

Powered by Editorial Manager® and ProduXion Manager® from Aries Systems Corporation

Manuscript

Click here to access/download;Manuscript;HARU_gigascience.pdf

OXFORD (GIGA)^n SCIENCE

GigaScience, 2022, 1–13

doi: xx.xxxx/xxxx
Manuscript in Preparation
Paper

PAPER

Efficient Real-Time Selective Genome Sequencing on Resource-Constrained Devices

Po Jui Shih[1,*], Hassaan Saadat[2], Sri Parameswaran[3] and Hasindu Gamaarachchi[1,4,†]

[1]School of Computer Science and Engineering, UNSW Sydney, Australia and [2]School of Electrical Engineering and Telecommunications, UNSW Sydney, Australia and [3]School of Electrical and Information Engineering, University of Sydney, Australia and [4]Kinghorn Centre for Clinical Genomics, Garvan Institute of Medical Research, Sydney, Australia

*pojui.shih@unsw.edu.au
†hasindu@garvan.org.au

Abstract

Background: Third-generation nanopore sequencers offer selective sequencing or 'Read Until' that allows genomic reads to be analysed in real-time and abandoned halfway if not belonging to a genomic region of 'interest'. This selective sequencing opens the door to important applications such as rapid and low-cost genetic tests. The latency in analysing should be as low as possible for selective sequencing to be effective so that unnecessary reads can be rejected as early as possible. However, existing methods that employ subsequence Dynamic Time Warping (sDTW) algorithm for this problem are too computationally intensive that a massive workstation with dozens of CPU cores still struggles to keep up with the data rate of a mobile phone-sized MinION sequencer.
Results: In this paper, we present Hardware Accelerated Read Until (HARU), a resource-efficient hardware-software co-design-based method that exploits a low-cost and portable heterogeneous Multiprocessor System-on-Chip (MPSoC) platform with on-chip Field-Programmable Gate Arrays (FPGA) to accelerate the sDTW-based Read Until algorithm. Experimental results show that HARU on a Xilinx FPGA embedded with a 4-core ARM processor is around 2.5× faster than a highly optimised multi-threaded software version (around 85× faster than the existing unoptimised multi-threaded software) running on a sophisticated server with 36-core Intel Xeon processor for a SARS-CoV-2 dataset. The energy consumption of HARU is two orders of magnitudes lower than the same application executing on the 36-core server.
Conclusions: HARU demonstrates that nanopore selective sequencing is possible on resource-constrained devices through rigorous hardware/software optimisations. The source code for HARU sDTW module is available as open-source at `https://github.com/beebdev/HARU`, and an example application that utilises HARU is at `https://github.com/beebdev/sigfish-haru`.

Key words: selective sequencing; adaptive sampling; nanopore; subsequence dynamic time warping; FPGA; hardware acceleration; edge computing;

Introduction

The latest third-generation nanopore sequencing technology has revolutionised the field of genomics. The portable palm-sized nanopore sequencer called the MinION produced by Oxford Nanopore Technologies (ONT) can perform direct selective sequencing, which rejects the genomic reads that are not of interest. This technique, also known as Read Until, can vastly reduce the sequencing time and cost for applications such as genetic disease identification [1, 2], cancer detection [3, 4], and the surveillance of viruses (e.g. SARS-CoV-2) and other pathogens [5, 6], and sequencing low abundance species metagenomics samples [7]. However, the

Compiled on: April 11, 2023.
Draft manuscript prepared by the author.

Key Points

- Hardware accelerated signal-matching Read Until designed for resource-constrained embedded platforms.
- A resource-efficient sDTW accelerator for selective sequencing.
- Full proposed design (software processing layer, devices drivers, hardware sDTW accelerator): `https://github.com/beebdev/HARU`
- Example application utilising HARU and optimised C implementation of RUScripts: `https://github.com/beebdev/sigfish-haru`.
- Modified RUscripts (supports Python 3.6+, BLOW5 format, ONT's R9.4 chemistry): `https://github.com/beebdev/RUscripts-R9`.

real-time analysis of genomic reads involves the complex and time-consuming process of aligning the read to the reference to obtain the position information. Ideally, the real-time analysis should be performed on a low-cost, low-power, and portable device [8, 9, 10], which is the aim of this paper.

Existing alignment methods for selective sequencing utilise high-performance computing systems to meet the real-time processing requirement, compromising portability, cost-effectiveness, and power efficiency. The very first nanopore selective sequencing method tackled the alignment problem directly in signal-domain [11]. It used *subsequence dynamic time warping* (sDTW) for direct signal mapping for the early R7 nanopore chemistry, which could sequence at a speed of 70 bases/s. However, with the introduction of the R9 nanopore chemistry with a 450 bases/s speed [12], (sDTW)-based Read Until could not keep with a portable palm-sized MinION sequencer, even when running on a 22-core High-Performance Computing (HPC) system. The sDTW computation alone takes more than 98% of the total run time.

The current base-domain Read Until implementations [13] first convert signal reads to bases using GPU-accelerated basecallers, and then map them to the reference base sequence using sequence mapping techniques (e.g. *Minimap2* [14]). Although the mapping techniques in the base-domain are optimised and matured in the bioinformatics field, the prerequisite basecalling step is compute-intensive and is a significant bottleneck Read Until implementations. To keep up with the sequencing rate, the execution of basecalling requires high-end GPU hardware (NVIDIA RTX 1080 for simple reference targets [13] and NVIDIA RTX 3090 for more complex targets [1]), which makes selective sequencing expensive, power-hungry, non-portable and non-scalable. Therefore, researchers have shown significant interest in developing methods to process the raw signals directly (to avoid this compute-intensive basecalling step) and it has become an active and growing research area [11, 15, 16, 17, 18, 19, 20, 21].

In this paper, to address the lack of portability and costly execution nature of existing solutions, we aim to develop a portable, low-cost and power-efficient solution for selective sequencing in raw signal domain. We present HARU (Figure 1), a software-hardware co-design system for raw signal-alignment Read Until that utilises the memory-efficient sDTW hardware-accelerator for high throughput signal mapping.

HARU primarily targets low-cost resource-constrained heterogeneous multiprocessor system-on-chip (MPSoC) devices with on-chip reconfigurable hardware and performs efficient multi-threaded batch-processing for signal preparation in conjunction with the sDTW accelerator. HARU tackles the computational bottleneck by accelerating the sDTW algorithm with Field-Programmable Gate Arrays (FPGAs). The memory-efficient sDTW accelerator for Read Until is designed by exploiting the fine-grained parallelism offered by the FPGA and has a computational time complexity of $O(M+N)$. The sDTW accelerator is loaded onto the on-chip FPGA and interfaces with the software application through software drivers. Sequenced raw-signal samples are pre-processed in software before streaming into the sDTW accelerator (Figure 1). Mapping results of the signal are then returned to the application through the software driver for post-processing.

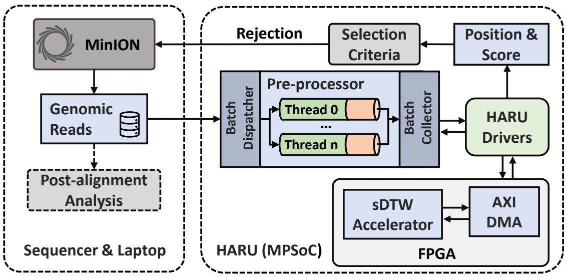

Figure 1. HARU overview.

We demonstrate that HARU gains around 85× speedup against the original software implementation mapping the SARS-CoV-2 sequenced data on a 36-core HPC system. Furthermore, we show that HARU runs around 2.5× faster than an optimised multi-threaded software implementation on the same 36-core server and around 6.5× faster than the same software running on a 10-core Intel Core i9-10850K desktop. The energy consumption of HARU is 341.7 × lower than the same application executing on the 36-core server.

HARU is a complete system for selective sequencing that works on off-the-shelf devices, as opposed to being a conceptual work limited to simulation. For instance, one may purchase the targeted device used in this paper (Xilinx's Kria AI Starter Kit, which has a quad-core ARM Cortex A53 with 4GB of RAM and an on-chip FPGA), flash the device, and execute HARU. In its current form, HARU is limited to kilo-base-sized genomes. However, this is the first time a selective sequencing work is shown to be able to execute selective sequencing on such a low-power and lightweight device, and more importantly, running on off-the-shelf low-cost hardware. HARU demonstrates that selective sequencing can be performed efficiently on an edge device with an excellent price to performance-per-watt ratio. We believe this work will inspire the possibility of performing selective sequencing directly on a chip within a nanopore sequencer.

HARU can also be used as a framework for other future work intending to explore acceleration for selective sequencing on FPGAs by replacing the sDTW core in HARU. As a stepping stone for such projects, this allows quick verification of the experimental core producing practical results instead of being limited to using software simulation. We have provided step-by-step instructions and documentation on building the overarching system from scratch. In addition, the interface to the accelerator is exposed as a library so that the application layer source code can call the interface and treat the accelerator as a black box. We selected Xilinx's Kria AI Starter Kit as the target reference device for HARU, with the intention of HARU being used as a framework for future developers focusing on similar genomics FPGA acceleration work. The *xmutil* tool on Kria platform allows easy access to system performance and information metrics as well as fast loading and replacing of FPGA bitstreams, allowing users to quickly change hardware accelerators for different applications without rebooting the system. Xilinx's Kria supports tools such as Vitis HLS (C to HDL generation) and PYNQ (Python framework for Zynq MPSoCs), which allows researchers with limited hardware backgrounds to design accelerators for their applications.

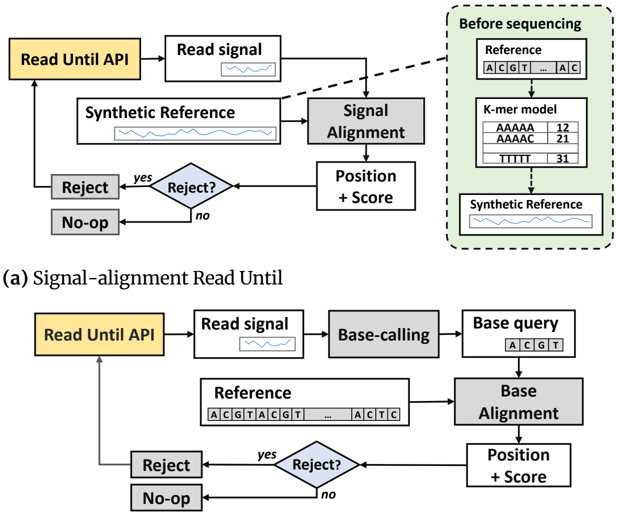

(a) Signal-alignment Read Until

(b) Base-alignment Read Until

Figure 2. Overview of Read Until workflows

Background

Nanopore Selective sequencing

Nanopore sequencers from ONT are third-generation genomic sequencers that are capable of producing long reads (currently ranging between 1 kilo-bases to >2 mega-bases) [22, 23] and are commercially available at an affordable price compared to sequencers of other techniques and generations [24]. These ONT nanopore sequencers provide genomic reads through *flow cells* which contain a proprietary sensor array over nanopore channels embedded in a synthetic membrane [25]. During the sequencing process, the nanopore channels capture the electric current change caused by the genome molecules' ionic current when it passes through [25]. This current signal trace is streamed to the sequencer software in real-time and can later be basecalled into the corresponding nucleobase representation for later analysis [26].

A feature of ONT nanopore sequencers is the direct selective sequencing capability. These sequencers provide real-time data output streams and allow the rejection of reads at individual nanopore channels [11, 13]. This means the sequenced data can be analysed during the sequencing and rejected before completion if decided it is not of interest. This selective sequencing process in the nanopore sequencing workflow is known as *Read Until*. ONT provides the Read Until API interface for software applications to access and reject the sequenced reads in real time. A rejection made through the Application Programming Interface (API) call will eventually be passed back to the sequencer. The voltage at the indicated channel will be reversed to eject the genomic molecule out of the nanopore [11].

For the Read Until execution to be effective, the round-trip task latency for read acquisition, analysis, and rejection signal forwarding should be completed before the majority of the subject read is sequenced by the nanopore sequencer [11]. Rejections made after most of the strand is sequenced bring no benefit as no sequencing time is saved. Existing Read Until methods perform analysis by aligning the genomic reads to the target reference and making the rejection decision based on the position and distance score. This alignment can be done using either signal or base alignment [11, 16, 15, 13, 27, 28].

Signal-alignment Read Until. Signal-alignment Read Until aligns raw signal reads with the reference to obtain the alignment position and distance score, as seen in Figure 2a. Reference sequences usually are obtained in base representation (in the base equivalent '*ACGT*' characters) and need to be converted to a synthetic signal representation before being used to map the reads. This can be done using the k-mer model, which slides a window size of k bases over the base reference while the bases in the window are mapped to a value using the k-mer model hash-map (see Figure 2a). The obtained alignment position and score are then used to determine if a rejection should be made, which is custom to application usage. This signal-alignment workflow was first shown by Loose et al. [11] in the *RUscripts* work, which is also the first Read Until implementation introduced. *RUscripts* is a Python implementation that uses the sDTW algorithm to align initial segments of the raw signals to the synthetic reference and can match 1 read every 0.3 seconds on a single CPU core [11]. At the time of the proposal, *RUscripts* could keep up with the 70 bases/s nanopore sequencing rate on a 22-core server [11]. However, as sequencing speed improved over the years, the current 450 bases/s sequencing rate [12] surpassed *RUscripts*'s capability of performing Read Until during sequencing. We observed that 98% of processing time is spent processing the $O(MN)$ sDTW algorithm.

Base-alignment Read Until. As signal-aligning Read Until could not keep up with improved sequencing rates due to sDTW, researchers turned the focus of Read Until workflows towards base-domain techniques [27, 13]. These techniques align the genomic reads in the base domain as opposed to the signal domain, which requires an extra step of basecalling the signal to base sequences in real-time before alignment (see Figure 2b). Thanks to well-optimised multi-state alignment implementations such as *Minimap2* [14] and the proprietary GPU-accelerated basecaller *Guppy* from ONT, it can out-speed sequencing rate to save time. Recent FPGA acceleration work on Minimap2 [29, 30] could further speedup the base-level alignment. Yet, the extensive power usage and the need for high-performance GPUs and CPUs for basecalling make base-alignment Read Until expensive and non-portable [1].

Potential for signal-alignment Read Until. Alignment in the signal domain and alignment in the base domain share high similarities in their algorithms and mainly differ in the sequence representation [31]. Though base-alignment methods are fast and can keep up with current sequencing rates [13, 1], basecalling is a bottleneck in current base-alignment Read Until methods. Thus, we hypothesise that signal-domain Read Until could reach better performance if enough optimisation and acceleration work is applied to signal-alignment as it does not require the additional basecalling step. In this work, we revitalise the direct signal approach by optimising and exploiting hardware acceleration for the sDTW alignment methodology targeting low-cost embedded heterogeneous platforms, which also addresses the high cost of Read Until executions.

Subsequence Dynamic Time Warping

The dynamic time warping algorithm family are dynamic programming algorithms that provide optimal alignment and distance metrics between two given time series [32] and have been widely used in pattern recognition applications in different fields [33, 34]. This optimal alignment is achieved by warping the time series samples (see Figure 3a), which is done by keeping an $M \times N$ sized cost matrix. The classical DTW (cDTW) algorithm performs global alignment of the signals (see Figure 3b) [32], while the sDTW algorithm performs local alignment of the smaller sequence in the larger sequence (see Figure 3c) [35]. Read Until attempts to find the local alignment of the query on the reference and thus utilises sDTW, which is elaborated below:

sDTW Problem: Given two sequences X of size M and Y of size N where $1 \leq M \leq N \in \mathbb{N}$, the sDTW distance is the summation of the distance in the optimal warp path $w_{optimal}$. The warp paths considered are all the paths that align the sequence X with any subsequence of the sequence Y. The dynamic programming formu-

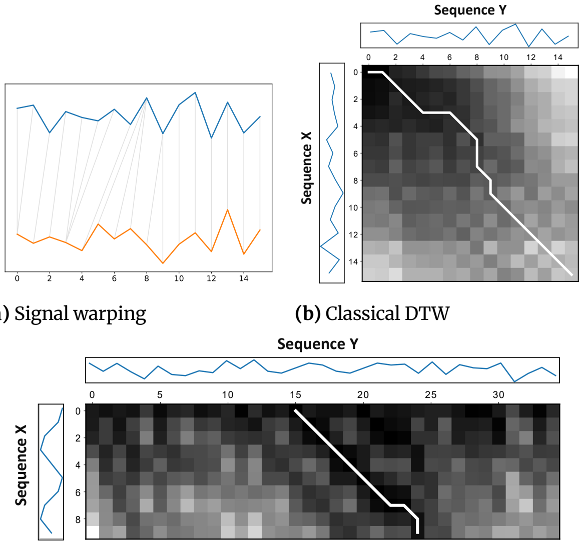

(a) Signal warping **(b)** Classical DTW

(c) Subsequence DTW

Figure 3. Illustration of DTW

lation of sDTW is based on the recurrence relation of the equation:

$$\gamma(i,j) = \delta(i,j) + min \begin{cases} \gamma(i-1,j) \\ \gamma(i-1,j-1) \\ \gamma(i,j-1) \end{cases} \quad (1)$$

where δ is the distance measure[1] between samples and $1 \geq i > M$, $1 \geq j > N$. The boundary conditions for $\gamma(i,j)$ include $\gamma(i,0) = \infty$ and $\gamma(0,j) = 0$ and with a bottom-up memoisation, the γ values are stored in a cost matrix C of size $M \times N$ (i.e., $C[i,j] := \gamma(i,j)$). γ essentially chooses, at each step, the lowest cost move[2]. Once the cost matrix C is populated, the cell with the minimum distance value in the last row would be the ending position of the local alignment. Backtracking from the end position by, again, choosing the step with the lowest cost among the same dependency will give the optimal warp path and starting position (see Figure 3c).

Time and space complexity: The sDTW approach is given in Algorithm 1. As shown, sDTW is $O(MN)$ in time and space complexity due to the 2-dimensional search space. This has led to heavy computational bottlenecks in applications such as *RUscripts* discussed in Section "Nanopore Selective sequencing". To date, not many sDTW optimisation methods exist, and cDTW optimisations such as lower bounding [36, 37] and applying global constraints [38, 39] do not bring many benefits as the necessary search space is much larger than just the diagonal connecting start and end positions of the sequences.

Algorithm 1: Subsequence DTW

```
Input  :X[1 : M], Y[1 : N], M, N
Output :position, score
C: cost matrix of size M * N;
score ← ∞;
position ← −1;
for j in range 1 to N do
  | C[1, j] ← abs(X[1] − Y[j]);
end
for i in range 2 to M do
  | C[i, 1] ← abs(X[i] − Y[1]) + C[i − 1, 1];
end
for i in range 2 to M do
  | for j in range 2 to N do
  |   | d ← min(C[i − 1, j], C[i, j − 1], C[i − 1, j − 1]);
  |   | C[i, j] ← abs(X[i] − Y[j]) + d;
  | end
end
min_score ← C[M, 1]
for j in range 2 to N do
  | if C[M, j] < min_score then
  |   | position ← j;
  |   | score ← C[M, j];
  | end
end
```

Results

Overall system performance

Figure 4a compares the overall performance of HARU for mapping all the 1.382 million reads of the SARS-CoV-2 dataset (see section "Datasets") with software-only implementations. The y-axis of Figure 4a is the signal mapping throughput (mapping throughput is the execution time divided by the number of reads in the dataset). The First bar in Figure 4a represents the original Python-based *RUScripts* (see section "Pure software implementations") running on the HPC with all 36 cores (throughput: 12.52 reads/s). The last bar represents our HARU system with a throughput of 1073.83 reads/s). Thus, our HARU system is ~85.8× faster than the original *RUScripts*. The second bar shows the optimised C implementation of *RUScripts* (see section "Pure software implementations") on the desktop system with a 10-core i9 processor, and the throughput is 162.29 reads/s (HARU is 6.6× faster). Then, the third bar is for the optimised C implementation run with all 36 Xeon cores on the HPC, and the throughput is 432.06 reads/second. HARU system being implemented on a low-cost embedded FPGA system is still ~2.49× faster than the server. The fourth bar in Figure 4a is for the optimised C implementation on the MPSoC run only on the 4-core ARM CPU, which has a throughput of 11.09 read/second. Thus, HARU that utilises the FPGA is 96.8× faster than running on the ARM processor alone.

Similarly, Figure 4b compares the overall HARU performance for mapping all the 500,000 reads of the human dataset to the reference containing the RFC1 gene (see section "Datasets"). HARU (last bar) is 64.5× faster than *RUScripts* on the 36-core HPC (first bar); 5.8× and 4.7× faster than optimised C implementation on the 10-core desktop (second bar) and 36-core HPC (third bar), respectively; and, 66.2× than the optimised C implementation on a 4-core ARM processor (fourth bar) alone.

Note that time measurement for the above throughput calculation for HARU includes all the overheads, including reading signal data from the disk, raw signal pre-processing on software, and data transfer time to/from FPGA for HARU and our FPGA implementation is running at 100 MHz. The speedups observed for HARU over other systems in Figure 4a (SARS-CoV-2 reference) are higher compared to those in Figure 4b (RFC1 reference) because the RFC reference is larger (128Kbases) than the SARS-CoV-2 reference (29Kbases) as explained below.

Performance of the sDTW over reference length

Figure 5 shows how the performance of our sDTW core in HARU executed on the FPGA (including the overhead for data transfer to/from FPGA), and the pure software version of DTW executed on the CPU varies over the reference length. The X-axis is the reference length on a number of bases on the log scale. Y-axis is the time taken for a single sDTW query. For the CPU (red curve), where this y-axis represents the time for executing the sDTW function on a single CPU thread, whereas, for the FPGA (blue curve), this is the time for processing on the FPGA plus the data transfer to and from the FPGA. Observe in Figure 5 how the gap between the

1 Distance metrics in DTW are not limited to a single method. Popular distance metrics include Euclidean distance, squared Euclidean distance, and Manhattan distance.
2 In Equation 1, $\gamma(i-1,j)$ indicates an *insertion* from sequence X into sequence Y whereas $\gamma(i-1,j-1)$ indicates a *match* and $\gamma(i,j-1)$ indicates a *deletion*.

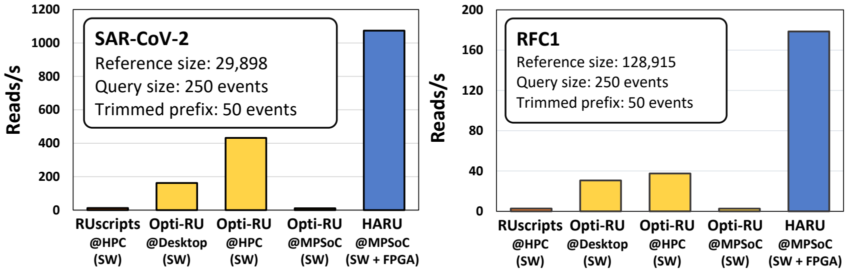

(a) SARS-CoV-2 dataset (b) RFC1 dataset

Figure 4. Mapping throughput for the selective sequencing

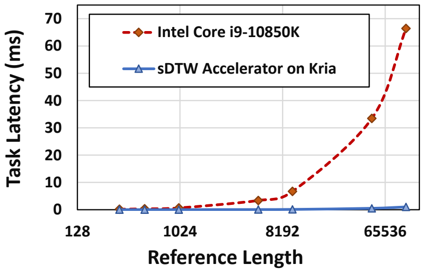

Figure 5. sDTW task latency

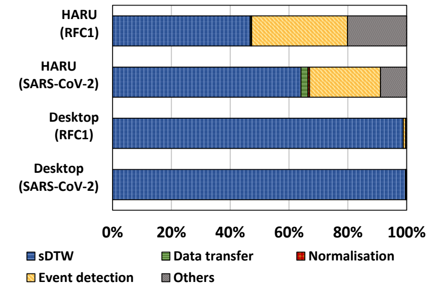

Figure 6. Process time breakdown

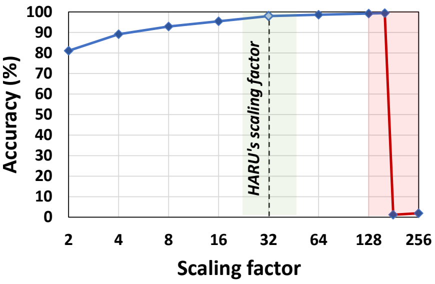

Figure 7. Accuracy against scaling factor

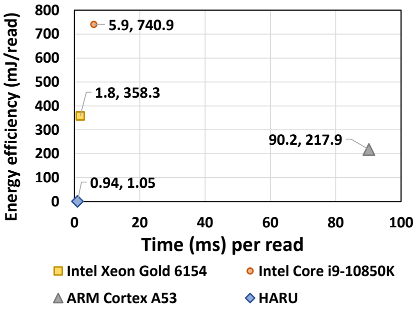

Figure 8. Energy and performance

two curves increases with the reference length, which causes the speed up of HARU over CPU to increase with increased reference size. This behaviour is due to a band of cells being computed in parallel on hardware using a Processing Elements (PE) chain (see section "Resource-Efficient sDTW Accelerator").

The time breakdown for different processing steps

Figure 6 compares the percentage of time spent on different processing steps for HARU vs the optimised software implementation in percentage. Due to the significant speedup of sDTW, the percentage of run time spent on sDTW is <64% for the SARS-CoV-2 dataset and >46% for the RFC1 data set (top two bars), whereas this was >98% for software (bottom two bars). Note that 'others' in Figure 6 is the time spent loading data from the disk, reference preparation, and writing the output.

Accuracy

Figure 7 shows the accuracy of the accelerator using different scaling factors (discussed in section "Software Processing Layer"). Accuracy in Figure 7 is calculated as a percentage of the number of mapping positions similar to results produced from sDTW computed on software using 32-bit floating points. Observe that a scaling factor of 2 yields a limited accuracy (80%), while increasing the scaling factor gradually converges the accuracy towards 100%. However, when scaled above 128, the distance cost accumulation results in data overflow during sDTW, which largely impacts the alignment accuracy. In HARU, we have used a scaling factor of 32 to prevent overflow while having an accuracy close to 99%. Refer to Supplementary Note 1 for further information on using fixed-point and a static scaling factor.

Energy comparison

Figure 8 shows the estimated energy efficiency (y-axis) plotted against the execution time (x-axis) for HARU and optimised software-only implementations on different processors. HARU's overall performance and energy efficiency are considerably lower (close to the origin of the graph: time 0.94 ms/read and energy 1.05 mJ/read) than the optimised version running on ARM (90.2 ms/read, 217.9 mJ/read), Intel Core-i9 (5.9 ms/read, 740.9 mJ/read), and Intel Xeon Gold processor (1.8 ms/read, 358.3 mJ/read). The energy-delay product for the server is 644.94, whereas 0.987 for HARU. Thus, HARU is 650X better in terms of energy-delay products. The energy consumed for HARU and the ARM processor was estimated using the power estimates reported by Vivado in the synthesis report. In contrast, the Thermal Design Power (TDP) value reported in the processor specification was used for Intel processors. For additional power analysis information for the HARU system on the Kria device, please refer to Supplementary Note 8.

Table 1. sDTW Accelerator resource utilisation

Resource	Available	Used (utilisation)
**CLB LUT**	117,120	21,121 (18.03%)
**CLB Registers**	234,240	16,798 (7.17%)
**CARRY8**	14,640	1,787 (12.21%)
**F7 Muxes**	58,560	9 (0.02%)

Resource utilisation

The resource utilisation for our sDTW accelerator, that loads the reference signal to the on-chip block RAM memory before runs, with a single query processor on the Kria board, as reported by the Vivado synthesis report, is shown in Table 1.

Note that we used a single query processor for all the above experiments to show the bare minimum performance on a low-end FPGA platform. As shown in Table 1, the maximum utilisation (CLB LUT) is <20%; thus, in theory, the Kria board can fit up to at least four parallel query processors with some engineering effort. In fact, we have an experimental branch that does not use on-chip block RAM to store references beforehand and directly streams reference signals together with queries. This means multiple accelerators on the same FPGA will not have critical paths in between accelerators. For the post-implementation resource utilisation of four accelerators targeting the Xilinx Kria AI Starter Kit, see Supplementary Note 2.

Comparison with alternate methods

The analysis in the preceding subsections represents the most equitable comparison possible. In this subsection, we attempt to compare HARU with other existing alternate methods. We must acknowledge that making a direct comparison is challenging as different methods are tailored towards different goals and intended for specific systems. Also, it is important to note that each method possesses its own distinct advantage and could be used complementarily.

Comparisons with DeepSelectNet and Guppy+Minimap2

To compare HARU with DeepSelectNet [19] (an enhanced neural-network-based method based on SquiggleNet [20] to classify reads from two classes of species) and the approach used in Readfish [13] (Guppy fast basecalling followed by Minimap2 for mapping), we used a dataset containing reads from two species, SARS-CoV-2 and Yeast (see Methods, Supplementary Notes 3 and 4). DeepSelectNet was executed on a server with a Tesla V100 GPU ()[3] HARU executing on the Xilinx Kria embedded platform (1066.3 reads/s) was yet 2.103× faster (Fig. 9) than DeepSelectNet running on the server (507.1 reads/s). As Guppy binaries for ARM processors are available and Minimap2 can be easily compiled for ARM [40], we executed Guppy_fast+Minimap2 on an NVIDIA Jetson Xavier edge GPU device as Guppy is impractically slow without a GPU (see Methods). HARU was still 3.354× faster than Guppy_fast+Minimap2 (317.94 reads/s). In the Guppy_fast+Minimap2 approach, Guppy took 96.4% of the time, demonstrating that in base-alignment-based selective sequencing methods, base-calling is the bottleneck. The accuracy of HARU (97.41%, Methods, Supplementary Note 4) was better than DeepSelectNet (91.78%) and Guppy_fast+Minimap2 (91.46%).

Note that Python-based DeepSelectNet is a proof of concept design to run on servers and is not optimised for performance. Therefore, the aforementioned numerical values should not be interpreted as definitive, as the method could potentially be optimised for embedded systems. When comparing with Guppy+Minimap2, note that Guppy was executed on a GPU, while HARU is designed for an FPGA architecture. It is possible that implementing Guppy on an FPGA could improve its performance. The accuracy of Guppy+Minimap2 was evaluated using default parameters in Minimap2, and parameter tuning may result in better accuracy. However, such work is beyond the scope of this current study.

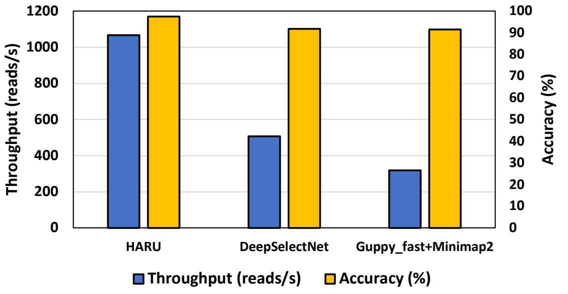

Figure 9. Comparison between HARU and state-of-the-art methods.

Comparison with UNCALLED

To compare HARU with UNCALLED, we mapped SARS-CoV-2 reads to the SARS-CoV-2 reference and compared the mapping location of reads reported by UNCALLED and HARU to Minimap2's mapping (see Methods, Supplementary Note 4). UNCALLED was executed on a Rock64 edge-computing board which has a quad-core ARM Cortex A53 processor with 4GB of RAM, similar to the Kria device used for HARU[4]. HARU's throughput (1066.33 reads/s) is 36.85× higher than UNCALLED on Rock64 (28.94 reads/s). The accuracy of UNCALLED (91.2%) is still lower than HARU's (97.41%).

Note that when comparing UNCALLED with HARU, UNCALLED was executed on the CPU while HARU runs on the CPU and FPGA heterogeneously. The results above must not be wrongly interpreted that UNCALLED is not lightweight, in fact, UNCALLED is much less CPU demanding than sDTW and scales well for larger references. While it is not in the scope of this work, optimising UNCALLED and implementing it on FPGA could yield better results.

Comparison with SquiggleFilter

SquiggleFilter [18] is a conceptual ASIC design for selective sequencing. As it is a conceptual ASIC design work yet to be fabricated and integrated with the envisioned SoC [18], we are unable to compare the performance throughput and accuracy. However, with the provided HDL source code, the resource utilisation of HARU and SquiggleFilter can be compared. We set the SquiggleFilter design to utilise 2000 PEs as claimed in [18], set the target device to the Kria AI starter kit, and manually synthesized the individual modules (as the design does not include a synthesisable top-level module orchestrating all sub-modules). Post-synthesis results show that the PE used in SquiggleFilter requires 2.15× more CLB LUTs (88), 5.81× more CLB Registers (93), and 2.75× more CARRY8 resources than HARU's PE in the sDTW accelerator (41, 16, and 4 respectively). As SquiggleFilter requires 2000 PEs for one single tile of accelerator (while HARU requires only 250 as it uses events), the warper in SquiggleFilter requires 8.44× more CLB LUTs (178,553), 11.54× more CLB Registers (191,991), and 12.5× more CARRY8s (22,002) than the total resource utilisation of HARU's sDTW accelerator (21158, 16634, and 7160 respectively). Note that this comparison for SquiggleFilter is excluding the normaliser, mean finder, mad finder, and it's a top-level entity. See Supplementary Note 4 for a more detailed resource comparison.

We also note that although claimed to be verified on FPGA, SquiggleFilter is primarily an ASIC design work. The results above targets the Kria AI Starter Kit device that HARU uses and synthesis results may differ based on target devices. Nevertheless, HARU shows to have an advantage over SquiggleFilter when targeting FPGAs for deployment with its much more efficient resource utilisation. In addition, HARU is a complete system integrated with off-the-shelf hardware devices with software support.

Methods

Design of Hardware Accelerated Read Until

HARU targets low-cost MPSoCs with on-chip FPGA to perform selective sequencing processing. Figure 10 shows the architecture of HARU in an ONT nanopore sequencing workflow. HARU consists of three main components: the software processing layer, device drivers for the accelerator and associated hardware, and the hardware sDTW accelerator. The software processing layer, discussed in subsection "Software Processing Layer", uses a multi-threaded batch processing architecture to perform raw read signal pre-processing and is customizable based on the selection criteria. The device drivers, discussed in subsection "HARU Device Drivers", are designed to provide high-throughput data transferring of query and reference signals. Lastly, the resource-efficient sDTW accelerator, discussed in subsection "Resource-Efficient sDTW Accelera-

3 Without a GPU, neural-network-based methods will be impractically slow. The proof-of-concept DeepSelectNet implementation is not supported on edge GPUs.

4 UNCALLED has many software dependencies and requires a package manager, which is not available on the Kria device running PetaLinux, see Methods.

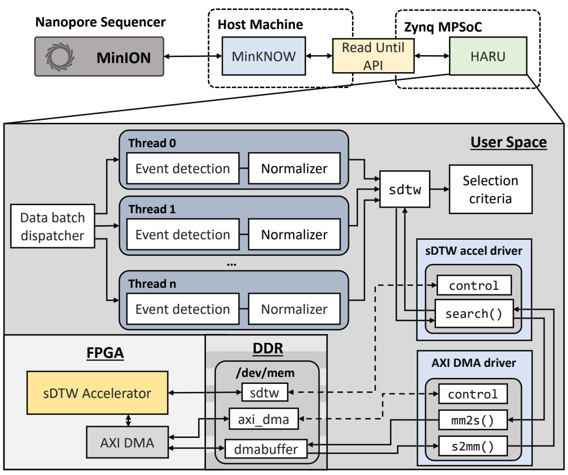

Figure 10. HARU architecture.

tor", performs high throughput sDTW for the selective sequencing use case.

Software Processing Layer

The software processing layer of HARU is the front end of the HARU design running on the processing system on the MPSoC. Its main tasks include pre-processing the reference sequence and raw signal reads and the final selection decision. Since references are obtained in base representation as discussed in subsection "Nanopore Selective sequencing", the initialisation step of the software processing layer forms the synthetic reference signal for the forward and reverse representation of the base reference[5] using the k-mer model for the flowcell type. Then, in preparation for the sDTW computation in hardware, the reference signal is normalised using z-score normalisation. Since the data type used for the signal and cost matrix in the sDTW accelerator are 16-bit fixed-point types (discussed in the section "Resource-Efficient sDTW Accelerator"), the normalised values are scaled with a scaling factor to preserve signal resolution.

During the genome sequencing step, the software layer collects sequenced data from the nanopore sequencer in batches which is then dispatched into multiple threads for efficient computing of pre-processing (see Figure 10). Each thread performs event detection on the raw signal samples to reduce sample data size for the sDTW accelerator. This is done until enough events are collected. For the R9.4 flowcell, 250 events are typically adequate for mapping and would require roughly 0.4-0.8 seconds of data collection[6]. After the collection, the events are normalised and scaled with the same scaling factor used in the reference signal preparation. When threads finish the pre-processing, the processed data are gathered and sent to the sDTW accelerator for processing using the drivers. After which, the mapping position and the similarity score are used to decide whether the read should be rejected.

HARU Device Drivers

To control and utilise the hardware accelerator in the software processing layer, we designed the software device drivers to have two main data paths (see Figure 10). The first data path is the control path of the accelerator, which uses the AMBA AXI4-Lite protocol to configure the control registers and read status registers in the software. The accelerator's physical address is memory-mapped to the virtual address space for user space applications to utilise.

The second data path is for the query and reference sequences.

5 This is needed since DNA molecules are double-stranded.
6 time to obtain around 50-300 events that belong to the read adapter and then actual 250 events of the query. See Supplementary Note 5 for more information.

Algorithm 2: Memory-efficient subsequence DTW

Input : $X[1:M], Y[1:N], M, N$
Output : *position*, *score*

```
1  C: array of size M + 1 initialised to ∞;
2  score ← ∞;
3  position ← −1;
4  for j in range 1 to N do
5      n ← 0;
6      nw ← C[1];
7      w ← C[2];
8      for i in range 1 to M do
9          C[i] := abs(x[i] − y[j]) + min(n, nw, w);
10         n := C[i];
11         nw := w;
12         w := C[i + 2];
13     end
14     if C[M] < score then
15         position ← j;
16         score ← C[M];
17     end
18 end
```

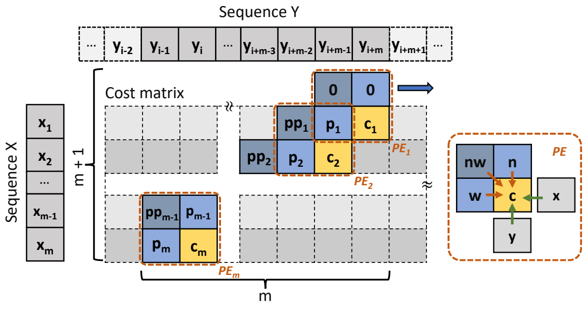

Figure 11. Pipelined execution of Algorithm 2

To prevent data transfer from becoming a bottleneck, we use the AMBA AXI4-Stream protocol to stream query and reference data into the accelerator at a high-throughput rate. This is done by using AXI Direct Memory Access (DMA) module to point to a physical hardware address to stream data to and from. By calling the driver function for processing the query, the sDTW accelerator driver initiates the transfer from the query and reference buffers to the transfer buffer on Double Data Rate (DDR) memory dedicated to AXI-stream communication and the FPGA. Our benchmarks show that data can be sent to and from the accelerator at a throughput of 330 MB/s.

Resource-Efficient sDTW Accelerator

As discussed in the section "Subsequence Dynamic Time Warping", the standard sDTW algorithm has $O(MN)$ time and space complexity due to the computation of the cost matrix. The computation of a cell value in the cost matrix requires comparing three neighbour cell values, making the exploitation of available hardware parallelism harder. Also, the preservation of the full cost matrix does not scale well if directly implemented on resource-constrained FPGA devices. We identified that the backtracking of the cost matrix to obtain the warp path is unnecessary for Read Until as the ending position is adequate to make the rejection decision. We provide the following optimisations over sDTW to obtain a resource-efficient high-throughput sDTW accelerator.

Cost matrix memory optimisation. The need to preserve the $M \times N$ sized matrix for backtracking was discussed in section "Subsequence Dynamic Time Warping". However, for selective sequencing, the obtained end position of the alignment is adequate to determine the location of the current query; thus, the backtracking step for obtaining the starting position is unnecessary. Consequently, preserving the whole cost matrix values is unnecessary, and a cost array of $M + 1$ is sufficient. Algorithm 2 shows the sDTW algorithm after the cost matrix size is reduced. The outer loop (line 4 of Algorithm 2) iterates through the whole reference sequence, while

the nested inner loop (line 8 of Algorithm 2) iterates through the column at each reference sample. During each iteration of the inner loop, the computation of the recurrence equation is performed, and the computed value is stored in the cost array that is of the same size as the query. Once the inner loop completes, the current minimum score and position values are updated if the last cell of the cost matrix is smaller than the current minimum score. As the computation is done in exactly the same way as the original sDTW with the whole cost matrix, there is no impact on accuracy from this optimisation.

Operation pipelining. The sDTW cost matrix size reduction explained above optimises the space complexity of the computation for selective sequencing. However, the algorithm's execution is still sequential and has $O(MN)$ time complexity. Computing the whole column in parallel by unrolling the inner loop is not feasible due to the data dependency in the recurrence equation that needs waiting until the n value is ready (see Algorithm 2). We observe that once the first iteration of the inner loop for the column is completed, all data dependencies for the first inner loop iteration for the next column are ready. By pipelining the outer loop computation, an oblique column is formed that is computed in parallel, as shown in Figure 11. This oblique column traverses through the reference sequences, reducing the time complexity from $O(MN)$ to $O(N)$ since the N query size is now computed in parallel. Since all cell computations are computed only after the dependencies are satisfied, pipelining does not affect the accuracy of sDTW.

Fixed-point data representation. After the optimisation above, the hardware's computational complexity is $O(M)$. However, the actual time needed is $(M + N - 1) \times II$, where II is the initiation interval (i.e. the number of cycles between loop iterations). In pipelined Algorithm 2, II is how fast the reference equation $C[i] := abs(x[i] - y[j]) + min(n, nw, w)$ can be computed. Normally, 32-bit floating-point data types are used for the sDTW computation to preserve the precision after the sequences are normalised. This is expensive to implement in hardware regarding resources and execution time. By using a fixed-point representation with fewer data bits and scaling the sequence values using a scaling factor, the recurrence equation can be computed in hardware rapidly and efficiently while keeping sufficient precision. We chose 16-bit fixed-points with a scaling factor of 2^5 as it gives sufficient precision and keeps II at one clock cycle (see section 22 on accuracy). Using fixed-point with a static scaling factor will decrease the accuracy slightly as we are using fewer bits to represent the decimal points compared to floating points. Nevertheless, this data representation will still provide close to zero difference in mapping accuracy compared to using floating points (see Supplementary Notes 1 and 6 for more detail).

HARU's sDTW Accelerator. The oblique parallel-computed column mentioned above uses a PE-chain structure where data-dependent neighbour cells are shared amongst the PEs (Figure 11). As shown in Figure 12, the shared values are stored in two register arrays of size M (L1 being the previous cost array and L2 being the second previous cost array). At each iteration, the costs in the L1 array are shifted into the L2 array, while the current costs are passed onto the L1 array. Each PE computes the recurrence equation, which takes the Manhattan distance ($\delta = |x[i] - y[j]|$) and adds the minimum of the three neighbour cells (see equation 1). Samples of the reference sequence are first streamed into the first PE of the chain and are then passed along to successive PEs in each iteration. In the subsection "Software Processing Layer", we discussed that the software processing layer uses multi-threaded batch processing to perform event detection and normalisation. The event detection decreases the query size to make the M term smaller in the algorithm complexity. We choose to use a size of 250 events (see Supplementary Note 5), giving the accelerator a PE chain of 250 PEs. In total, it takes $N + 250 - 1$ clock cycles to complete the full search.

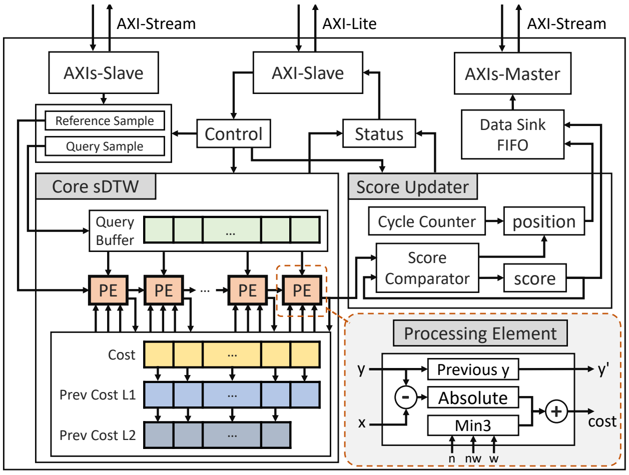

Figure 12. sDTW hardware accelerator design for HARU

Table 2. Computational platforms

System	HPC	Desktop	MPSoC
CPU	Intel Xeon Gold 6154	Intel Core i9-10850K	Arm Cortex-A53
CPU cores	36	10	4
Clock rate	3.00 GHz	3.60 GHz	1.5 GHz
RAM	377 GB	32 GB	4 GB
FPGA	No	No	Yes

Table 3. Datasets

Target	SARS-CoV-2	RFC1
Type	Viral genome	Partial human genome
No. of bases	29,903	128,915
Search space size	59,806	257,830
No. of reads	1,382k	500k
SLOW5 file size	5.5 GB	39 GB

Experimental Setup

The HARU system, proposed in the section "Design of Hardware Accelerated Read Until", was implemented on Xilinx's Kria AI Starter Kit with a Zynq Ultrascale+ XCK26-SFVC784-2LV-C MPSoC. This board contains a processing system with a quad-core ARM Cortex A53 CPU and 4GB of DDR4 memory (specifications on column 'MPSoC' in Table 2). Implementation details of HARU will be discussed in the section "HARU implementation". This HARU implementation is compared to two pure software implementations discussed in section "Pure software implementations". These two software versions are executed on a desktop computer comprising of a 10-core Intel Core-i9 processor and a high-performance computer (server) with a 36-core Intel Xeon processor (specifications are in Table 2). We performed the experiments on two representative datasets detailed in the section "Datasets.

HARU implementation

The operating system running on the processing system of the board is a customised embedded Linux image generated using Xilinx's Petalinux 2021.1 tool. To show the bare minimum throughput of the accelerator, our sDTW accelerator is synthesised with a single query processor in the accelerator clocked at 100 MHz. The number of query processors that can fit in the FPGA depends on the available resource on the device; see section "Resource utilisation" for resource utilisation of the accelerator with a single query processor.

sDTW Hardware Accelerator: The sDTW accelerator was implemented using Verilog Hardware Descriptive Language (HDL). Synthesis was performed using Xilinx's Vivado 2021.1. The control bus interface for the accelerator utilises the AMBA AXI-Lite pro-

tocol. We utilise the AMBA AXI-Stream protocol through the AXI DMA hardware in the FPGA for high-throughput data transfer for the query and reference data.

HARU Driver: Device drivers were implemented for the hardware accelerator and AXI DMA in the C programming language. The accelerator and AXI DMA's drivers memory map the physical address of corresponding devices into the virtual address space for utilisation by the user space applications. The shared communication memory buffers between software and FPGA are preserved on the DDR memory, which is allocated during the initialisation stage.

Software Processing Layer: The software processing layer that prepares the raw signals and performs the selecting decision was implemented in the C programming language. For benchmarking experiments, the software loads raw signal data in the BLOW5 format [41] from a USB 3 external hard drive connected to the Kria board. Raw signals for a batch of reads are first loaded to the Random Access Memory (RAM) and are pre-processed using multiple threads implemented using POSIX threads. Pre-processing steps include event detection, prefix trimming, and normalisation (explained in section "Software Processing Layer"). Then, sDTW is performed on each read in hardware by iteratively calling the HARU driver. Once the mapped positions and the DTW scores are available for the whole batch, the software computes the mapping quality (MAPQ) [42] and executes the selection criteria based on this score [11].

Pure software implementations

RUscripts: Original *RUscripts* written by Loose et al. [11] using Python 2.7 has reached end-of-life support and target ONT's R7 Nanopore chemistry, which is no longer in use. We modified *RUscripts* to work on Python 3.6+ and extended it to support BLOW5 format and ONT's current Nanopore chemistry R9.4. This support for R9.4 chemistry is implemented by integrating the R9.4 pore model and R9.4 event detection parameters [43, 44].

Optimised *RUscripts* in C: As the Python *RUscripts* is not efficient enough for a fair comparison, we implemented a multi-threaded C implementation that follows similar algorithmic steps. This implementation in C is very similar to the software explained above (section "Software Processing Layer") except that sDTW on the CPU is called with multiple threads instead of using the FPGA accelerator. The sDTW computation on the CPU is performed using the optimised sDTW implementation in the *mlpy* library [35].

Datasets

HARU was tested against combinations of software running on the systems mentioned in Table 2 on two datasets. The first dataset is the SARS-CoV-2 genomic reads sequenced on a MinION R9.4 flowcell and has a total of 1.382 million reads (Table 3), publicly available at [45]. The SARS-CoV-2 genome (MN908947.3), which is 29903 bases long, is used as the reference for this experiment. The second dataset is a subset of a NA12878 human genome reference sample containing 500,000 reads sequenced on a PromethION R9.4 flowcell (Table 3), publicly available at [41]. This dataset is mapped to a reference constructed by extracting the region chr4:39262456-39391375 (128 Kbases long) of the human genome (hg38). This region includes the RFC1 gene, which contains an important pathogenic variant indicative of hereditary cerebellar ataxias disease and selective sequencing has been applied [1] for accurate diagnosis.

Performance Evaluation

We measure the overall execution time of mapping all reads of the provided datasets by using the *gettimeofday* function in C. This execution time is divided by the number of reads in the dataset to calculate the signal mapping throughput. Note that all our time measurement used in throughput calculation includes all the overheads including reading signal data from the disk, raw signal preprocessing on software, and data transfer time to/from FPGA for HARU.

Comparison with alternate methods

To compare HARU with DeepSelectNet and the approach in Readfish (Guppy2 followed by Minimap2), we used the curated test data for SARS-CoV-2 and Yeast from [19]) that contained 20,000 reads from each species. DeepSelectNet was installed and executed on a workstation with a Tesla V100-16GB GPU, 20 CPU cores, and 384 GB RAM (Supplementary Notes 3 and 4). Guppy 6.1.3 and Minimap2 2.20 were set up and run on an NVIDIA Jetson Xavier AGX board. Note that we activated the 15W nvpmodel on this device to make the specification closer to what is available on the Kria board used for HARU. The *dna_r9.4.1_450bps_fast.cfg* model was used for Guppy and a combined reference genome of SARS-CoV-2 and Yeast was used for Minimap2 (Supplementary Note 7). For DeepSelectNet, the first 4500 signal samples were used (default options) and the same number of signal samples were used for Guppy+Minimap2. For HARU we used the default prefix and query size in HARU (50+250) that approximately relates to around 1500-3000 signal samples. The accuracy of each method was calculated as the sum of true positives and true negatives divided by the total reads (Supplementary Note 4). For HARU where the reads from each species were mapped against the SARS-CoV-2 reference, the cut-off value for sDTW scores to determine if a read mapped to SARS-CoV-2 or not, was determined as explained in Supplementary 3.

To compare against UNCALLED, we used 40,000 reads from the SARS-CoV-2 dataset (in Subsection 4). UNCALLED was installed on a Rock64 embedded device that has a similar computing power (quad-core ARM Cortex A53 with 4GB RAM) to the Kria board used for HARU. This is because UNCALLED has many dependencies and enabling support for the Kria platform which runs a custom PetaLinux distribution is laborious. Despite the Rock64 board supporting Ubuntu and the apt package manager along with Python/PIP and C/C++ build tools, we still had to manually intervene in the UNCALLED installation scripts to enable support for HDF5 and BWA dependencies to build on ARM. Both HARU and UNCALLED were executed using the SARS-CoV-2 reference and the accuracy was calculated by using UNCALLED pafstats by comparing mapping locations to Minimap2 mappings as the truth set (Supplementary Note 7). The *−chunk-time* and *−max-chunks 1* parameters in UNCALLED was used to limit the number of signal samples to 3200 (Supplementary Note 7). For generating the truth set using Minimap2, the complete reads were basecalled and mapped.

Discussion

Signal-level vs base-level selective sequencing

The field of selective sequencing is a nascent area, and to date, no definitive solution has emerged as the panacea. Both signal-level and base-level approaches to selective sequencing have advantages and disadvantages and determining which is the optimal approach at this stage is more of a philosophical debate.

With the methods available to date, basecalling raw signals obtained from the sequencers to convert signals to base-domain followed by using optimised alignment tools such as Minimap2 (the approach described in Readfish) is the most practical approach if large genomes are involved. The reason being base-level aligners have matured over the last decade of research and development and are highly optimised to make base-level selective sequencing practical. However, for basecalling, regardless of the GPU acceleration effort performed by ONT over the years, basecalling is still the major bottleneck for base-domain selective sequencing, taking 96% of the execution time for Guppy fast basecalling + Minimap2. Furthermore, basecalling is not portable nor scalable due to the compute power constraints, and if selective sequencing is ever to be done on an integrated chip within the sequencer, basecalling approaches would require a more costly system and possibly come at a much larger form factor.

The goal of signal-level selective sequencing is to completely bypass the basecalling step and instead, directly map the raw signal to the reference. This is an emerging and immature field and will inevitably require a substantial period of time to achieve the same level of maturity as base-level selective sequencing. Since the concept of nanopore selective sequencing was introduced, a range of different signal-level selective sequencing methods was explored, including RUscripts [11], cwDTW [17], UNCALLED [15], and sigmap [16]; and more recently, RawHash [21], DTWax [46], and RawMap [47].

In addition, directly passing raw signals into neural networks is also being explored as opposed to using classical algorithms for mapping, including works such as SquiggleNet [20], DeepSelectNet [19], and RISER [48]. However, neural-network-based approaches are currently limited to classifying reads between two target species, and getting mapping coordinates is not yet possible. Moreover, neural-network-based methods require training the model for each dataset, which makes it less flexible and require more preparation than the classical approaches.

The data rate of nanopore sequencers is comparable to modern camera sensors on mobile devices today. Considering the amount of raw signal processing being performed for sensors on mobile devices, it is promising to envision signal-level nanopore selective sequencing done efficiently within nanopore sequencers, if this level of miniaturisation is ever reached for selective sequencing compute requirements. In summary, signal-level selective sequencing is an exciting area worth investigating together with base-level selective sequencing.

Limitations and Future Work

In our proof-of-concept implementation of HARU, the reference sequence is first loaded onto the FPGA's on-chip memory (block RAM) at the beginning of the execution. During alignment, the PE chain streams the reference samples from the block RAM to the first PE (Fig. 12). On-chip memory (block RAM) on the Xilinx Kria board used for evaluation is limited to 5.1 Mb, thus limiting the maximum reference sequence size to 295 kilobases. To eliminate this limitation, future work could directly stream the reference together with the query sequence before each sDTW call (there is currently an experimental branch for this, see Supplementary Note 2). However, even with HARU (linear time complexity for sDTW), performing sDTW of a query on a giga-base-sized genome like the human genome is impractical (estimated to take 3 seconds for a query). Nevertheless, when processing giga-based sized genomes, HARU is intended to be used in the final refinement step when potential mapping locations (a few reference sequence segments that are small in size) are first found using a heuristic method. Such a heuristic method that can currently map nanopore signals directly to giga-based sized genomes does not exist. However, methods such as Sigmap [16], UNCALLED [15] and RawHash [21] are already setting the foundation for scalable direct signal mapping.

Future work can also improve the throughput by implementing multiple parallel sDTW cores for coarse-grain parallelism. Our sDTW processor uses less than 20% of the LUT resources of the FPGA, as mentioned in Section "Resource utilisation". Thus, resources are sufficient to fit multiple parallel processors, increasing the theoretical throughput. A high-end FPGA board with a larger area could support even more processors; for instance, Xilinx's Versal VP2802 FPGA has sufficient resources to theoretically fit 140 parallel processors (see Supplementary Note 2 for experimental explorations of deploying four accelerators in HARU). However, such work also would require eliminating other bottlenecks that would arise. For instance, the 30% of execution time currently spent on the signal pre-processing (Fig. 6) would then become a bottleneck and require acceleration.

Our implementation of HARU loads raw signal from BLOW5 file format because the slow5lib library is lightweight (with minimal dependencies), thus, easily allowing the cross-compilation to target the Kria platform. Running MinKNOW on the Kria platform is theoretically possible but is far from practicality due to being closed source. Even if MinKNOW were open source, potential issues with hundreds of bulky dependencies would make cross-compilation impractical. Potential workarounds could include a server-client approach where MinKNOW runs on a laptop and communicates with the Kria board using ethernet. However, such workarounds are not ideal due to network communication overheads. Also, latency in the public-facing ReadUntil API provided by ONT (in Python programming language) would negate the massive benefit of hardware acceleration.

Our proof-of-concept HARU implementation is currently limited to DNA on R9.4 chemistry. Future work could focus on extending selective RNA sequencing, the most recent R10.4 chemistry, or upcoming protein sequencing from ONT.

The primary sequencer device targeted for HARU running on resource-constrained devices is the palm-sized MinION nanopore sequencer. Sequencers such as ONT's PromethION provide a much larger throughput than MinION and will vastly increase the selective sequencing processing requirements. Future work could explore the scalability of HARU on higher-end FPGAs with HBM memory and more resources for fast selective sequencing on high-throughput sequencers such as the PromethION.

Related Hardware Acceleration Work

Existing hardware acceleration work targeting the subsequence search problem using the DTW algorithm family is rare. Previous FPGA accelerators such as [49, 50] implement a cDTW accelerator to compute the distance score between a query and a window buffer that stores a subset of the reference sequence. The reference sequence is continuously streamed into the window after each cDTW compute iteration completes, shifting older samples out. A distance score that is below a preset threshold indicates a match between the query and the current reference subsequence in the window buffer. [49] focused on exploiting coarse-grain parallelism by computing multiple cDTW in parallel. [50] introduced a PE-ring structure that computes multiple recurrence equations in parallel where the processing elements (PEs) compute cells that do not share data dependencies. This windowed cDTW approach is suitable for reference sequences of undetermined arbitrary length. Still, it is inefficient (requires $N \times O(M^2)$ for software approaches) for selective sequencing usage where the reference sequence is static with a known length. sDTW, on the other hand, is a data-reusing version of the approach and our work exploits the fine-grain parallelism that computes the whole $O(M)$ dimension in parallel, leaving $O(M+N)$ computational time and $O(M)$ space. Furthermore, there is prior work that accelerates DTW using non-volatile memories [51] and using GPU acceleration [52, 53].

For the hardware acceleration on signal-alignment Read Until the only previous attempt was a simulated Application Specific Integrated Circuit (ASIC) design that accelerates the sDTW algorithm [18]. The proposed accelerator uses the unprocessed raw signal reads to map directly with the reference, which requires 2000 PEs to perform the matching and has a reference limit of 100KB. The design has extensive resource requirements making it difficult to fit on lower-cost reconfigurable hardware, thus targeting ASIC. Furthermore, as seen in the history of Read Until [11, 27, 13], Read Until requires implementations to adapt quickly as nanopore sequencing technology improves, and the cost of re-manufacturing ASICs would be unsustainable. In contrast, HARU is a complete design with an efficient software processing layer utilising the sDTW accelerator. Our presented accelerator requires only 250 resource-efficient PEs due to pre-processing reducing the query size needed in the high-throughput computation of sDTW and is capable of

executing selective sequencing with low-cost embedded MPSoC platforms with on-chip reconfigurable hardware.

Conclusion

Existing sDTW-based software methods for nanopore selective sequencing are highly computationally intensive, and a large workstation cannot keep up with a portable MinION sequencer. In this paper, we present HARU, a resource-efficient design that enables sDTW-based selective sequencing on a low-cost and portable heterogeneous system comprised of an ARM processor and an FPGA, which is around 85× faster than the original sDTW-based software implementation and around 2.5× faster than a highly optimised software version running on a server with a 36-core Xeon processor for a complete SARS-CoV-2 dataset. The energy-delay product for the server is around 650× higher than HARU executing on an embedded device.

Availability of source code and requirements

HARU

- Project name: HARU
- Description: Source code for the HARU accelerator, including the Verilog HDL core accelerator and user-space device driver
- Project home page: https://github.com/beebdev/HARU
- Operating system(s): Windows 10/11 (building), Custom Embedded Linux image built with PetaLinux 2021.1 (running)
- Programming language: Verilog, C, Python
- Other requirements: Vivado 2022.2, Petalinux 2021.1
- License: MIT

Sigfish-HARU

- Project name: Sigfish-HARU
- Description: Source code that demonstrates the proof-of-concept integration of HARU accelerator for squiggle mapping. Also contains the optimised RUscripts implementation in C.
- Project home page: https://github.com/beebdev/sigfish-haru
- Operating system(s): Linux (building), embedded Linux built with PetaLinux 2021.1 (running)
- Programming language: C
- Other requirements: Cross-compilation toolchain for AARCH64
- License: MIT

RUScripts-R9

- Project name: RUScripts-R9
- Description: The modified RUscripts to support Python 3.6+, BLOW5 format and ONT's current Nanopore chemistry R9.4
- Project home page: https://github.com/beebdev/RUscripts-R9
- Operating system(s): Platform Independent
- Programming language: Python
- Other requirements: Python 3.6
- License: MIT

Availability of supporting data and materials

Datasets used for the benchmarks are available to be directly downloaded from https://doi.org/10.5281/zenodo.7314838, which we curated from publicly available datasets:

- https://community.artic.network/t/links-to-raw-fast5-fastq-data-for-artic-protocol/17 associated with publication [45]
- https://www.ncbi.nlm.nih.gov/sra/SRX11368475 associated with publication [41]

Declarations

List of abbreviations

API: Application Programming Interface
ASIC: Application-Specific Integrated Circuit
AXI: Advanced eXtensive Interface
cDTW: Classical Dynamic Time Warping
CLB: Configurable Logic Block
CPU: Central Processing Unit
DMA: Direct Memory Access
DTW: Dynamic Time Warping
FPGA: Field-Programmable Gate Arrays
GPU: Graphics Processing Unit
HBM: High Bandwidth Memory
HDL: Hardware Descriptive Language
HPC: High-Performance Computing
LUT: Lookup Table
MAPQ: Mapping Quality
MPSoC: Multi-Processor System-on-Chip
ONT: Oxford Nanopore Technologies
PE: Processing Element
RAM: Random Access Memory
sDTW: Subsequence Dynamic Time Warping
TDP: Thermal Design Power

Consent for publication

Not applicable.

Competing Interests

H.G. has received travel and accommodation expenses to speak at Oxford Nanopore Technologies conferences. The authors declare no other competing interests.

Funding

H.G. is supported by Australian Research Council DECRA Fellowship DE230100178.

Author's Contributions

H.G., H.S., and S.P. conceived the work. P.S. and H.S. designed and implemented the hardware accelerator. P.S. designed and implemented the device driver. H.G. designed and implemented the optimised multi-threaded signal mapping software (*sigfish*). P.S. drafted the manuscript. H.G. and S.P. revised the manuscript. H.G. and P.S. devised the experiments and benchmarks. P.S. conducted the experiments and benchmarks. All authors read and approved the manuscript.

References

1. Stevanovski I, Chintalaphani SR, Gamaarachchi H, Ferguson JM, Pineda SS, Scriba CK, et al. Comprehensive genetic diagnosis of tandem repeat expansion disorders with pro-

grammable targeted nanopore sequencing. Science Advances 2022;8(9):eabm5386. https://www.science.org/doi/abs/10.1126/sciadv.abm5386.
2. Miller DE, Sulovari A, Wang T, Loucks H, Hoekzema K, Munson KM, et al. Targeted long-read sequencing identifies missing disease-causing variation. The American Journal of Human Genetics 2021;108(8):1436–1449.
3. Djirackor L, Halldorsson S, Niehusmann P, Leske H, Capper D, Kuschel LP, et al. Intraoperative DNA methylation classification of brain tumors impacts neurosurgical strategy. Neuro-Oncology Advances 2021;3(1):vdab149.
4. Yamaguchi K, Kasajima R, Takane K, Hatakeyama S, Shimizu E, Yamaguchi R, et al. Application of targeted nanopore sequencing for the screening and determination of structural variants in patients with Lynch syndrome. Journal of Human Genetics 2021;66(11):1053–1060.
5. Wang M, Fu A, Hu B, Tong Y, Liu R, Liu Z, et al. Nanopore targeted sequencing for the accurate and comprehensive detection of SARS-CoV-2 and other respiratory viruses. Small 2020;16(32):2002169.
6. Marquet M, Zöllkau J, Pastuschek J, Viehweger A, Schleußner E, Makarewicz O, et al. Evaluation of microbiome enrichment and host DNA depletion in human vaginal samples using Oxford Nanopore's adaptive sequencing. Scientific reports 2022;12(1):1–10.
7. Martin S, Heavens D, Lan Y, Horsfield S, Clark MD, Leggett RM. Nanopore adaptive sampling: a tool for enrichment of low abundance species in metagenomic samples. Genome Biology 2022;23(1):1–27.
8. Quick J, Loman NJ, Duraffour S, Simpson JT, Severi E, Cowley L, et al. Real-time, portable genome sequencing for Ebola surveillance. Nature 2016;530(7589):228–232.
9. McIntyre AB, Rizzardi L, Yu AM, Alexander N, Rosen GL, Botkin DJ, et al. Nanopore sequencing in microgravity. npj Microgravity 2016;2(1):1–9.
10. Samarakoon H, Punchihewa S, Senanayake A, Hammond JM, Stevanovski I, Ferguson JM, et al. Genopo: a nanopore sequencing analysis toolkit for portable Android devices. Communications biology 2020;3(1):1–5.
11. Loose M, Malla S, Stout M. Real-time selective sequencing using nanopore technology. Nature methods 2016;13(9):751–754.
12. Wang Y, Zhao Y, Bollas A, Wang Y, Au KF. Nanopore sequencing technology, bioinformatics and applications. Nature biotechnology 2021;39(11):1348–1365.
13. Payne A, Holmes N, Clarke T, Munro R, Debebe BJ, Loose M. Readfish enables targeted nanopore sequencing of gigabase-sized genomes. Nature biotechnology 2021;39(4):442–450.
14. Li H. Minimap2: pairwise alignment for nucleotide sequences. Bioinformatics 2018;34(18):3094–3100.
15. Kovaka S, Fan Y, Ni B, Timp W, Schatz MC. Targeted nanopore sequencing by real-time mapping of raw electrical signal with UNCALLED. Nature biotechnology 2021;39(4):431–441.
16. Zhang H, Li H, Jain C, Cheng H, Au KF, Li H, et al. Real-time mapping of nanopore raw signals. Bioinformatics 2021;37(Supplement_1):i477–i483.
17. Han R, Li Y, Gao X, Wang S. An accurate and rapid continuous wavelet dynamic time warping algorithm for end-to-end mapping in ultra-long nanopore sequencing. Bioinformatics 2018;34(17):i722–i731.
18. Dunn T, Sadasivan H, Wadden J, Goliya K, Chen KY, Blaauw D, et al. SquiggleFilter: An Accelerator for Portable Virus Detection. In: MICRO-54: 54th Annual IEEE/ACM International Symposium on Microarchitecture; 2021. p. 535–549.
19. Senanayake A, Gamaarachchi H, Herath D, Ragel R. DeepSelectNet: deep neural network based selective sequencing for oxford nanopore sequencing. BMC bioinformatics 2023;24(1):31.
20. Bao Y, Wadden J, Erb-Downward JR, Ranjan P, Zhou W, McDonald TL, et al. SquiggleNet: real-time, direct classification of nanopore signals. Genome biology 2021;22:1–16.
21. Firtina C, Ghiasi NM, Lindegger J, Singh G, Cavlak MB, Mao H, et al. RawHash: Enabling Fast and Accurate Real-Time Analysis of Raw Nanopore Signals for Large Genomes. bioRxiv 2023;p. 2023–01.
22. Jain M, Koren S, Miga KH, Quick J, Rand AC, Sasani TA, et al. Nanopore sequencing and assembly of a human genome with ultra-long reads. Nature biotechnology 2018;36(4):338–345.
23. Deamer D, Akeson M, Branton D. Three decades of nanopore sequencing. Nature biotechnology 2016;34(5):518–524.
24. Petersen LM, Martin IW, Moschetti WE, Kershaw CM, Tsongalis GJ. Third-generation sequencing in the clinical laboratory: exploring the advantages and challenges of nanopore sequencing. Journal of clinical microbiology 2019;58(1):e01315–19.
25. Logsdon GA, Vollger MR, Eichler EE. Long-read human genome sequencing and its applications. Nature Reviews Genetics 2020;21(10):597–614.
26. Wick RR, Judd LM, Holt KE. Performance of neural network basecalling tools for Oxford Nanopore sequencing. Genome biology 2019;20(1):1–10.
27. Edwards HS, Krishnakumar R, Sinha A, Bird SW, Patel KD, Bartsch MS. Real-time selective sequencing with RUBRIC: read until with basecall and reference-informed criteria. Scientific reports 2019;9(1):1–11.
28. Ulrich JU, Lutfi A, Rutzen K, Renard BY. ReadBouncer: Precise and Scalable Adaptive Sampling for Nanopore Sequencing. bioRxiv 2022;.
29. Guo L, Lau J, Ruan Z, Wei P, Cong J. Hardware acceleration of long read pairwise overlapping in genome sequencing: A race between fpga and gpu. In: 2019 IEEE 27th Annual International Symposium on Field-Programmable Custom Computing Machines (FCCM) IEEE; 2019. p. 127–135.
30. Liyanage K, Gamaarachchi H, Ragel R, Parameswaran S. Cross Layer Design Using HW/SW Co-Design and HLS to Accelerate Chaining in Genomic Analysis. IEEE Transactions on Computer-Aided Design of Integrated Circuits and Systems 2023;.
31. Kruskal JB. An overview of sequence comparison: Time warps, string edits, and macromolecules. SIAM review 1983;25(2):201–237.
32. Müller M. Dynamic time warping. Information retrieval for music and motion 2007;p. 69–84.
33. Juang BH. On the hidden Markov model and dynamic time warping for speech recognition—A unified view. AT&T Bell Laboratories Technical Journal 1984;63(7):1213–1243.
34. Tuzcu V, Nas S. Dynamic time warping as a novel tool in pattern recognition of ECG changes in heart rhythm disturbances. In: 2005 IEEE international conference on systems, man and cybernetics, vol. 1 IEEE; 2005. p. 182–186.
35. Albanese D, Visintainer R, Merler S, Riccadonna S, Jurman G, Furlanello C. mlpy: Machine learning python. arXiv preprint arXiv:12026548 2012;.
36. Keogh E, Wei L, Xi X, Lee SH, Vlachos M. LB_Keogh supports exact indexing of shapes under rotation invariance with arbitrary representations and distance measures. In: Proceedings of the 32nd international conference on Very large data bases Citeseer; 2006. p. 882–893.
37. Lemire D. Faster retrieval with a two-pass dynamic-time-warping lower bound. Pattern recognition 2009;42(9):2169–2180.
38. Sakoe H, Chiba S. Dynamic programming algorithm optimization for spoken word recognition. IEEE transactions on acoustics, speech, and signal processing 1978;26(1):43–49.
39. Itakura F. Line spectrum representation of linear predictor coefficients of speech signals. The Journal of the Acoustical Society of America 1975;57(S1):S35–S35.

40. Gamaarachchi H, Parameswaran S, Smith MA. Featherweight long read alignment using partitioned reference indexes. Scientific reports 2019;9(1):4318.
41. Gamaarachchi H, Samarakoon H, Jenner SP, Ferguson JM, Amos TG, Hammond JM, et al. Fast nanopore sequencing data analysis with SLOW5. Nature biotechnology 2022;p. 1–4.
42. Li H, Handsaker B, Wysoker A, Fennell T, Ruan J, Homer N, et al. The sequence alignment/map format and SAMtools. Bioinformatics 2009;25(16):2078–2079.
43. Gamaarachchi H, Lam CW, Jayatilaka G, Samarakoon H, Simpson JT, Smith MA, et al. GPU accelerated adaptive banded event alignment for rapid comparative nanopore signal analysis. BMC bioinformatics 2020;21(1):1–13.
44. Simpson JT, Workman RE, Zuzarte P, David M, Dursi L, Timp W. Detecting DNA cytosine methylation using nanopore sequencing. Nature methods 2017;14(4):407–410.
45. Rodriguez-Morales AJ, Gallego V, Escalera-Antezana JP, Méndez CA, Zambrano LI, Franco-Paredes C, et al. COVID-19 in Latin America: The implications of the first confirmed case in Brazil. Travel medicine and infectious disease 2020;35:101613.
46. Sadasivan H, Stiffler D, Tirumala A, Israeli J, Narayanasamy S. Accelerated Dynamic Time Warping on GPU for Selective Nanopore Sequencing. bioRxiv 2023;p. 2023–03.
47. Sadasivan H, Wadden J, Goliya K, Ranjan P, Dickson RP, Blaauw D, et al. Rapid real-time squiggle classification for read until using rawmap. bioRxiv 2022;p. 2022–11.
48. Sneddon A, Ravindran A, Hein N, Shirokikh NE, Eyras E. Real-time biochemical-free targeted sequencing of RNA species with RISER. bioRxiv 2022;p. 2022–11.
49. Sart D, Mueen A, Najjar W, Keogh E, Niennattrakul V. Accelerating dynamic time warping subsequence search with GPUs and FPGAs. In: 2010 IEEE International Conference on Data Mining IEEE; 2010. p. 1001–1006.
50. Wang Z, Huang S, Wang L, Li H, Wang Y, Yang H. Accelerating subsequence similarity search based on dynamic time warping distance with FPGA. In: Proceedings of the ACM/SIGDA international symposium on Field programmable gate arrays; 2013. p. 53–62.
51. Fernandez I, Manglik A, Giannoula C, Quislant R, Ghiasi NM, Gómez-Luna J, et al. Accelerating Time Series Analysis via Processing using Non-Volatile Memories. arXiv preprint arXiv:221104369 2022;.
52. Schmidt B, Hundt C. cuDTW++: Ultra-Fast Dynamic Time Warping on CUDA-Enabled GPUs. In: Euro-Par 2020: Parallel Processing: 26th International Conference on Parallel and Distributed Computing, Warsaw, Poland, August 24–28, 2020, Proceedings 26 Springer; 2020. p. 597–612.
53. Hundt C, Schmidt B, Schömer E. Cuda-accelerated alignment of subsequences in streamed time series data. In: 2014 43rd International Conference on Parallel Processing IEEE; 2014. p. 10–19.

Click here to access/download
Supplementary Material
Supplementary Information_HARU.pdf

Reviewer Response

Click here to access/download
Supplementary Material
HARU_reviewer_response.pdf

Personal Cover

Click here to access/download;Personal Cover;CoverLetter.pdf

384 Victoria Street,
Darlinghurst, Sydney
NSW 2010, Australia

t +61 2 9295 8100
f +61 2 9295 8136
www.garvan.org.au

Garvan Institute
of Medical Research

REVISED MANUSCRIPT SUBMISSION – ***Efficient Real-Time Selective Genome Sequencing on Resource-Constrained Devices***

Dear Editor,

We thank you for giving us the opportunity to submit a revised manuscript. We have addressed the issues raised by the reviewers or have provided reasons in the point-by-point response (attached to the end of this letter). Reviewers 1,2 and 4 were large positive about the innovation, novelty and contribution in the system-level design. However, reviewer 3 seemed dissatisfied with the novelty aspect and may have overlooked that this is a technical note rather than a research article.

The editorial comments have been addressed as follows:

Overall, the reviewers are supportive of publication. One major concern mentioned by several reviewers is that the evaluation and comparisons to similar approaches need to be improved, also to highlight differences and improvements compared to existing work.

Initially, we did not provide comparisons with other methods because none of them was designed to be executed on a portable, low-cost and resource-constrained device like the one HARU runs on, which has only 4-ARM cores, 4GB RAM, and consumes less than 3W of power. Nonetheless, to address the reviewers comments in this revised manuscript, we executed those other methods (DeepSelectNet, Minimap2+Guppy, etc) on high-end devices and compared them to HARU executed on a low-end embedded system. Although the difference in execution platform makes the comparison biased in favour of other methods, our results show that HARU still has better performance.

Reviewer #2 mentions that "the source code for both implementations of the optimized RUscripts in C and Python is not open nor available". Please note it is a requirement for publication in GigaScience that all supporting code is available under an OSI-approved open licence.

The links to these source codes were already provided as open-source under “Availability of source code and requirements” section. Now we have also added the links to all repositories as key-points in the manuscript so those links may not be accidentally overlooked by a reader.

On a minor note, please also structure your abstract ("Background", "Findings", "Conclusions").

We have structured the abstract as per your suggestion.

We have also attached the responses to the reviewers as the back of this letter.

Sincerely,

Dr Hasindu Gamaarachchi
Genomics Computing Research Scientist | Genomic Technologies Group | Kinghorn Centre for Clinical Genomics
a: 384 Victoria Street, Darlinghurst NSW 2010
p: +612 9355 5883
e: hasindu@garvan.org.au

384 Victoria Street,
Darlinghurst, Sydney
NSW 2010, Australia

t +61 2 9295 8100
f +61 2 9295 8136
www.garvan.org.au

Garvan Institute of Medical Research

Reviewer #1:

This submission is a solid application work to accelerate low-latency, signal-level genome alignment on FPGAs. It provides an end-to-end design for portable sequencers to analyze input genome and discard unnecessary reads in real-time. By providing an open-source RTL design with an integration example, the work brings 2.5× performance with impressive energy efficiency to the community. I would expect this work could facilitate future research.

This paper is well-written, with an extensive explanation of the background. I found it entertaining to read, even with little background knowledge of signal-level processing of genomes. The research goal of low-cost targets and low-latency processing are well motivated. The experimental results are solid and extensive, with an optimized baseline for comparison and end-to-end performance analysis, including overhead time. The authors combine knowledge from different fields and co-optimize the design, for example, the data scaling with acceptable accuracy reduction.

We thank the reviewer for these encouraging comments.

Q1. Although well-written, my main concern about this work is that it seems incremental and needs to exhibit more research innovation. The authors successfully combine multiple optimizations, but most optimization approaches are well-known. For example, the 2D dynamic programming (DP) algorithm without backtrace requirement is well-studied for not only FPGA accelerations but also from the space reduction perspective. Pipelining and data reusing are both standard practices in the FPGA community. I would appreciate it if the authors could highlight the improvement and difference from the regular 2D DP problem and elaborate more on the novelty of their work at the system integration level.

We appreciate the reviewer's feedback and agree that the initial manuscript did not emphasize the novelty of the system-level integration work sufficiently. Our system-level integration of our end-to-end overarching architecture involves several novel components, such as optimized squiggle preparation for normalization using multithreading, the custom driver for high-throughput transferring via the AMBA AXI4-Stream, and a portable and efficient hardware accelerator. We can also use Kria's xmutil utility tool to swap bitstreams without needing to reboot the system, which makes it easy to adapt to changes or customize towards applications if needed. The hardware control interface uses AXI-Lite, while data streaming (query and reference) uses AXI stream, which enables high throughput on low-cost MPSoC platforms.

We have updated the introduction of the manuscript to highlight the novelty of our system-level integration (page 2).

Q1.2. Another concern is that this work needs to discuss its related work sufficiently. Although there are a few paragraphs on the acceleration of signal alignment, only a few base-alignment approaches are referred to. Although different in the format and semantics of the input data, the algorithm is similar, and I believe the authors could reuse many of the existing accelerator designs. Besides, the discussion on Minimap2 on page 2 is not accurate, as there is at least one published FPGA acceleration (Hardware Acceleration of Long Read Pairwise Overlapping in Genome Sequencing: A Race Between FPGA and GPU), along with GPU implementations. This inaccuracy also undermines the motivation of processing at the signal level. I would appreciate it if the authors further discussed the reason for using a signal-based approach.

Thank you for your feedback on related work. Currently, when performing base-level alignment-based selective sequencing, the bottleneck is basecalling and not the alignment (Minimap2). CPU-based Minimap2 is still very fast and takes only 2-5% of the time in a base-level alignment-based selective sequencing workflow. 95-98% of the time is spent on basecalling, despite using GPU (fast basecalling model). Therefore, those accelerations on Minimap2 are less relevant unless the basecalling in significantly accelerated. We have clarified on page 2 of our report that Minimap2 is not the bottleneck and that the basecalling process is the cause of the bottleneck.

We have also discussed recent acceleration on Minimap2 on page 3.

In fact, the observation that alignment in the base-space is very fast compared to basecalling is what makes signal alignment a potentially better method. We have clarified this on page 3.

384 Victoria Street,
Darlinghurst, Sydney
NSW 2010, Australia

t +61 2 9295 8100
f +61 2 9295 8136
www.garvan.org.au

Garvan Institute of Medical Research

Q1.3. There are a few minor comments I would appreciate if the authors could address before publication:

(1.3.1) It seems that as M is implemented as the pipeline depth, it is set to a constant. Does M changes as the data changes? Or does the detection accuracy change if M does not change with the data?

We conducted experiments investigating the impact of query length (denoted by M) on mapping accuracy and found that accuracy increased with longer query lengths. However, trimming the prefix sequence (which belongs to the adaptor and barcode, if present) proved more crucial for maintaining high accuracy. This prefix is pre-determinable and is more desired than having a varying query length. A query length of 250 was chosen to balance speed and accuracy. If for other reasons a different query length is needed our hardware can be updated to accommodate varying query lengths, allowing users to dynamically choose the appropriate bitstream using the Kria platform's xmutil tool. Longer query lengths, though, may require increased squiggle collection wait time and risk pore damage from clogging. We have discussed this in Supplementary Note 5: "Selection of query length" associated with the updated manuscript.

1.3.2 Does the data scaling method work for all data? How about using dynamic scaling instead of a set scale?

The data scaling method we used involves converting the 13-bit MinION integer data and 11-bit PromethION integer data to real numbers using picoampere conversion and z-score normalization. These real numbers are then represented and processed in fixed-point representation with a scaling factor of 32 (5 fractional bits) on the HARU hardware to achieve resource efficiency.

Does the data scaling method work for all data?
We found that the normalized data typically follow a Gaussian distribution, and after normalization, only a very small fraction of samples (0.0027%) have a magnitude greater than 3. We accumulated such values over 512 samples, so the probability of the magnitude exceeding 1024 is negligible. Based on this, we determined that using 16 bits (11 integer bits and 5 fractional bits) is sufficient to represent all types of data without significant loss of precision.

We also performed experiments to empirically validate this method for multiple target genomes, including SARS-CoV-2, Ebola, Ecoli, and Lambda datasets publicly available. Supplementary Note 1, associated with the revised mansuscript, now discusses these experiments and the mapping accuracy percentage difference between sigfish using 32-bit floating points and sigfish-HARU which uses 16-bit fixed-point with scaling method. The accuracy of each version of sigfish measured against the results obtained from Minimap2 (if the mapping coordinate is within 200 bases proximity they are considered correct) only differed by 0-0.05% for all the genomes tested.

How about using dynamic scaling instead of a set scale?
Dynamic scaling can be useful when the dynamic range of the input data is not known in advance, as it allows for the scaling factor to be adjusted on-the-fly to accommodate varying input magnitudes. However, in the case of our sDTW algorithm, the input data comes from a well-defined and consistent range, as explained earlier, making a static scaling factor a more appropriate and efficient choice for hardware implementation. Additionally, fixed-point arithmetic with a set scaling factor can often be faster and more power-efficient than using dynamic scaling or floating-point arithmetic, which requires more complex hardware and consumes more power as discussed in Supplementary Note 6.

1.3.3 In Figure 7, "25" should be "256".

We have updated the manuscript.

Nevertheless, this work is publishable, and the audiences will benefit from its results.

We again thank the reviewer for the positive and constructive feedback that allowed us to improve the quality of the manuscript.

---

384 Victoria Street,
Darlinghurst, Sydney
NSW 2010, Australia

t +61 2 9295 8100
f +61 2 9295 8136
www.garvan.org.au

Garvan Institute of Medical Research

Reviewer #2:

The authors observe that existing Read Until approaches that employ subsequence Dynamic Time Warping (sDTW) algorithm are computationally intensive such that a capable workstation with dozens of CPU cores struggles to keep up with the data rate of a mobile phone-sized MinION sequencer.

The authors present Hardware Accelerated Read Until (HARU), a resource-efficient hardware-software co-design-based method that exploits a low-cost and portable heterogeneous Multiprocessor System-on-Chip (MPSoC) platform with on-chip Field-Programmable Gate Arrays (FPGA) to accelerate the sDTW-based Read Until algorithm. Experimental results show that HARU on a Xilinx FPGA embedded with a 4-core ARM processor is around 2.5× faster than a highly optimized multi-threaded software version (around 85× faster than the existing unoptimized multi-threaded software) running on a sophisticated server with 36-core Intel Xeon processor for a SARS-CoV-2 dataset.

I like this work. The paper appears to provide significant research contributions. It targets tackling an important problem in the genome sequencing pipeline. The following points need to be addressed.

We thank the reviewers for these positive comments.

Q2.1 The authors make several optimization techniques that I appreciate and agree with. However, the effect of each of these techniques on the execution time, FPGA resource allocation, and more importantly the accuracy of Read Until decisions are not provided.

We thank the reviewer for the suggestion to include more details about the effect of our optimization techniques on execution time, FPGA resource allocation, and accuracy of Read Until decisions.

Accuracy:

The pipelining optimization does not change how sDTW is computed and thus does not affect the accuracy. For operations to be pipelined, each operation can only start after its dependent data are ready, and all computations are done exactly the same as non-pipelined computations. Thus, accuracy is not affected by pipelining. We have now briefly stated this in the subsection "Operation pipelining" (page 8) of the updated manuscript.

The cost matrix memory optimization is applicable in the selective sequencing application because we only need either the start or end of the mapping of squiggles in the reference, which means backtracking is not needed as long as we keep track of the minimum value in the last row of the cost matrix. The cost matrix memory optimization still returns the same end position of the mapping as is without the optimization. We have briefly stated this in the subsection "Cost matrix memory optimization" (page 8) of the updated manuscript.

However, we also acknowledge that using fixed-point representation may result in some loss of precision, and we already explored this in detail in the subsection "Fixed-point data representation" of the original manuscript. In addition, in the supplementary Note 1 of the updated manuscript, we have included experimental results that the accuracy of the fixed point representation is nearly identical to using floating point (see the response in reviewer#1's question)

FPGA resource allocation and Execution time

Different optimizations can interact with one another, potentially resulting in varying execution times and resource allocations when different combinations are applied. As such accurately measuring the effect due to individual optimizations poses a significant difficulty. Therefore, we believe that, ultimately, it's the final execution time and the FPGA resource allocated with all optimizations in place that matter most which we had reported and discussed extensively in the original manuscript. Nonetheless, we have further added Supplementary Note 6 to highlight the impact of different optimizations as best as we can.

Q2.2 I would like to understand more about the effect of choosing 250 events to decide on Read Until. Is sampling more than 250 events needed? The length of read is also an important factor as the length of the ONT reads varies from tens to a few million bases.

384 Victoria Street,
Darlinghurst, Sydney
NSW 2010, Australia

t +61 2 9295 8100
f +61 2 9295 8136
www.garvan.org.au

Garvan Institute
of Medical Research

We have now discussed this in Supplementary Note 5: "Selection of query length" associated with the updated manuscript. The same question was also raised by reviewer 1. The reviewer is requested to kindly refer to the response to Q1.3.1 - query made by reviewer 1 on M being implemented as the pipeline depth.

Q2.3 The source code for both implementations of the optimized RUscripts in C and Python is not open nor available. I would highly encourage the authors to make them available on github.

We apologize for any confusion caused. The links to these source codes were already provided as open-source under "Availability of source code and requirements" in the original manuscript. The Optimised RUScripts implementation in C is in the same repository as sigfish-haru which can be specified as an option to make:

```
# Building sigfish without hardware acceleration
make PROCESSOR=aarch64
# Building sigfish with hardware acceleration
make fpga=1 PROCESSOR=aarch64
```

To avoid such confusion, we added the following as a key point under the manuscript (page 2).

Q2.4 The evaluation of how HARU scales with a different number of threads is not provided. Both the front software interface for HARU and the RUscripts are multithreaded, but the number of CPU threads that makes RUscripts faster than HARU needs to be examined.

The experiments in our paper were conducted with RUScripts executed with **all threads available on the system, which included 36 cores when executing on the HPC**. Although RUScripts was originally written in Python and was not optimized for performance or multithreading efficiency, we re-implemented it in C and optimized it for performance and multithreaded efficiency to provide a fair comparison with HARU. The runtime for the optimized multithreaded implementation of RUScripts was also provided in the paper. As stated in the paper, our HARU system on the edge computing board (costing around $300) is ~85.8× faster than the original RUscripts running with 36 cores on a server and still ~2.49× faster than the 36-core server (costing around $30,000). In the following extract from the original manuscript (now page 4 of the updated manuscript), the fact that all threads were used was stated:

Q2.5 I wonder if the authors also evaluate the benefits of using HARU for base-domain Read Until. The basecalling step contributes significantly to the read accuracy and performing operations in the signal space is still challenging. This is true for most of the Read Until applications. Showing the pros and cons through evaluation of discussion for using HARU for signal domain Read Until versus base domain Read Until can be very useful.

We agree with the reviewer's suggestion that evaluating the benefits of using HARU for base-domain Read Until would be useful. We acknowledge that basecalling is a challenging step in nanopore sequencing and that performing operations in the signal space is still a developing field. However, we believe that signal processing holds great potential for nanopore adaptive sampling on-chip. While piggybacking on existing sequence alignment techniques is easier, signal processing advancements in other fields such as image, video, and audio signal processing have led to the development of miniaturized devices with comparable data rates to nanopore sensors. We have now discussed the pros and cons of each approach under the discussion on page 9 and 10 of the updated manuscript.

Q2.6 For Figure 4, the throughput of the target sequencing machine needs to be added to have a clear comparison with the current need for acceleration.

The relationship between the throughput of the target sequencing machine and the throughput required by the accelerator for effective selective sequencing is very complex. The effectiveness of selective sequencing is determined by whether the reject or sequence decision can be made before the nanopore channel in the sequencer finishes sequencing the current squiggle. The average length of squiggles in nanopore sequencing applications may vary across different targets and can be affected by sample read length distribution. There are also other factors involved in the effectiveness of selective sequencing, including the proportion of on-target and off-target reads in the sample, pore-clogging when a rejection signal is passed back when the

384 Victoria Street,
Darlinghurst, Sydney
NSW 2010, Australia
t +61 2 9295 8100
f +61 2 9295 8136
www.garvan.org.au

nanopore channel has sequenced a majority of the squiggle, software complexities and limitations introduced by MinKNOW (0.4s is the minimum wait time before any data is available). Thus, it is unrealistic to provide any sequencer throughput quantization and discuss its relation with selective sequencing processing throughput to determine Read Until effectiveness.

Q2.7 The authors mention that "Our implementation of HARU loads raw signal from BLOW5 file format because the slow5lib library is lightweight". As the default file format provided by ONT devices is FAST5, I wonder if the execution for format conversion is included in the total execution time.

To clarify, by "lightweight," we meant avoiding the use of bulky libraries. The conversion time is not included because direct loading from RAM through MinKNOW API would eliminate such conversion. However, as the MinKNOW is closed-source and only available in limited settings, we opted to use BLOW5 for this proof-of-concept implementation. It's important to note that BLOW5 is also lightweight in terms of runtime, as demonstrated in https://www.nature.com/articles/s41587-021-01147-4. However, the default file format is controlled by the company, which presents a separate issue.

Q2.8 The reference genome needs to be first loaded to FPGA's block RAM. What if the reference genome doesn't fit into the block RAM? What is the reference genome size that is used in practice for Read Until applications?

In our proof-of-concept implementation, we used block RAMs to load the reference genome, but this is not the only approach. A more flexible way to handle reference genomes is to stream them together with the query signal during runtime, allowing for arbitrarily long references. We have implemented this approach in a separate branch of our code (https://github.com/beebdev/HARU/tree/dynamic-reference), and it allows the accelerator to compute the mappings on the fly while the reference is being streamed into the accelerator.

While the reference streaming branch doesn't limit the reference size, in practice, sDTW-based methods still will not scale for giga-base references. However, we plan to integrate methods such as sigmap and UNCALLED to first perform fast mapping using seeds, followed by running sDTW on potential matches to refine the results. The sDTW mapping operation will involve a much smaller mapping reference size than the full target reference. Currently, these index/seeding-based methods have limitations and require optimizations before they can be fully integrated into our system. We have briefly discussed this under the discussion on page 10 of the updated manuscript.

Q2.9 I understand that the use of low-cost MPSoC is to run the software interface and the hardware accelerator on the same portable device. However, is it possible to use high-end FPGA devices with HBM memories? Is it needed to cope with the throughput of more capable sequencing machines such as PromethION?

While our current implementation targets low-cost MPSoCs, scaling up the hardware implementation to parallelize more PE chain accelerators to handle higher throughputs from more capable sequencing machines such as PromethION is certainly a possibility. Our current design of the accelerator only supports an AXI-stream and AXI-Lite interface, which means that platforms with sufficient resources can parallelize multiple accelerators using multi-channel AXI DMAs. However, efficiently streaming data to a large number of parallel accelerators to achieve the necessary throughput will require faster data buses such as PCIe. Although we have not explored the use of HBM memories or higher-end FPGAs at this point, we appreciate your suggestion for future work and have discussed this possibility under the discussion on page 10 of the updated manuscript.

Q2.10 The command lines for evaluating the existing tools are missing.

We thank the reviewer for reminding this which was overlooked by us. We have added this under Supplementary Note 7.

Q2.11 A few typos:
*** page 3, on the left down side, range of i and j have typo.**
*** On Algorithm 1, first for loop has typo i and j**

Thanks. We updated the manuscript and the algorithm.

---

384 Victoria Street,
Darlinghurst, Sydney
NSW 2010, Australia

t +61 2 9295 8100
f +61 2 9295 8136
www.garvan.org.au

Garvan Institute of Medical Research

Reviewer #3:
This paper proposes a hardware-assisted subsequence Dynamic Time Warping solver for selective genome sequencing from raw signal traces from hand-held nanopore sequencers. To improve the efficiency of ReadUntil framework, the proposed framework introduces a systolic array DTW accelerator deployed on an FPGA based SoC, which is integrated into and driven from a host and driver software. Avoiding implementing the full cost matrix allows their design to scale. Improved sDTW processing has achieved large speedup and energy efficiency over a server system with Xeon CPUs.

+ The authors have performed a system-wide study with a full-stack proof-of-concept.
+ Compact PE design that allows it to be implemented on a small FPGA or an edge device.

In this paper, the authors propose a hardware software co-design for selective genome sequencing using sDTW deployed on FPGA. This paper is well written and easy to read. Notably, one of the strengths of this paper is that they demonstrated the full stack implementation of the proposed FPGA solution, and the hardware and software stacks are open-sourced. I guess non-trivial implementation and verification efforts have been made, and I think this can be one of the reasons for this paper to be accepted.

We thank the reviewer for this positive feedback.

Q3.1 Incremental work to SquiggleFilter [37]. The architecture itself is a deployment of a well-known systolic array architecture for dynamic programming including DTW and Smith-Waterman.

The main focus of HARU is the end-to-end integrated system architecture and it is not limited to the sDTW core. We agree that our sDTW core is built upon well known optimizations in hardware (so does the SquiggleFilter) and the primary innovation lies in the overarching system architecture as appreciated by reviewer 1 and 2.

The sDTW core in HARU started as an undergraduate honours thesis project in 2020 and the design was complete by mid 2021, during which the SquiggleFilter publication did not exist. The designs happened to be similar despite being developed independently, because well-known optimizations have been used in both designs. However, as our sDTW core itself was not a complete system and we realised that there is limited utility of such a hypothetical design, we pursued to develop an end-to-end system closer to integration with a sequencer. In fact, the overall system integration turned out to be a more challenging problem than designing the sDTW core itself. We re-iterated our system for overall performance and robustness, leading to the architecture we presented in our paper. While our architecture is based on well-known systolic array designs for dynamic programming, its novelty and practical usefulness comes from its application-oriented end-to-end system-level design and integration with the broader bioinformatics ecosystem.

As suggested by reviewer 1, we have elaborated on the system design more deeply in the introduction of the updated manuscript.

Q3.2 The design is only compared with a single type of target genome (SARS-CoV-2), and the sensitivity to the sample property is not explored deeply.

The computational method of using event detection followed by sDTW for alignment was originally shown in the RUScripts paper by Loose et al, which has already demonstrated its effectiveness through a series of experiments. HARU is built on top of this Loose et al’s work, as acknowledged in the original manuscript. Thus, we believe that repeating multiple such experiments is redundant.

The only optimization in HARU that can cause a deviation from the original sDTW algorithm used in RUscripts is the scaling optimization, all other optimizations guarantee the same answer as sDTW used in Loose et al’s work (see the response to Q2.1). To evaluate the impact of this scaling optimization, we have now performed more experiments using different samples such as Ebola, Ecoli, and yeast in the supplementary note. We found out that the accuracy mapping location reported by HARU differs from the accuracy from the original sDTW by only 0-0.05% (see Supplementary Note 1).

384 Victoria Street,
Darlinghurst, Sydney
NSW 2010, Australia

t +61 2 9295 8100
f +61 2 9295 8136
www.garvan.org.au

Garvan Institute
of Medical Research

In terms of “the sensitivity to the sample property”, please refer to the Supplementary Not), to see how the sDTW scores can be used to discriminate between two samples.

Q3.3 Comparisons with the state-of-the-art are not provided.

We have added comparisons to Supplementary Note 4 and a brief summary to section *Results* (page 6) and *Methods* (Page 9) of the revised manuscript. Please also refer to the response for the related Q3.9 for comparisons between HARU and SquiggleFilter, DeepSelectNet, UNCALLED, Guppy + Minimap2.

Q3.4 The motivation for this paper lies in the fact that direct read mapping (or adaptive sampling) using Read Until cannot keep up with the sequencing throughput without a full-fledged GPU. While direct mapping using the raw current signals in fast5 admittedly has heavy computation loads, it is not clear to me why direct mapping from squiggles is a good option in the first place. MinION's fast basecalling can be done with an edge GPU or mobile devices (e.g., Mk1C can do fast realtime basecalling using its embedded GPU, and Mk1D is announced to work with iPad Pro's M1 processor and neural engines), and aligning basecalled reads looks much efficient as it needs to deal with less data (fastq is a lot smaller (1/5 ~ 1/10 in size), compared to fast5). While they try to "revitalize the direct signal approach," the motivation and the goal of this work, especially why it should start from the raw current signals, do not seem to be clear. Also, the accuracy is only compared with
RUscripts with sDTW but not with a pipeline with basecalling that many MinION practices follow.

We acknowledge that basecalling from squiggles is a popular and effective approach for many MinION applications, and we did not intend to suggest that it should be replaced. Rather, our goal was to investigate the feasibility and potential benefits of direct signal mapping, which has been previously explored and is an ongoing research area. We believe that direct mapping from squiggles can provide a more complete and accurate representation of the underlying signal, which may be particularly useful for applications that require higher sensitivity or resolution than current basecalling methods can achieve. This is more of a philosophical discussion and we have added a section called “Signal-level vs base-level selective sequencing” on page 9 of the discussion of the updated manuscript.

Reading raw-signals has no such heavy computational bottlenecks if efficient formats such as BLOW5 [https://www.nature.com/articles/s41587-021-01147-4] are used, the problem is in FAST5 rather than with the raw signal. If the raw signal is directly loading from RAM through MinKNOW API, that would anyway eliminate such loads. However, as the MinKNOW is closed-source and only available in limited settings, we opted to use BLOW5 for this proof-of-concept implementation.

Mk1C can do some limited fast realtime basecalling, however, the best-case throughput mentioned on the manufacture’s website cannot be not achieved in practice when we tested. When using Mk1C for selective sequencing, however, enrichment observed is very limited. This is why we currently use Mk1B connected to a workstation with a 3090 GPU for our other in-house selective sequencing experiments that perform redfish. While the Mk1D is announced, it doesn’t yet exist and thus we cannot reach any conclusions.

Regarding the comparison with existing methods, we agree that it would be useful to evaluate the performance of our approach against a pipeline that includes basecalling which we have addressed in the response to Q3.9.

Q3.5 Fundamentally, the sDTW-based approach lacks the seeding step which is critical in the general sequence alignment to reduce search space in the reference genome, so every time all possible positions in the reference need to be searched, and the applicability of this approach looks limited.

We agree that the seeding step is a common technique used in the sequence alignment to reduce the search space in the reference genome, and it can be especially helpful when dealing with long-read sequences. As mentioned in the discussion of our original manuscript, other tools such as sigmap and UNCALLED have already implemented a seeding step and indexing and these tools are continually evolving. Our work with HARU is intended to complement these tools by being used as an accurate

384 Victoria Street,
Darlinghurst, Sydney
NSW 2010, Australia
t +61 2 9295 8100
f +61 2 9295 8136
www.garvan.org.au

refinement step after approximate search methods such as chaining is performed. In fact, general sequence alignment tools such as Minimap2 perform a variant of Smith-Waterman called Suzuki's formulation on potential mapping locations found by chaining.

Q3.6 SquiggleFilter [37] has introduced a very similar approach for raw signal (squiggle) filtering, using a wide sDTW systolic array on ASIC (their work has also verified with FPGA), in order to quickly find viral genomes. The main difference between SquiggleFilter and this work lies in the input granularity: SquiggleFilter uses 2000 sample points while this work uses 250 events, where each sample point or event needs one PE. I think the authors' approach of using a small number of systolic PEs makes sense to reduce resource amount, however, similar systolic array approaches using the partial diagonal PE band for dynamic programming algorithms such as sDTW and Smith Waterman have been well-known from decades ago [ref1, ref2], and I personally did not find their hardware design has much new insights or novelty compared to the prior works. I believe integrating them into one system with a host software for sure requires non-trivial engineering work, but as an academic paper I think this work is a bit too incremental.

In addition to our response for Q3.1 about the squiggleFilter and novelty, we also like to remind that this article is submitted as a technical note rather than a research article.

Regarding the SquiggleFilter being verified on FPGA, it seems that the authors of SquiggleFilter, despite stating the need for 2000 PEs for 2000 sample points, used only 2 PEs to synthesise for the FPGA implementation results (and only synthesised on AWS instance) while using 100 PEs for behavioural simulation (functional verification). Furthermore, the source code they provided does not contain a synthesizable top-level module that collectively connects all submodules. We opened a GitHub issue on these topics and have received confirmation that the synthesis verification for FPGA was done individually for each submodule (as they do not have the full system and only envision it) and the number of PEs used for synthesis was indeed done on a smaller number that is not 2000 (see https://github.com/TimD1/SquiggleFilter/issues/1). In addition, SquiggleFilter is an ASIC design that is expensive to manufacture and integrate with a full SoC and is vulnerable to selective sequencing requirement changes, while the effort needed to update HARU is much easier. As mentioned in the manuscript, HARU is a low-cost and highly efficient end-to-end Read Until solution that runs on off-the-shelf low-cost FPGA SoCs, which we believe will highly benefit the ongoing improvements in signal-level alignment research in genomic sequencing. Overall, SquiggleFilter does not provide a fully integrated working system (as re-stated by the author in the issue linked above) which is the main difference between HARU and SquiggleFilter. Please refer to the response for Q3.9 for comparison with SquiggleFilter.

Q3.7 Recent work, SquiggleNet [ref3], also proposes a similar pre-filtering approach of raw squiggles using a machine learning model. This model is very lightweight and shown to have good accuracy. It also needs a GPU for fast filtering, but because it is based on the well-known ResNet model, it can be easily accelerated by an accelerator such as an edge TPU. I wonder how the sDTW-based approach compares with such ML-based ones.

While neural network-based methods have some advantages, they also have their own limitations. One significant issue is that they require training for every new dataset, which can be time-consuming and computationally expensive. Moreover, they may not be suitable for applications that require per-position mapping or depletion/enrichment analysis, as they typically require both positive and negative training data from both samples.

Regarding the use of edge TPUs to accelerate SquiggleNet, please note that typical edge TPUs are designed to support int8 types for energy- and power efficiency (e.g., https://cloud.google.com/edge-tpu), while SquiggleNet reports experimentation and implementation on workstation GPUs and does not mention the use of int8. While it is possible to convert the model to int8, it will require additional research and optimization effort, and it is unclear how it would affect the accuracy and efficiency of the model. Thus, using edge TPUs for NN-based selective sequencing is yet a hypothesis.

As for the comparison with neural network-based methods, see the response for Q3.9 that compares with state-of-the-art methods, where we compare HARU with DeepSelectNet, which is a work recently published that optimizes SquiggleNet. Comparing throughput results, HARU exceeds the throughput of DeepSelectNet and the accuracy is slightly better, despite the fact that DeepSelectNet was ran on a HPC system with a high-end Tesla V100 GPU, whereas the HARU runs on a low-power, resource constrained device.

384 Victoria Street,
Darlinghurst, Sydney
NSW 2010, Australia

t +61 2 9295 8100
f +61 2 9295 8136
www.garvan.org.au

Garvan Institute of Medical Research

Q3.8 The evaluation can be improved to have more depth. They only compared a single type of viral genome (SARS-CoV-2) and a narrow region of a human genome. The sensitivity or robustness to the property of input genome, such as repetitions, variants, mutations, and read length, and to the diversity of the mixes of the genome being sequenced (e.g. respiratory metagenome has about 1000:1 human to viral genome ratio) are not explored.

Please see the response to Q3.2 on experiments using different input genomes. Also, see Q3.12 for the effect of mutations. For experiments on the effect of the query length, please refer to the response to Q1.3 - query made by reviewer 1 on M being implemented as the pipeline depth and also Supplementary Note 5.

Q3.9 The comparisons have been made based on the RUscripts (and sDTW), but it might be a bit narrow-scoped. It would be helpful if authors can include quantitative comparisons with the state-of-the-art work including SquiggleFilter and SquiggleNet, and other approaches not based on RUscripts or sDTW (like Minimap2 or UNCALLED). I would also make this paper insightful if the authors can provide a discussion on signal alignment and base alignment.

They reason why we did not provide comparisons was because none of the existing methods could execute on a resource-constrained device like the one HARU runs on, which has only 4-ARM cores, 4GB RAM, and consumes less than 10W of power (power analysis from post-implementations reports 2.941W for the whole system. See Supplementary Note 8). However, in this revised manuscript, we executed those other methods on high-end devices where possible and compared them to HARU executed on a low-end embedded system. Although the difference in execution platform makes the comparison biased in favour of other methods, our results show that HARU still has better performance.

We have added those comparisons to Supplementary Note 4 and a brief summary to section *Results* (page 6) and *Methods* (Page 9) of the manuscript.

Q3.10 The authors claim up to 5 parallel query processors can be supported by the tested board, but given the LUT utilization for a single processor, I doubt if 5 instances can actually be placed & routed.

We thank the reviewer for pointing this out. The doubt regarding whether 5 instances of accelerators can be placed and routed for the target device is valid. To verify the ability to fit multiple accelerators in parallel, we have now included a separate branch (https://github.com/beebdev/HARU/tree/dynamic-reference) that allows for streaming the query and reference signals together, which eliminates the need for storing the reference signal in the FPGA's block RAM. This makes each accelerator independent of each other and does not contain critical paths between the accelerators. Through synthesis and implementation, we see that the most used resource for a single instance of the accelerator is the CLB LUT, taking up to 22% of availability post-synthesis (see Supplementary Note 2, Supplementary Table 1). With this information, we connected four accelerators with a multi-channel AXI DMA and ran synthesis and implementation. Post-implementation resource utilization is shown in Supplementary Table 2 of Supplementary Note 2 and all timing constraints were satisfied. Based on the results we agree that having five accelerators in parallel might be a little bit too tight and require some effort to fit and we have updated the manuscript under "Resource utilization" to change the claim from 5 to 4.

Q3.11 What observation has led you to conclude that "250 events are adequate for mapping"?

See the response to Reviewer#1's question Q1.3.1 on this topic and also the newly added Supplementary Note 5.

Q3.12 What would you do if the input reads need more than 250 events (e.g. targeting only a specific variant of a virus) to do the selective read alignment?

Targeting a specific variant does not necessarily require more events. In selective sequencing, we take the beginning of the read and not the whole read. The SARS-CoV-2 reference we used was MN908947.3 [https://www.ncbi.nlm.nih.gov/nuccore/MN908947], which is the original Wuhan lineage B. The SARS-CoV-2 SP1 data set we

384 Victoria Street,
Darlinghurst, Sydney
NSW 2010, Australia

t +61 2 9295 8100
f +61 2 9295 8136
www.garvan.org.au

Garvan Institute of Medical Research

used is B.1 lineage that contained several variants from the reference, however, sDTW could map with >95% accuracy (see Supplementary Note 4). We additionally tested on a sequencing run containing 10 barcoded SARS-CoV-2 samples (a mix of A2.2, B.1 and B.28 lineages) and sDTW could map with >95% accuracy (see Supplementary Note 3). In fact, it is not the query size used for sDTW that matters, but it is the length of the prefix trimmed that is important to properly eliminate the adaptor (and barcode if present) which can be deduced using information available at library preparation (see Supplementary Note 5).

Having said they, if there a specific reasons that require the query length to be larger than 250, users can compile and synthesize HARU with their desired query length and load the system using the xmutil tool on the Kria platform (will consume more area and can be increased up to 1000 events in theory for the Kria FPGA board). However, if we increase it, the accuracy of mapping will increase at cost of adaptive sampling efficiently (number of bases sequenced from unwanted regions; for example rejecting unwanted reads at 250 events at 87% accuracy vs 500 events at 95% accuracy). Also, note that rejecting reads after too many bases have passed through the pore can be detrimental, as long strands passed through the pore when rejected can get clogged and destroy the pore.

Q3.13 Any quantification of the scalability?

For throughput scalability, our hardware architecture is designed with consideration for synthesizing and implementing multiple accelerator instances on the FPGA fabric, which can be streamed with the ARM processor on the MPSoC through the multichannel AXI DMA device as long as the overall resource utilization is below the available resources (approximately 4 on the Kria board). With more parallel instances of the accelerator running on the MPSoC device, we can achieve higher throughput. Additionally, our software implementation uses multithreading for the normalization, and with Kria having quad-core, we can have 4 threads each owning one HARU accelerator. Moreover, since the MPSoC devices are edge platforms, we can deploy clusters of the MPSoC to provide more devices.

Regarding target reference length scalability, we acknowledge that the performance of DTW-based implementations do not scale well with target reference lengths. However, if our work is combined with existing seeding/indexing methods in sigmap or UNCALLED it could be possible to first possible locations on large references through index searching and then use sDTW for refinement. While sDTW remains linear complexity against the reference length, our hardware design and software implementation provide significant acceleration in the search process.

In summary, our work provides insights into both throughput scalability and target reference length scalability through our hardware design and software implementation. While there are limitations to the scalability of DTW-based implementations, we believe that our work provides significant acceleration in the search process, and we are constantly exploring new ways to improve scalability in our future work.

---

384 Victoria Street,
Darlinghurst, Sydney
NSW 2010, Australia

t +61 2 9295 8100
f +61 2 9295 8136
www.garvan.org.au

Garvan Institute
of Medical Research

Reviewer #4:

Subsequence Dynamic Time Warping (sDTW) is an important algorithm for processing signals arising from third-generation genomic sequencers. In particular, certain applications with real-time requirements can benefit from efficient computational platforms for this task. The paper adresses this need by presenting a new FPGA-based solution called HARU. It is demonstrated that the approach is energy-efficient and can outperform a self-written C implementation. In addition, HARU is made publicly available which can be beneficial to the bioinformatics community.

We thank the reviewer for the feedback.

A weakness of the paper is the limited comparison to the state-of-the-art in the DTW and sDTW area. There are several newer GPU-based and NVM-based approaches that should be considered, such as
- Fernandez, et al. "Accelerating Time Series Analysis via Processing using Non-Volatile Memories." arXiv preprint arXiv:2211.04369 (2022).
- Schmidt, et al. "cuDTW++: Ultra-Fast Dynamic Time Warping on CUDA-Enabled GPUs." European Conference on Parallel Processing. Springer, Cham, 2020.
- Hundt, et al. "Cuda-accelerated alignment of subsequences in streamed time series data." 2014 43rd International Conference on Parallel Processing. IEEE, 2014.

In particular, I suggest using a measure such as GCUPS (Giga Cell Updates per second) that is commonly used to compare the performance of dynamic programming algorithms on various platforms.

We appreciate your suggestions for comparison to other state-of-the-art DTW and sDTW implementations. Even though GCUPS or TCUPS is a commonly used performance metric to compare the performance of generic DTW implementations, our work is highly application-specific and focuses on optimizing the selective nanopore sequencing problem for resource-constrained devices. Our work is not meant to be a generic sDTW processor but rather an overarching system architecture composed of an application-specific sDTW processor for selective sequencing purposes. Therefore, the real metric that is relevant to our work is the number of genomic reads processed per second, which we have included in our experimental results. In the updated manuscript we have included comparisons with state-of-the-art solutions exclusively focused on selective sequencing (see the section “Comparison with alternate methods” on page 6 of the manuscript and Supplementary Note 4) We have also added the above papers to the related work section on page 10 of the updated manuscript.
