# Supplementary material for: Efficient real-time selective genome sequencing on resource-constrained devices
Source: Gigascience. 2023 Jul 3;12:giad046. doi: 10.1093/gigascience/giad046 (PMC10316692; doi:10.1093/gigascience/giad046)
Supplement: giad046_Supplemental_File [file giad046_supplemental_file.pdf]

Supplementary Information: Efficient Real-Time Selective Genome Sequencing on Resource-Constrained Devices

Po Jui Shih[1], Hassaan Saadat[2], Sri Parameswaran[3] and Hasindu Gamaarachchi[1,4]

[1]School of Computer Science and Engineering, UNSW Sydney, Australia
[2]School of Electrical Engineering and Telecommunications, UNSW Sydney, NSW, Australia
[3]School of Electrical and Information Engineering, University of Sydney, Australia
[4]Kinghorn Centre for Clinical Genomics, Garvan Institute of Medical Research, Sydney, Australia

Supplementary Note 1: Effect of fixed-point scaling on accuracy....1
Supplementary Note 2: Resource utilisation of multiple accelerators....2
Supplementary Note 3: Exploring the sDTW score cut-off and its impact on accuracy....3
Supplementary Note 4: Comparison with other works....4
HARU vs DeepSelectNet....4
HARU vs Guppy + Minimap2....5
HARU vs UNCALLED....5
Comparison with SquiggleFilter....6
Supplementary Note 5: Selection of query length....7
Supplementary Note 6: Impact of Individual Optimisations....8
Fixed-point Optimisation....8
Cost matrix memory optimisation....9
Pipelining....9
Supplementary Note 7: Commands used for evaluation....10
Supplementary Note 8: Post-implementation power-analysis of Kria with HARU....12

Supplementary Note 1: Effect of fixed-point scaling on accuracy

The fixed-point scaling method in HARU involves converting the 13-bit integer MinION data and 11-bit integer PromethION data into real numbers through picoampere conversion and then z-score normalisation. These real numbers are then represented and processed in fixed-point representation with a scaling factor of 32 (5 fractional bits) in the HARU hardware to achieve resource efficiency.

The data typically follow a Gaussian distribution which means after normalisation, only around 0.0027% of samples can be larger than 3 in magnitude. We accumulate such values over 500 in the worst case (250 cells along the x-axis and 250 cells along the y-axis for the cost matrix) which means there is a minuscule probability for the magnitude to go over 1024. Hence 16 bit fixed-point types, with 11 integer bits, and 5 fractional bits, are enough to represent raw signals generated by an ONT sequencer with adequate precision for sDTW to map accurately.

We also performed experiments to empirically validate this method for multiple target genomes, including SARS-CoV-2, Ebola, Ecoli, and Lambda datasets publicly available. In these experiments, we measured the accuracy of sigfish using 32-bit float and sigfish-haru using 16-bit fixed-point data type. The accuracy of each version of sigfish measured against the results obtained from Minimap2 (if the mapping coordinate is within 200 bases proximity they are considered correct) only differed by 0-0.05% for all the genomes tested.

In summary, in the case of our sDTW algorithm, the input data comes from a well-defined and consistent range, as explained earlier, making a static scaling factor a more appropriate and efficient choice for hardware implementation. Additionally, fixed-point arithmetic with a set scaling factor can often be faster and more power-efficient than floating-point arithmetic, which requires more complex hardware and consumes more power (see Supplementary Note 6 for the impact of using 32-bit float, 32-bit fixed point, and 16-bit fixed point for HARU).

Supplementary Note 2: Resource utilisation of multiple accelerators

The results shown in this work use a single sDTW accelerator with the sigfish software. However, for devices that have enough FPGA resources multiple accelerators can be deployed in parallel to provide higher throughput. While we have not provided a full system that holds multiple sDTW accelerators we have provided an experimental branch at https://github.com/beebdev/HARU/tree/dynamic-reference which streams references on the fly during real-time execution without using on-chip memory (BRAM) to store reference signals. As the accelerator only exposes a single AXI-Stream interface for the data path and a single AXI-Lite interface for the control path, each accelerator will be independent of the other (no cross-accelerator critical paths). The accelerators interfacing with the ARM processor (PS) on the Zynq device will be using a multi-channel AXI DMA. For the tested target device in this work, the Xilinx Kria AI Starter Kit, the programmable logic has enough resources for 4 accelerators. The post-implementation resource utilisation is provided in Supplementary Table 1 (HARU with single accelerator) and Supplementary Table 2 (HARU with four accelerators). All timing constraints are satisfied.

Site Type	Used	Fixed	Prohibited	Available	Util%
CLB LUTs	26726	0	0	117120	22.82
LUT as Logic	25718	0	0	117120	21.96
LUT as Memory	1008	0	0	57600	1.75
LUT as Distributed RAM	830	0			
LUT as Shift Register	178	0			
CLB Registers	25686	0	0	234240	10.97
Register as Flip Flop	25686	0	0	234240	10.97
Register as Latch	0	0	0	234240	0.00
CARRY8	1783	0	0	14640	12.18
F7 Muxes	4	0	0	58560	<0.01
F8 Muxes	0	0	0	29280	0.00
F9 Muxes	0	0	0	14640	0.00

Supplementary Table 1. Post-implementation utilisation for HARU system with single

dynamic reference streaming accelerator

Site Type	Used	Fixed	Prohibited	Available	Util%
CLB LUTs	92842	0	0	117120	79.27
LUT as Logic	91385	0	0	117120	78.03
LUT as Memory	1457	0	0	57600	2.53
LUT as Distributed RAM	1256	0			
LUT as Shift Register	201	0			
CLB Registers	79136	0	0	234240	33.78
Register as Flip Flop	79136	0	0	234240	33.78
Register as Latch	0	0	0	234240	0.00
CARRY8	7082	0	0	14640	48.37
F7 Muxes	4	0	0	58560	<0.01
F8 Muxes	0	0	0	29280	0.00
F9 Muxes	0	0	0	14640	0.00

Supplementary Table 2. Post-implementation utilisation for HARU system with four dynamic reference streaming accelerators

Supplementary Note 3: Exploring the sDTW score cut-off and its impact on accuracy

This experiment explores how the sDTW score cut-off affects mapping accuracy. We mix reads of two targets, SARS-CoV-2 and yeast, together and use sigfish-haru to determine whether a read belongs to the SARS-CoV-2 target by mapping to the SARS-CoV-2 reference. If the sDTW score is lesser than a cut-off threshold, the mapping is deemed to belong to SARS-CoV-2, otherwise to yeast. Using a cut-off score applied to the mapping results, the accuracy is affected as seen in Supplementary Figure 1.1 The accuracy is calculated as $\frac{true\,positive + true\,negative}{total\,reads}$. We observe that for the shown results, the accuracy peaks when a cut-off score of 70 is used. Mapping out the DTW score distribution of mappings as seen in Supplementary Figure 1.2, we see that there are two clear bell shapes each representing the positive results (SARS-CoV-2) and negative results (read belonging to the yeast target) with 70 being the overlap.

<table>
<tr><td>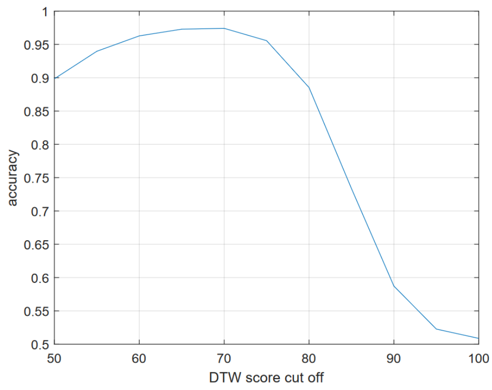
</td><td>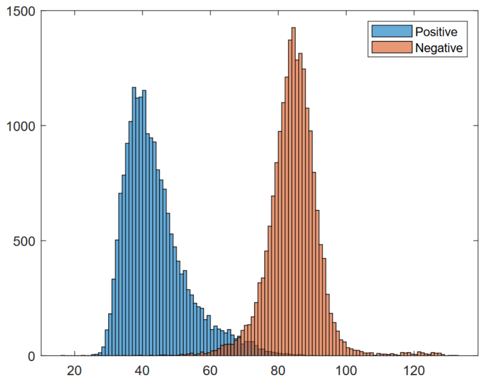
</td></tr>
<tr><td>Supplementary Figure 1.1: DTW score cut-off threshold vs accuracy</td><td>Supplementary Figure 1.2: DTW score distribution of Positive and Negative mappings</td></tr>
</table>

Supplementary Note 4: Comparison with other works

In this note we compare HARU against different selective sequencing methods, providing the throughput and accuracy. The throughput is measured in reads per second while the mapping accuracy is measured as:

$$\frac{true\,positive + true\,negative}{total\,reads}$$

HARU vs DeepSelectNet

DeepSelectNet is a neural-network-based signal-domain selective sequencing work for Nanopore sequencers based on SquiggleNet and provides improvements in both accuracy and throughput compared to. The results for DeepSelectNet were run on a Tesla V100 GPU mapping the SARS-CoV-2 dataset.

Supplementary Table 3. Comparison between HARU and DeepSelectNet

**Method**	**Throughput (reads/s)**	**Accuracy (%)**
HARU	1066.33	97.41
DeepSelectNet	507.099	91.78

Supplementary Table 4. DeepSelectNet mapping results of SARS-CoV-2 and Yeast

	True	False
Positive	18619	1381
Negative	18091	1909

From Supplementary Table 3, we see that HARU exceeds DeepSelectNet's throughput by almost double and the accuracy is slightly higher than DeepSelectNet. Supplementary Table 4 shows the mapping results count for DeepSelectNet. HARU's advantage over

Deepselectnet is the amount of preparation needed is much less as deepselectnet requires the model to be trained before performing selection in real-time.

HARU vs Guppy + Minimap2

We also evaluated the approach used in Readfish, which employs Guppy with fast basecalling followed by Minimap2 for mapping. Since both Guppy and Minimap2 are supported on ARM platforms, we ran Guppy_fast+Minimap2 on an Nvidia Jetson Xavier edge GPU platform. We use 4500 samples of each read to basecall and use the resulting sequence to stream into Minimap2. The versions used were Guppy 6.1.3 and Minimap2 2.22.

Supplementary Table 5. Comparison between HARU and Guppy_fast+Minimap2

**Method**	**Throughput (reads/s)**	**Accuracy (%)**
HARU	1066.33	97.41
Guppy_fast + *Minimap2*	317.94	91.46

Supplementary Table 6. Guppy_fast+Minimap2 mapping results of SARS-CoV-2 and Yeast

	True	False (unmapped)
Positive	19952	48
Negative	16631	3369

We observe in Supplementary Table 5 that HARU is much higher in terms of throughput and provides slightly higher accuracy for the mapping. Supplementary Table 6 shows the mapping results of Guppy_fast+Minimap2.

HARU vs UNCALLED

UNCALLED is considered the state-of-the-art seeding and mapping implementation of selective sequencing in the signal domain. To compare the performance between HARU and UNCALLED, we ran UNCALLED on a Rock64 which has a quad-core ARM Cortex A53 processor. We run UNCALLED with two configurations of chunktime, 0.4 being roughly the same query size (4000*0.4 = 1600samples) as HARU uses and 0.8 (4000*0.8 = 3200 samples) to provide higher accuracy.

Supplementary Table 7. Comparison between HARU and UNCALLED

**Method**	**Throughput (reads/s)**	**Accuracy (%)**
HARU	1066.33	97.41
UNCALLED (Rock64) - 0.4 chunktime	36.78	47.82
UNCALLED (Rock64) - 0.8 chunktime	28.94	91.2

Comparing HARU and UNCALLED on the embedded Rock64 device (Supplementary Table 7) we see that the performance throughput of HARU is 28.99x more than UNCALLED on Rock64 with 0.4 chunk time, and 36.85x more than UNCALLED on Rock64 with 0.8 chunk time. Referring to the accuracy performance we see that UNCALLED with 0.4 chunk time (when translates into 1600 samples for query size) obtains a 47.82% accuracy, while 0.8 chunk time (translating to 3200 samples for query size) obtains a 91.2% accuracy, both being lower than HARU's 97.41% accuracy.

Comparison with SquiggleFilter

It is not possible to practically execute SquiggleFilter on an FPGA with the provided source code of SquiggleFilter due to the absence of a synthesiable top-level module that orchestrates all submodules of the system. The provided performance results in the SquiggleFilter remain theoretical performance metrics and do not consider the overheads that are involved in having a fully integrated system. Instead, we provide insight for comparing hardware resource utilisation between HARU and SquiggleFilter.

Using the provided SquiggleFilter HDL source code and Xilinx Vivado project (https://github.com/TimD1/SquiggleFilter), we set up the system to target the same tested device HARU is using (Xilinx's Kria AI Starter Kit), change the PE count used in SquiggleFilter to 2000 (which is the number needed as stated in the SquiggleFilter work; PE count was set to 2 for synthesis by default), and manually synthesised the submodules of SquiggleFilter (as described by the author in the GitHub issue https://github.com/TimD1/SquiggleFilter/issues/1). Note that we have upgraded the rar compressed Vivado project provided by the SquiggleFilter authors to Vivado 2022.2, matching with the version used to synthesise and implement HARU).

Supplementary Table 8. Resource utilisation for SquiggleFilter's submodules

**Module**	**CLB LUTs**	**CLB Registers**	**CARRY8**
Warper (2000 PEs)	178,553	191,991	22,002
+ PE	88	93	11
Normalizer	284	711	39
Mean finder	275	853	43
Mad finder	Not synthesisable	Not synthesisable	Not synthesisable
**Total (at least)**	**179,112**	**193,555**	**22,084**

Supplementary Table 9. Resource utilisation for Kria with HARU deployed in PL

**Module**	**CLB LUTs**	**CLB Registers**	**CARRY8**
zynq_dtw_0	21,158	16,634	1,760
+ dtw_core (250 PEs)	21,060	16,449	1,760
++ PE	41	16	4

**Total (HARU+others)**	**28,094**	**26,432**	**1,783**

From Supplementary Table 8 and 9, we observe that the PE resource utilisation for HARU is much lower than SquiggleFilter's PE, using 53% less CLB LUTs, 82.8% less CLB Registers, and 63.6% fewer CARRY8s despite SquiggleFilter using 8-bit data types and HARU using 16-bit. Furthermore, since HARU uses events instead of samples of raw signals, as used in SquiggleFilter, the amount of PEs needed is much lower, which results in the overall accelerator having a comparably lower hardware resource utilisation footprint than that of SquiggleFilter (HARU's sDTW accelerator in total uses 88.2% less CLB LUTs, 91.3% less CLB registers, and 92% less CARRY8 than just the warper itself in SquiggleFilter). Note that the total resource shown in Supplementary Table 9 includes all other components in the programmable logic fabric in the system, including the processing system, AXI DMA, and other miscellaneous IP cores. The total resource in SquiggleFilter's utilisation in Supplementary Table 8 excludes the Mad finder (as we are unable to run synthesis for this module), and does not include a top-level module.

Overall, HARU's advantage over SquiggleFilter is its hardware resource efficiency, ease of adapting to change (FPGA vs ASIC design), and provide a full working system from software design to hardware.

Supplementary Note 5: Selection of query length

We performed experiments to determine how the query length and the prefix trim length affect accuracy (Supplementary Figure 2.1). We used the SARS-CoV-2 dataset for this experiment and the accuracy is computed by comparing the output of HARU to that from Minimap2. If the mapping location reported by HARU is within 200 bases from the mapping location reported by Minimap2, such a mapping is considered correct. From Supplementary Figure 2.1 we can see that accuracy increases as the query length increases (a prefix of fixed 50 events are rimmed before the query), which is 88% at query length 250 and saturates at ~99% from 500. This gives the impression that a query length of 500 is much better than 250. However, from Supplementary Figure 2.2, where we use a fixed query length (250) but vary the prefix of the sequence we trim, we see that with a higher trim, we can obtain higher accuracy while keeping the query length constant. For instance, taking 250 events for mapping after trimming 250 initial events, yields an accuracy of >98%. This trimming is to eliminate the prefixes such as the adapter (and barcode if present) that are at the beginning of the read.

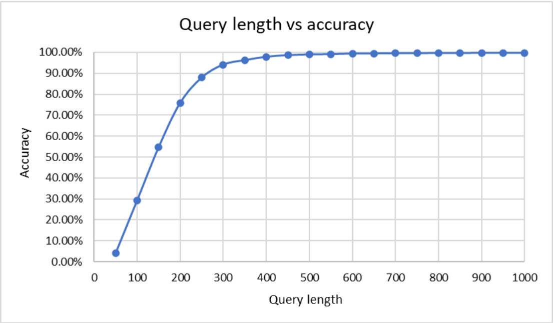

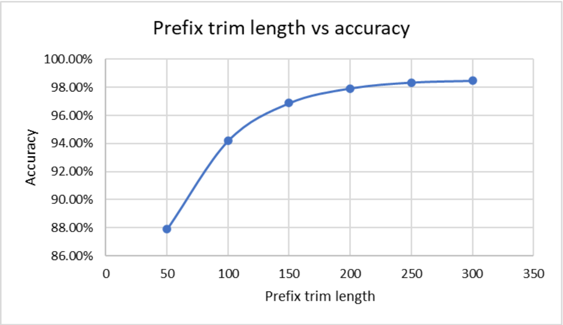

Supp. Fig. 2.1	Supp. Fig. 2.2
Supplementary Figure 2: Impact of query length and prefix trim length on accuracy	

The number of events to be trimmed depends on the sample. For instance, if the sample is not barcoded (SARS-CoV-2 dataset used for Supplementary Figure 2.1), trimming around 150 events is adequate to reach >96% accuracy with a 250 query length. However, around 300 events were required to be trimmed to reach the 96% accuracy for another SARS-CoV-2 dataset which is barcoded (data associated with https://doi.org/10.1093/bioinformatics/btab846). The appropriate trimming length is thus a value that is determinable before the sequencing based on information available during library preparation.

The length of the prefix trimmed is far more important than the query length for accuracy. While higher accuracy can be also achieved instead of mapping a longer query without adequate trimming, this increases the computational space and time, and more importantly in our case, reconfigurable hardware resources. Thus, in HARU, the prefix trimming is performed in software and a fixed 250 event query is sent to the FPGA accelerator for mapping. This 250 query length was selected in our implementation to balance the speed and accuracy. The selection of this (250 query length) value has also been validated for different datasets by Loose et. al. in the RUscripts work (https://www.nature.com/articles/nmeth.3930) which we based our work on. 250 events relate to around 100-150 bases and typically such a large-kmer size would be adequate to locate a genomic coordinate with adequate accuracy for selective sequencing applications. Having said that this query length (`M`) is a variable in the HDL source code that can be changed and synthesised (will consume more area and can be increased up to 1000 events in theory for the Kria FPGA board). However, if we increase it, the accuracy of mapping will increase at cost of adaptive sampling efficiently (number of bases sequenced from unwanted regions; for example rejecting unwanted reads at 250 events at 87% accuracy vs 500 events at 95% accuracy). Also, note that rejecting reads after too many bases have passed through the pore can be detrimental, as long strands passed through the pore when rejected can get clogged and destroy the pore.

Supplementary Note 6: Impact of Individual Optimisations

Fixed-point Optimisation

Using floating points for the sDTW computation can preserve more resolution of the decimals of the samples in the query signal after the z-score normalisation. However, float computations in hardware are generally more expensive in time and resource utilisation. Initially, in our work, we modelled the sDTW accelerator hardware design (PE chains) with Xilinx's Vivado HLS. Using 32-bit floating point data types, due to the timing constraints and the atomic PE's computational time, additional latches were needed in the PE to satisfy timing constraints when clocking at 100MHz. This resulted in the pipelining of PEs having higher initiation interval (II) (14 clocks for the PE pipelining's II, see Supplementary Figure 3). We can see that IIs contribute a lot to the overall task latency of the accelerator as seen in

sub-figure (a) in Supplementary Figure 3. For HARU, the latency of the core mapping operation is computed as:

```
Latency = (M - 1 + N) x II cycles
```

Ideally, II should be one cycle to obtain the least cycles to finish the sDTW mapping. We then modelled our fixed point with the static scaling factor method using 32-bit fixed point and obtain a 2 cycle II (Supplementary Figure 4), and 16-bit fixed point, which gave a 1-cycle II (Supplementary Figure 5). In Supplementary Note 1 we show why 16-bit fixed point is feasible with the static scaling factor. Overall, 16-bit fixed point works for ONT sequencers and provides efficient resource utilisation and computation speed.

Cost matrix memory optimisation

The cost matrix memory optimisation is possible due to backtracking being unnecessary for the selective sequencing application. If pipelining is not applied to the cost matrix computation, the implementation only needs `M+1` cells for storing cost matrix cell values (the additional `1` being used to store the `NW` dependency value). For each cell the score will be calculated as:

```
abs(x[i]-y[j]) + min3(cost[i], cost[i-1], NW)
```

`cost[i-1]` is assigned to `NW` after each cell computation. However, with pipelining, `M` PEs will be computing in parallel and will increase the number of cost matrix cells to `3M-1` (to store all dependency values for each PE, refer to Figure 11 in the manuscript). These cost matrix cells are distributed in the PE design in the HDL code and each PE has the resource utilisation shown below.

Pipelining

Without pipelining, the naive way to compute sDTW in FPGA is similar to how software computes it, which is calculating cell values one at a time. However, the pipelining of cost matrix computation uses a PE chain of size `M`, which computes a column worth of values at a time. As mentioned above, the latency after the pipelining is `(M - 1 + N) x II`. With `M` being a small fixed number in the HARU system (250 by default), the overall performance is linear to the reference size. Each PE's resource utilisation and overall accelerator utilisation are shown in Supplementary Table 10.

Supplementary Table 10. Resource utilisation of HARU's sDTW accelerator (experimental reference streaming)

	CLB LUTs	CLB Registers	Carry8
zynq_dtw_0	21,158	16,634	1,760
+ dtw_core (250 PEs)	21,060	16,449	1,760
++ PE	42	16	4
Total (+ other IP and PS)	28,094	26,432	17,83

a. Overall sDTW hardware model task latency @100MHz

Latency (cycles)		Latency (absolute)		Interval (cycles)		
min	max	min	max	min	max	Type
689928	689928	6.899 ms	6.899 ms	689928	689928	none

b. Cost matrix computation pipelining latency

	Latency (cycles)			Initiation Interval			
Loop Name	min	max	Iteration Latency	achieved	target	Trip Count	Pipelined
- sdtw_col_0_label	7470	7470	30	-	-	249	no
- sdtw_col_j_label	682441	682441	3512	14	1	48496	yes

c. Resource utilization estimates (XC7Z020-CLG484-1)

Name	BRAM_18K	DSP48E	FF	LUT	URAM
DSP	-	-	-	-	-
Expression	-	-	0	18828	-
FIFO	-	-	-	-	-
Instance	0	90	26528	51042	-
Memory	-	-	-	-	-
Multiplexer	-	-	-	12931	-
Register	494	-	65892	927	-
Total	494	90	92420	83728	0
Available	280	220	106400	53200	0
Utilization (%)	176	40	86	157	0

Supplementary Figure 3. sDTW accelerator modelled using 32-bit floating-point

Overall sDTW hardware model task latency @100MHz

Latency (cycles)		Latency (absolute)		Interval (cycles)		
min	max	min	max	min	max	Type
98242	98242	0.982 ms	0.982 ms	98242	98242	none

Cost matrix computation pipelining latency

	Latency (cycles)			Initiation Interval			
Loop Name	min	max	Iteration Latency	achieved	target	Trip Count	Pipelined
- sdtw_col_0_label	747	747	3	-	-	249	no
- sdtw_col_j_label	97490	97490	501	2	1	48496	yes

Resource utilization estimates (XC7Z020-CLG484-1)

Name	BRAM_18K	DSP48E	FF	LUT	URAM
DSP	-	-	-	-	-
Expression	-	-	0	42088	-
FIFO	-	-	-	-	-
Instance	-	-	0	15224	-
Memory	-	-	-	-	-
Multiplexer	-	-	-	10193	-
Register	0	-	36830	621	-
Total	0	0	36830	68126	0
Available	280	220	106400	53200	0
Utilization (%)	0	0	34	128	0

Supplementary Figure 4. sDTW accelerator modelled using 32-bit fixed-point.

Overall sDTW hardware model task latency @100MHz

Latency (cycles)		Latency (absolute)		Interval (cycles)		
min	max	min	max	min	max	Type
49497	49497	0.495 ms	0.495 ms	49497	49497	none

Cost matrix computation pipelining latency

	Latency (cycles)			Initiation Interval			
Loop Name	min	max	Iteration Latency	achieved	target	Trip Count	Pipelined
- sdtw_col_0_label	747	747	3	-	-	249	no
- sdtw_col_j_label	48746	48746	252	1	1	48496	yes

Resource utilization estimates (XC7Z020-CLG484-1)

Name	BRAM_18K	DSP48E	FF	LUT	URAM
DSP	-	-	-	-	-
Expression	-	-	0	26241	-
FIFO	-	-	-	-	-
Instance	-	-	0	17166	-
Memory	-	-	-	-	-
Multiplexer	-	-	-	4625	-
Register	0	-	20808	384	-
Total	0	0	20808	48416	0
Available	280	220	106400	53200	0
Utilization (%)	0	0	19	91	0

Supplementary Figure 5. sDTW accelerator modelled using 16-bit fixed-point.

Supplementary Note 7: Commands used for evaluation

RUscripts on 36-core server:

```
$ cd RUscripts-R9
$ python3 OfflineReadUntil.py -f <reference.fasta> -t
```

```
<target-name>:<region-start>-<region-end> -p 36 -m <k-mer-model> -w <reads-dir> -o
<output-dir> -L 3000 > result.paf
```

Sigfish on 36-core server:

```
$ make
$ ./sigfish dtw -g <reference.fasta> -s <reads.blow5> -t 36 -q 250 -p 50 > server.paf
```

Sigfish on Intel Core-i9 machine:

```
$ make
$ ./sigfish dtw -g <reference.fasta> -s <reads.blow5> -t 8 -q 250 -p 50 > desktop.paf
```

Sigfish on Kria (software only):

```
$ make
$ ./sigfish dtw -g <reference.fasta> -s <reads.blow5> -t 4 -q 250 -p 50 > kria_ps.paf
```

Sigfish-HARU on Kria:

```
$ make fpga=1
$ ./sigfish dtw -g <reference.fasta> -s <reads.blow5> -t 4 -p 50 > kria_pspl.paf
```

Fixed-point with static scaling factor evaluation:

```
$ minimap2 -cx map-ont <reference.fasta> <reads.fastq> --secondary=no > minimap2.paf

$ sigfish dtw -g <reference.fasta> -s <reads.blow5> -p <prefix> > float.paf
$ sigfish-scale dtw -g <reference.fasta -s <reads.blow5> -p <prefix> > 16int_32s.paf

# Evaluation using sigfish
$ sigfish eval  minimap2.paf 16int_32s.paf > int16.acc.txt
$ sigfish eval  minimap2.paf float.paf > float.acc.txt


#sigfish: https://github.com/beebdev/sigfish-haru [commit: 8eb1d0c]
#sigfish-scale: https://github.com/beebdev/sigfish-haru/tree/scaling_profiling [commit:  df8d5ee]
```

Effect of query length on accuracy evaluation script:

```
for i in 50 100 150 200 250 300 350 400 450 500 550 600 650 700 750 800 850 900 950 1000
do
    sigfish-scale dtw nCoV-2019.reference.fasta batch0.blow5 -q $i -p 50 > $i.paf
    sigfish eval batch0.minimap2.paf $i.paf > $i.acc.txt
done
```

Effect of prefix trimming size on accuracy evaluation script:

```
for i in 50 100 150 200 250 300
do
    sigfish-scale dtw nCoV-2019.reference.fasta batch0.blow5 -p $i -q 250 > $i.paf
    sigfish eval batch0.minimap2.paf $i.paf > $i.acc.txt
done
```

SARS-CoV-2 with barcodes read selective sequencing evaluation script:

```
minimap2 -cx map-ont --secondary=no nCoV-2019.reference.fasta
ACW749_pass_355bdcb8_0.fastq > minimap2.paf

sigfish dtw nCoV-2019.reference.fasta ACW749_pass_0.blow5 -p 300 -q 250 > dtw.paf
sigfish eval minimap2.paf dtw.paf > eval.acc
```

Executing DeepSelectNet:

```
# DeepSelectNet - Covid
$ python3 <path-to>/DeepSelectNet/scripts/inference.py -model
<path-to>/COVIDSP1-YEAST/DeepSelectNet/MDL02-mad5/model_92.86_k1/ -s5
<path-to>/COVID/test-covid.blow5 -lb 1 -o <path-to>/COVIDSP1-YEAST/output_covid_k1.txt
-b 1000

# DeepSelectNet - Yeast
$ python3 <path-to>/DeepSelectNet/scripts/inference.py -model
<path-to>/COVIDSP1-YEAST/DeepSelectNet/MDL02-mad5/model_92.86_k1/ -s5
<path-to>/YEAST/test-yeast.blow5 -lb 0 -o <path-to>/COVIDSP1-YEAST/output_yeast_k1.txt
-b 1000
```

Executing Guppy_fast+Minimap2 - on Jetson Xavier:

```
# Guppy basecalling - covid
$ guppy_basecaller -c dna_r9.4.1_450bps_fast.cfg -i COVIDSP1/fast5 -s
COVIDSP1/basecalls/ -x cuda:all

# Guppy basecalling - yeast
$ guppy_basecaller -c dna_r9.4.1_450bps_fast.cfg -i YEAST/fast5 -s YEAST/basecalls/ -x
cuda:all

# Minimap2 Covid
$ minimap2 -x map-ont --secondary=no  mixed-ref.fasta COVIDSP1/reads_covid.fastq >
reads_covid.minimap2.paf

# Minimap2 Yeast
$ minimap2 -x map-ont --secondary=no  mixed-ref.fasta YEAST/reads_yeast.fastq >
reads_yeast.minimap2.paf
```

Executing UNCALLED:

```
# UNCALLED on Rock64 embedded platform running with 0.4 chunk time
$ time uncalled map  nCoV-2019.reference.fasta fast5/ --chunk-time 0.4 --max-chunks 1 -t
32 > uncalled.paf

# UNCALLED on Rock64 embedded platform running with 0.8 chunk time
$ time uncalled map  nCoV-2019.reference.fasta fast5/ --chunk-time 0.8 --max-chunks 1 -t
32 > uncalled.paf

$ uncalled pafstats -r batch0.minimap2.paf uncalled.paf
```

Supplementary Note 8: Post-implementation power-analysis of Kria with HARU

Supplementary Figure 6 shows the power analysis summary of the targeted device (Kria AI Starter Kit) when using a single accelerator HARU. Total on-chip power is estimated to use 2.941 W with 91% of the power used in the ARM processing system. The overall programmable logic, which hosts HARU's sDTW accelerator, AXI DMA IP, and other miscellaneous IPs, is estimated to use only 0.302W. For the SARS-CoV-2 dataset, HARU is

able to process one single read within 0.9356 ms, which means the power consumption per read spent in the programmable logic of the Kria device for HARU is 0.2825mJ.

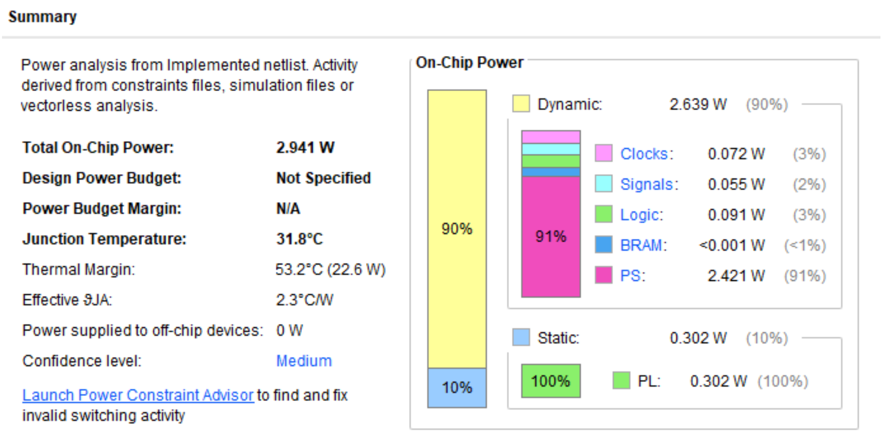

Supplementary Figure 6. Post-implementation power-analysis information of Kria with a single accelerator HARU
