# Supplementary material for: Efficient real-time selective genome sequencing on resource-constrained devices
Source: Gigascience. 2023 Jul 3;12:giad046. doi: 10.1093/gigascience/giad046 (PMC10316692; doi:10.1093/gigascience/giad046)
Supplement: giad046_Response_to_Reviewer_Comments_Original_Submission [file giad046_response_to_reviewer_comments_original_submission.pdf]

Author's Response To Reviewer Comments

Close

The nicely formatted PDF containing the reviewer's response is attached as a supplementary material.

Editor's comments

- Overall, the reviewers are supportive of publication. One major concern mentioned by several reviewers is that the evaluation and comparisons to similar approaches need to be improved, also to highlight differences and improvements compared to existing work.

Initially, we did not provide comparisons with other methods because none of them was designed to be executed on a portable, low-cost and resource-constrained device like the one HARU runs on, which has only 4-ARM cores, 4GB RAM, and consumes less than 3W of power. Nonetheless, to address the reviewers comments in this revised manuscript, we executed those other methods (DeepSelectNet, Minimap2+Guppy, etc) on high-end devices and compared them to HARU executed on a low-end embedded system. Although the difference in execution platform makes the comparison biased in favour of other methods, our results show that HARU still has better performance.

- Reviewer #2 mentions that "the source code for both implementations of the optimized RUscripts in C and Python is not open nor available". Please note it is a requirement for publication in GigaScience that all supporting code is available under an OSI-approved open licence.

The links to these source codes were already provided as open-source under “Availability of source code and requirements” section. Now we have also added the links to all repositories as key-points in the manuscript so those links may not be accidentally overlooked by a reader.

- On a minor note, please also structure your abstract ("Background", "Findings", "Conclusions").

We have structured the abstract as per your suggestion.

Reviewer #1:

This submission is a solid application work to accelerate low-latency, signal-level genome alignment on FPGAs. It provides an end-to-end design for portable sequencers to analyze input genome and discard unnecessary reads in real-time. By providing an open-source RTL design with an integration example, the work brings 2.5× performance with impressive energy efficiency to the community. I would expect this work could facilitate future research.

This paper is well-written, with an extensive explanation of the background. I found it entertaining to read, even with little background knowledge of signal-level processing of genomes. The research goal of low-cost targets and low-latency processing are well motivated. The experimental results are solid and extensive, with an optimized baseline for comparison and end-to-end performance analysis, including overhead time. The authors combine knowledge from different fields and co-optimize the design, for example, the data scaling with acceptable accuracy reduction.

We thank the reviewer for these encouraging comments.

Q1. Although well-written, my main concern about this work is that it seems incremental and needs to exhibit more research innovation. The authors successfully combine multiple optimizations, but most

optimization approaches are well-known. For example, the 2D dynamic programming (DP) algorithm without backtrace requirement is well-studied for not only FPGA accelerations but also from the space reduction perspective. Pipelining and data reusing are both standard practices in the FPGA community. I would appreciate it if the authors could highlight the improvement and difference from the regular 2D DP problem and elaborate more on the novelty of their work at the system integration level.

We appreciate the reviewer's feedback and agree that the initial manuscript did not emphasize the novelty of the system-level integration work sufficiently. Our system-level integration of our end-to-end overarching architecture involves several novel components, such as optimized squiggle preparation for normalization using multithreading, the custom driver for high-throughput transferring via the AMBA AXI4-Stream, and a portable and efficient hardware accelerator. We can also use Kria's xmutil utility tool to swap bitstreams without needing to reboot the system, which makes it easy to adapt to changes or customize towards applications if needed. The hardware control interface uses AXI-Lite, while data streaming (query and reference) uses AXI stream, which enables high throughput on low-cost MPSoC platforms.
We have updated the introduction of the manuscript to highlight the novelty of our system-level integration (page 2).

Q1.2. Another concern is that this work needs to discuss its related work sufficiently. Although there are a few paragraphs on the acceleration of signal alignment, only a few base-alignment approaches are referred to. Although different in the format and semantics of the input data, the algorithm is similar, and I believe the authors could reuse many of the existing accelerator designs. Besides, the discussion on Minimap2 on page 2 is not accurate, as there is at least one published FPGA acceleration (Hardware Acceleration of Long Read Pairwise Overlapping in Genome Sequencing: A Race Between FPGA and GPU), along with GPU implementations. This inaccuracy also undermines the motivation of processing at the signal level. I would appreciate it if the authors further discussed the reason for using a signal-based approach.

Thank you for your feedback on related work. Currently, when performing base-level alignment-based selective sequencing, the bottleneck is basecalling and not the alignment (Minimap2). CPU-based Minimap2 is still very fast and takes only 2-5% of the time in a base-level alignment-based selective sequencing workflow. 95-98% of the time is spent on basecalling, despite using GPU (fast basecalling model). Therefore, those accelerations on Minimap2 are less relevant unless the basecalling in significantly accelerated. We have clarified on page 2 of our report that Minimap2 is not the bottleneck and that the basecalling process is the cause of the bottleneck.

We have also discussed recent acceleration on Minimap2 on page 3.

In fact, the observation that alignment in the base-space is very fast compared to basecalling is what makes signal alignment a potentially better method. We have clarified this on page 3.

Q1.3. There are a few minor comments I would appreciate if the authors could address before publication:

(1.3.1) It seems that as M is implemented as the pipeline depth, it is set to a constant. Does M changes as the data changes? Or does the detection accuracy change if M does not change with the data?

We conducted experiments investigating the impact of query length (denoted by M) on mapping accuracy and found that accuracy increased with longer query lengths. However, trimming the prefix sequence (which belongs to the adaptor and barcode, if present) proved more crucial for maintaining high accuracy. This prefix is pre-determinable and is more desired than having a varying query length. A query length of 250 was chosen to balance speed and accuracy. If for other reasons a different query length is needed our hardware can be updated to accommodate varying query lengths, allowing users to dynamically choose the appropriate bitstream using the Kria platform's xmutil tool. Longer query lengths, though, may require increased squiggle collection wait time and risk pore damage from clogging. We have discussed this in Supplementary Note 5: “Selection of query length” associated with the updated manuscript.

1.3.2 Does the data scaling method work for all data? How about using dynamic scaling instead of a set scale?

The data scaling method we used involves converting the 13-bit MinION integer data and 11-bit PromethION integer data to real numbers using picoampere conversion and z-score normalization. These real numbers are then represented and processed in fixed-point representation with a scaling factor of 32 (5 fractional bits) on the HARU hardware to achieve resource efficiency.

Does the data scaling method work for all data?
We found that the normalized data typically follow a Gaussian distribution, and after normalization, only a very small fraction of samples (0.0027%) have a magnitude greater than 3. We accumulated such values over 512 samples, so the probability of the magnitude exceeding 1024 is negligible. Based on this, we determined that using 16 bits (11 integer bits and 5 fractional bits) is sufficient to represent all types of data without significant loss of precision.

We also performed experiments to empirically validate this method for multiple target genomes, including SARS-CoV-2, Ebola, Ecoli, and Lambda datasets publicly available. Supplementary Note 1, associated with the revised mansuscript, now discusses these experiments and the mapping accuracy percentage difference between sigfish using 32-bit floating points and sigfish-HARU which uses 16-bit fixed-point with scaling method. The accuracy of each version of sigfish measured against the results obtained from Minimap2 (if the mapping coordinate is within 200 bases proximity they are considered correct) only differed by 0-0.05% for all the genomes tested.

How about using dynamic scaling instead of a set scale?
Dynamic scaling can be useful when the dynamic range of the input data is not known in advance, as it allows for the scaling factor to be adjusted on-the-fly to accommodate varying input magnitudes. However, in the case of our sDTW algorithm, the input data comes from a well-defined and consistent range, as explained earlier, making a static scaling factor a more appropriate and efficient choice for hardware implementation. Additionally, fixed-point arithmetic with a set scaling factor can often be faster and more power-efficient than using dynamic scaling or floating-point arithmetic, which requires more complex hardware and consumes more power as discussed in Supplementary Note 6.

1.3.3 In Figure 7, "25" should be "256".

We have updated the manuscript.

Nevertheless, this work is publishable, and the audiences will benefit from its results.

We again thank the reviewer for the positive and constructive feedback that allowed us to improve the quality of the manuscript.

Reviewer #2:
The authors observe that existing Read Until approaches that employ subsequence Dynamic Time Warping (sDTW) algorithm are computationally intensive such that a capable workstation with dozens of CPU cores struggles to keep up with the data rate of a mobile phone-sized MinION sequencer.

The authors present Hardware Accelerated Read Until (HARU), a resource-efficient hardware-software co-design-based method that exploits a low-cost and portable heterogeneous Multiprocessor System-on-Chip (MPSoC) platform with on-chip Field-Programmable Gate Arrays (FPGA) to accelerate the sDTW-based Read Until algorithm. Experimental results show that HARU on a Xilinx FPGA embedded with a 4-core ARM processor is around 2.5× faster than a highly optimized multi-threaded software version (around 85× faster than the existing unoptimized multi-threaded software) running on a sophisticated server with 36-core Intel Xeon processor for a SARS-CoV-2 dataset.

I like this work. The paper appears to provide significant research contributions. It targets tackling an important problem in the genome sequencing pipeline. The following points need to be addressed.

We thank the reviewers for these positive comments.

Q2.1 The authors make several optimization techniques that I appreciate and agree with. However, the effect of each of these techniques on the execution time, FPGA resource allocation, and more importantly the accuracy of Read Until decisions are not provided.

We thank the reviewer for the suggestion to include more details about the effect of our optimization techniques on execution time, FPGA resource allocation, and accuracy of Read Until decisions.

Accuracy:
The pipelining optimization does not change how sDTW is computed and thus does not affect the accuracy. For operations to be pipelined, each operation can only start after its dependent data are ready, and all computations are done exactly the same as non-pipelined computations. Thus, accuracy is not affected by pipelining. We have now briefly stated this in the subsection “Operation pipelining” (page 8) of the updated manuscript.
The cost matrix memory optimization is applicable in the selective sequencing application because we only need either the start or end of the mapping of squiggles in the reference, which means backtracking is not needed as long as we keep track of the minimum value in the last row of the cost matrix. The cost matrix memory optimization still returns the same end position of the mapping as is without the optimization. We have briefly stated this in the subsection “Cost matrix memory optimization” (page 8) of the updated manuscript.
However, we also acknowledge that using fixed-point representation may result in some loss of precision, and we already explored this in detail in the subsection “Fixed-point data representation” of the original manuscript. In addition, in the supplementary Note 1 of the updated manuscript, we have included experimental results that the accuracy of the fixed point representation is nearly identical to using floating point (see the response in reviewer#1’s question)
FPGA resource allocation and Execution time

Different optimizations can interact with one another, potentially resulting in varying execution times and resource allocations when different combinations are applied. As such accurately measuring the effect due to individual optimizations poses a significant difficulty. Therefore, we believe that, ultimately, it's the final execution time and the FPGA resource allocated with all optimizations in place that matter most which we had reported and discussed extensively in the original manuscript. Nonetheless, we have further added Supplementary Note 6 to highlight the impact of different optimizations as best as we can.

Q2.2 I would like to understand more about the effect of choosing 250 events to decide on Read Until. Is sampling more than 250 events needed? The length of read is also an important factor as the length of the ONT reads varies from tens to a few million bases.

We have now discussed this in Supplementary Note 5: “Selection of query length” associated with the updated manuscript. The same question was also raised by reviewer 1. The reviewer is requested to kindly refer to the response to Q1.3.1 - query made by reviewer 1 on M being implemented as the pipeline depth.

Q2.3 The source code for both implementations of the optimized RUscripts in C and Python is not open nor available. I would highly encourage the authors to make them available on github.

We apologize for any confusion caused. The links to these source codes were already provided as open-source under “Availability of source code and requirements” in the original manuscript. The Optimised RUScripts implementation in C is in the same repository as sigfish-haru which can be specified as an option to make:

```
# Building sigfish without hardware acceleration
make PROCESSOR=aarch64
# Building sigfish with hardware acceleration
make fpga=1 PROCESSOR=aarch64
```

To avoid such confusion, we added the following as a key point under the manuscript (page 2).
Q2.4 The evaluation of how HARU scales with a different number of threads is not provided. Both the front

software interface for HARU and the RUscripts are multithreaded, but the number of CPU threads that makes RUscripts faster than HARU needs to be examined.

The experiments in our paper were conducted with RUScripts executed with all threads available on the system, which included 36 cores when executing on the HPC. Although RUScripts was originally written in Python and was not optimized for performance or multithreading efficiency, we re-implemented it in C and optimized it for performance and multithreaded efficiency to provide a fair comparison with HARU. The runtime for the optimized multithreaded implementation of RUScripts was also provided in the paper. As stated in the paper, our HARU system on the edge computing board (costing around $300) is ~85.8× faster than the original RUscripts running with 36 cores on a server and still ~2.49× faster than the 36-core server (costing around $30,000). In the following extract from the original manuscript (now page 4 of the updated manuscript), the fact that all threads were used was stated:

Q2.5 I wonder if the authors also evaluate the benefits of using HARU for base-domain Read Until. The basecalling step contributes significantly to the read accuracy and performing operations in the signal space is still challenging. This is true for most of the Read Until applications. Showing the pros and cons through evaluation of discussion for using HARU for signal domain Read Until versus base domain Read Until can be very useful.

We agree with the reviewer's suggestion that evaluating the benefits of using HARU for base-domain Read Until would be useful. We acknowledge that basecalling is a challenging step in nanopore sequencing and that performing operations in the signal space is still a developing field. However, we believe that signal processing holds great potential for nanopore adaptive sampling on-chip. While piggybacking on existing sequence alignment techniques is easier, signal processing advancements in other fields such as image, video, and audio signal processing have led to the development of miniaturized devices with comparable data rates to nanopore sensors. We have now discussed the pros and cons of each approach under the discussion on page 9 and 10 of the updated manuscript.

Q2.6 For Figure 4, the throughput of the target sequencing machine needs to be added to have a clear comparison with the current need for acceleration.

The relationship between the throughput of the target sequencing machine and the throughput required by the accelerator for effective selective sequencing is very complex. The effectiveness of selective sequencing is determined by whether the reject or sequence decision can be made before the nanopore channel in the sequencer finishes sequencing the current squiggle. The average length of squiggles in nanopore sequencing applications may vary across different targets and can be affected by sample read length distribution. There are also other factors involved in the effectiveness of selective sequencing, including the proportion of on-target and off-target reads in the sample, pore-clogging when a rejection signal is passed back when the nanopore channel has sequenced a majority of the squiggle, software complexities and limitations introduced by MinKNOW (0.4s is the minimum wait time before any data is available). Thus, it is unrealistic to provide any sequencer throughput quantization and discuss its relation with selective sequencing processing throughput to determine Read Until effectiveness.

Q2.7 The authors mention that "Our implementation of HARU loads raw signal from BLOW5 file format because the slow5lib library is lightweight". As the default file format provided by ONT devices is FAST5, I wonder if the execution for format conversion is included in the total execution time.

To clarify, by "lightweight," we meant avoiding the use of bulky libraries. The conversion time is not included because direct loading from RAM through MinKNOW API would eliminate such conversion. However, as the MinKNOW is closed-source and only available in limited settings, we opted to use BLOW5 for this proof-of-concept implementation. It's important to note that BLOW5 is also lightweight in terms of runtime, as demonstrated in https://www.nature.com/articles/s41587-021-01147-4. However, the default file format is controlled by the company, which presents a separate issue.

Q2.8 The reference genome needs to be first loaded to FPGA's block RAM. What if the reference genome doesn't fit into the block RAM? What is the reference genome size that is used in practice for Read Until applications?

In our proof-of-concept implementation, we used block RAMs to load the reference genome, but this is not the only approach. A more flexible way to handle reference genomes is to stream them together with the query signal during runtime, allowing for arbitrarily long references. We have implemented this approach in a separate branch of our code (https://github.com/beebdev/HARU/tree/dynamic-reference), and it allows the accelerator to compute the mappings on the fly while the reference is being streamed into the accelerator.
While the reference streaming branch doesn't limit the reference size, in practice, sDTW-based methods still will not scale for giga-base references. However, we plan to integrate methods such as sigmap and UNCALLED to first perform fast mapping using seeds, followed by running sDTW on potential matches to refine the results. The sDTW mapping operation will involve a much smaller mapping reference size than the full target reference. Currently, these index/seeding-based methods have limitations and require optimizations before they can be fully integrated into our system. We have briefly discussed this under the discussion on page 10 of the updated manuscript.
Q2.9 I understand that the use of low-cost MPSoC is to run the software interface and the hardware accelerator on the same portable device. However, is it possible to use high-end FPGA devices with HBM memories? Is it needed to cope with the throughput of more capable sequencing machines such as PromethION?

While our current implementation targets low-cost MPSoCs, scaling up the hardware implementation to parallelize more PE chain accelerators to handle higher throughputs from more capable sequencing machines such as PromethION is certainly a possibility. Our current design of the accelerator only supports an AXI-stream and AXI-Lite interface, which means that platforms with sufficient resources can parallelize multiple accelerators using multi-channel AXI DMAs. However, efficiently streaming data to a large number of parallel accelerators to achieve the necessary throughput will require faster data buses such as PCIe. Although we have not explored the use of HBM memories or higher-end FPGAs at this point, we appreciate your suggestion for future work and have discussed this possibility under the discussion on page 10 of the updated manuscript.
Q2.10 The command lines for evaluating the existing tools are missing.

We thank the reviewer for reminding this which was overlooked by us. We have added this under Supplementary Note 7.

Q2.11 A few typos:
* page 3, on the left down side, range of i and j have typo.
* On Algorithm 1, first for loop has typo i and j

Thanks. We updated the manuscript and the algorithm.

Reviewer #3:
This paper proposes a hardware-assisted subsequence Dynamic Time Warping solver for selective genome sequencing from raw signal traces from hand-held nanopore sequencers. To improve the efficiency of ReadUntil framework, the proposed framework introduces a systolic array DTW accelerator deployed on an FPGA based SoC, which is integrated into and driven from a host and driver software. Avoiding implementing the full cost matrix allows their design to scale. Improved sDTW processing has achieved large speedup and energy efficiency over a server system with Xeon CPUs.

+ The authors have performed a system-wide study with a full-stack proof-of-concept.
+ Compact PE design that allows it to be implemented on a small FPGA or an edge device.

In this paper, the authors propose a hardware software co-design for selective genome sequencing using sDTW deployed on FPGA. This paper is well written and easy to read. Notably, one of the strengths of this paper is that they demonstrated the full stack implementation of the proposed FPGA solution, and the hardware and software stacks are open-sourced. I guess non-trivial implementation and verification efforts have been made, and I think this can be one of the reasons for this paper to be accepted.

We thank the reviewer for this positive feedback.

Q3.1 Incremental work to SquiggleFilter [37]. The architecture itself is a deployment of a well-known systolic array architecture for dynamic programming including DTW and Smith-Waterman.

The main focus of HARU is the end-to-end integrated system architecture and it is not limited to the sDTW core. We agree that our sDTW core is built upon well known optimizations in hardware (so does the SquiggleFilter) and the primary innovation lies in the overarching system architecture as appreciated by reviewer 1 and 2.

The sDTW core in HARU started as an undergraduate honours thesis project in 2020 and the design was complete by mid 2021, during which the SquiggleFilter publication did not exist. The designs happened to be similar despite being developed independently, because well-known optimizations have been used in both designs. However, as our sDTW core itself was not a complete system and we realised that there is limited utility of such a hypothetical design, we pursued to develop an end-to-end system closer to integration with a sequencer. In fact, the overall system integration turned out to be a more challenging problem than designing the sDTW core itself. We re-iterated our system for overall performance and robustness, leading to the architecture we presented in our paper. While our architecture is based on well-known systolic array designs for dynamic programming, its novelty and practical usefulness comes from its application-oriented end-to-end system-level design and integration with the broader bioinformatics ecosystem.

As suggested by reviewer 1, we have elaborated on the system design more deeply in the introduction of the updated manuscript.

Q3.2 The design is only compared with a single type of target genome (SARS-CoV-2), and the sensitivity to the sample property is not explored deeply.

The computational method of using event detection followed by sDTW for alignment was originally shown in the RUScripts paper by Loose et al, which has already demonstrated its effectiveness through a series of experiments. HARU is built on top of this Loose et al's work, as acknowledged in the original manuscript. Thus, we believe that repeating multiple such experiments is redundant.

The only optimization in HARU that can cause a deviation from the original sDTW algorithm used in RUscripts is the scaling optimization, all other optimizations guarantee the same answer as sDTW used in Loose et al's work (see the response to Q2.1). To evaluate the impact of this scaling optimization, we have now performed more experiments using different samples such as Ebola, Ecoli, and yeast in the supplementary note. We found out that the accuracy mapping location reported by HARU differs from the accuracy from the original sDTW by only 0-0.05% (see Supplementary Note 1).
In terms of "the sensitivity to the sample property", please refer to the Supplementary Not), to see how the sDTW scores can be used to discriminate between two samples.

Q3.3 Comparisons with the state-of-the-art are not provided.

We have added comparisons to Supplementary Note 4 and a brief summary to section Results (page 6) and Methods (Page 9) of the revised manuscript. Please also refer to the response for the related Q3.9 for comparisons between HARU and SquiggleFilter, DeepSelectNet, UNCALLED, Guppy + Minimap2.

Q3.4 The motivation for this paper lies in the fact that direct read mapping (or adaptive sampling) using Read Until cannot keep up with the sequencing throughput without a full-fledged GPU. While direct mapping using the raw current signals in fast5 admittedly has heavy computation loads, it is not clear to me why direct mapping from squiggles is a good option in the first place. MinION's fast basecalling can be done with an edge GPU or mobile devices (e.g., Mk1C can do fast realtime basecalling using its embedded

GPU, and Mk1D is announced to work with iPad Pro's M1 processor and neural engines), and aligning basecalled reads looks much efficient as it needs to deal with less data (fastq is a lot smaller (1/5 ~ 1/10 in size), compared to fast5). While they try to "revitalize the direct signal approach," the motivation and the goal of this work, especially why it should start from the raw current signals, do not seem to be clear. Also, the accuracy is only compared with
RUscripts with sDTW but not with a pipeline with basecalling that many MinION practices follow.

We acknowledge that basecalling from squiggles is a popular and effective approach for many MinION applications, and we did not intend to suggest that it should be replaced. Rather, our goal was to investigate the feasibility and potential benefits of direct signal mapping, which has been previously explored and is an ongoing research area. We believe that direct mapping from squiggles can provide a more complete and accurate representation of the underlying signal, which may be particularly useful for applications that require higher sensitivity or resolution than current basecalling methods can achieve. This is more of a philosophical discussion and we have added a section called “Signal-level vs base-level selective sequencing” on page 9 of the discussion of the updated manuscript.

Reading raw-signals has no such heavy computational bottlenecks if efficient formats such as BLOW5 [https://www.nature.com/articles/s41587-021-01147-4] are used, the problem is in FAST5 rather than with the raw signal. If the raw signal is directly loading from RAM through MinKNOW API, that would anyway eliminate such loads. However, as the MinKNOW is closed-source and only available in limited settings, we opted to use BLOW5 for this proof-of-concept implementation.

Mk1C can do some limited fast realtime basecalling, however, the best-case throughput mentioned on the manufacture’s website cannot be not achieved in practice when we tested. When using Mk1C for selective sequencing, however, enrichment observed is very limited. This is why we currently use Mk1B connected to a workstation with a 3090 GPU for our other in-house selective sequencing experiments that perform redfish. While the Mk1D is announced, it doesn’t yet exist and thus we cannot reach any conclusions.

Regarding the comparison with existing methods, we agree that it would be useful to evaluate the performance of our approach against a pipeline that includes basecalling which we have addressed in the response to Q3.9.

Q3.5 Fundamentally, the sDTW-based approach lacks the seeding step which is critical in the general sequence alignment to reduce search space in the reference genome, so every time all possible positions in the reference need to be searched, and the applicability of this approach looks limited.

We agree that the seeding step is a common technique used in the sequence alignment to reduce the search space in the reference genome, and it can be especially helpful when dealing with long-read sequences. As mentioned in the discussion of our original manuscript, other tools such as sigmap and UNCALLED have already implemented a seeding step and indexing and these tools are continually evolving. Our work with HARU is intended to complement these tools by being used as an accurate refinement step after approximate search methods such as chaining is performed. In fact, general sequence alignment tools such as Minimap2 perform a variant of Smith-Waterman called Suzuki's formulation on potential mapping locations found by chaining.

Q3.6 SquiggleFilter [37] has introduced a very similar approach for raw signal (squiggle) filtering, using a wide sDTW systolic array on ASIC (their work has also verified with FPGA), in order to quickly find viral genomes. The main difference between SquiggleFilter and this work lies in the input granularity: SquiggleFilter uses 2000 sample points while this work uses 250 events, where each sample point or event needs one PE. I think the authors' approach of using a small number of systolic PEs makes sense to reduce resource amount, however, similar systolic array approaches using the partial diagonal PE band for dynamic programming algorithms such as sDTW and Smith Waterman have been well-known from decades ago [ref1, ref2], and I personally did not find their hardware design has much new insights or novelty compared to the prior works. I believe integrating them into one system with a host software for sure requires non-trivial engineering work, but as an academic paper I think this work is a bit too incremental.

In addition to our response for Q3.1 about the squiggleFilter and novelty, we also like to remind that this article is submitted as a technical note rather than a research article.

Regarding the SquiggleFilter being verified on FPGA, it seems that the authors of SquiggleFilter, despite stating the need for 2000 PEs for 2000 sample points, used only 2 PEs to synthesise for the FPGA implementation results (and only synthesised on AWS instance) while using 100 PEs for behavioural simulation (functional verification). Furthermore, the source code they provided does not contain a synthesizable top-level module that collectively connects all submodules. We opened a GitHub issue on these topics and have received confirmation that the synthesis verification for FPGA was done individually for each submodule (as they do not have the full system and only envision it) and the number of PEs used for synthesis was indeed done on a smaller number that is not 2000 (see https://github.com/TimD1/SquiggleFilter/issues/1). In addition, SquiggleFilter is an ASIC design that is expensive to manufacture and integrate with a full SoC and is vulnerable to selective sequencing requirement changes, while the effort needed to update HARU is much easier. As mentioned in the manuscript, HARU is a low-cost and highly efficient end-to-end Read Until solution that runs on off-the-shelf low-cost FPGA SoCs, which we believe will highly benefit the ongoing improvements in signal-level alignment research in genomic sequencing. Overall, SquiggleFilter does not provide a fully integrated working system (as re-stated by the author in the issue linked above) which is the main difference between HARU and SquiggleFilter. Please refer to the response for Q3.9 for comparison with SquiggleFilter.

Q3.7 Recent work, SquiggleNet [ref3], also proposes a similar pre-filtering approach of raw squiggles using a machine learning model. This model is very lightweight and shown to have good accuracy. It also needs a GPU for fast filtering, but because it is based on the well-known ResNet model, it can be easily accelerated by an accelerator such as an edge TPU. I wonder how the sDTW-based approach compares with such ML-based ones.

While neural network-based methods have some advantages, they also have their own limitations. One significant issue is that they require training for every new dataset, which can be time-consuming and computationally expensive. Moreover, they may not be suitable for applications that require per-position mapping or depletion/enrichment analysis, as they typically require both positive and negative training data from both samples.
Regarding the use of edge TPUs to accelerate SquiggleNet, please note that typical edge TPUs are designed to support int8 types for energy- and power efficiency (e.g., https://cloud.google.com/edge-tpu), while SquiggleNet reports experimentation and implementation on workstation GPUs and does not mention the use of int8. While it is possible to convert the model to int8, it will require additional research and optimization effort, and it is unclear how it would affect the accuracy and efficiency of the model. Thus, using edge TPUs for NN-based selective sequencing is yet a hypothesis.
As for the comparison with neural network-based methods, see the response for Q3.9 that compares with state-of-the-art methods, where we compare HARU with DeepSelectNet, which is a work recently published that optimizes SquiggleNet. Comparing throughput results, HARU exceeds the throughput of DeepSelectNet and the accuracy is slightly better, despite the fact that DeepSelectNet was ran on a HPC system with a high-end Tesla V100 GPU, whereas the HARU runs on a low-power, resource constrained device.

Q3.8 The evaluation can be improved to have more depth. They only compared a single type of viral genome (SARS-CoV-2) and a narrow region of a human genome. The sensitivity or robustness to the property of input genome, such as repetitions, variants, mutations, and read length, and to the diversity of the mixes of the genome being sequenced (e.g. respiratory metagenome has about 1000:1 human to viral genome ratio) are not explored.

Please see the response to Q3.2 on experiments using different input genomes. Also, see Q3.12 for the effect of mutations. For experiments on the effect of the query length, please refer to the response to Q1.3 - query made by reviewer 1 on M being implemented as the pipeline depth and also Supplementary Note 5.

Q3.9 The comparisons have been made based on the RUscripts (and sDTW), but it might be a bit narrow-

scoped. It would be helpful if authors can include quantitative comparisons with the state-of-the-art work including SquiggleFilter and SquiggleNet, and other approaches not based on RUscripts or sDTW (like Minimap2 or UNCALLED). I would also make this paper insightful if the authors can provide a discussion on signal alignment and base alignment.

They reason why we did not provide comparisons was because none of the existing methods could execute on a resource-constrained device like the one HARU runs on, which has only 4-ARM cores, 4GB RAM, and consumes less than 10W of power (power analysis from post-implementations reports 2.941W for the whole system. See Supplementary Note 8). However, in this revised manuscript, we executed those other methods on high-end devices where possible and compared them to HARU executed on a low-end embedded system. Although the difference in execution platform makes the comparison biased in favour of other methods, our results show that HARU still has better performance.

We have added those comparisons to Supplementary Note 4 and a brief summary to section Results (page 6) and Methods (Page 9) of the manuscript.

Q3.10 The authors claim up to 5 parallel query processors can be supported by the tested board, but given the LUT utilization for a single processor, I doubt if 5 instances can actually be placed & routed.

We thank the reviewer for pointing this out. The doubt regarding whether 5 instances of accelerators can be placed and routed for the target device is valid. To verify the ability to fit multiple accelerators in parallel, we have now included a separate branch (https://github.com/beebdev/HARU/tree/dynamic-reference) that allows for streaming the query and reference signals together, which eliminates the need for storing the reference signal in the FPGA's block RAM. This makes each accelerator independent of each other and does not contain critical paths between the accelerators. Through synthesis and implementation, we see that the most used resource for a single instance of the accelerator is the CLB LUT, taking up to 22%
