# Supplementary material for: Efficient real-time selective genome sequencing on resource-constrained devices
Source: Gigascience. 2023 Jul 3;12:giad046. doi: 10.1093/gigascience/giad046 (PMC10316692; doi:10.1093/gigascience/giad046)
Supplement: giad046_Response_to_Reviewer_Comments_Revision_1 [file giad046_response_to_reviewer_comments_revision_1.pdf]

Author's Response To Reviewer Comments

Close

Editor comments:

- Please address the minor follow-up request by reviewer #3 (see below).
addressed below

- Our data curators will prepare an archival snapshot of your software repositories, and other supporting data, if applicable. They will contact you shortly with any requests they may have.
this has been completed

- Your Zenodo repository with test data is currently under a cc-BY licence. Supporting datasets for GigaScience articles need to be under a public domain waiver (cc0), this can be changed in the Zenodo settings.
changed to cc0

- At this stage, you can remove any highlighting in colour / tracking of changes that was made for the purpose of peer review.
done

- In addition, please register any new software application in the bio.tools and SciCrunch.org databases to receive RRID (Research Resource Identification Initiative ID) and biotoolsID identifiers, and include these in your manuscript in the "availability" section.
this has been done for the primary tool in the manuscript, HARU.

Reviewer comments:

Reviewer #2: The authors have addressed all my comments.

Reviewer #3: I would like to thank the authors for their detailed responses and clarifications to my comments and questions. There still appear to be some fundamental limitations to the authors' approach; however, as a manuscript submitted as a technical note, I appreciate the effort they have put into implementing and open-sourcing the hw/sw co-designed system, which I believe has the potential to generate new research ideas for our community. The revision that compares the proposed design with other alternatives should also help readers understand the merits.

One follow-up comment to the revised manuscript:
It would be beneficial if the authors can clarify how they obtained the accuracy numbers in Figures 7 and 9. Since using events is shown to reduce the accuracy of mapping, I am not sure there will be a 99% similarity in output (fig.7) for a long reference.

Reviewer #3: It would be beneficial if the authors can clarify how they obtained the accuracy numbers in Figures 7 and 9. Since using events is shown to reduce the accuracy of mapping, I am not sure there will be a 99% similarity in output (fig.7) for a long reference..

Figure 7 shows the accuracy of using the 16-bit fixed-scaled method in sDTW compared to using 32-bit floating point in sDTW calculations. The high similarity rate indicates minimal mapping loss compared to 32-bit floating point values and not accuracy of alignment. This is clarified in “Accuracy” subsection under Section “Results” in page 5 of the manuscript as:
“Figure 7 shows the accuracy of the accelerator using different scaling factors (discussed in section "Software Processing Layer"). Accuracy in Figure 7 is calculated as a percentage of the number of mapping

positions similar to results produced from sDTW computed on software using 32-bit floating points.”

As for Figure 9, the accuracy is the sum of true positive and true negative mapping result counts divided by the total amount of reads. The accuracy shown in Figure 9 is for the SARS-CoV-2 dataset and not for arbitrary length of references. These information are stated in Supplementary Note 4 which is referred to in the main manuscript where the accuracy is discussed:

“The throughput is measured in reads per second while the mapping accuracy is measured as:
(true positive + true negative)/(total reads)
"

Close
