# Supplementary material for: Efficient real-time selective genome sequencing on resource-constrained devices
Source: Gigascience. 2023 Jul 3;12:giad046. doi: 10.1093/gigascience/giad046 (PMC10316692; doi:10.1093/gigascience/giad046)
Supplement: giad046_Response_to_Reviewer_Comments_Revision_2 [file giad046_response_to_reviewer_comments_revision_2.pdf]

Author's Response To Reviewer Comments

Close

1) Please include a citation to your new GigaDB dataset (including the DOI link) to your reference list, and cite this in the data availability section.

The citation is:
[xx] Shih PJ, Saadat H, Parameswaran S, Gamaarachchi H. Supporting data for "Efficient Real-Time Selective Genome Sequencing on Resource-Constrained Devices" GigaScience Database. 2023. http://dx.doi.org/10.5524/102396

In the data availability section, please write something along the lines, "An archival copy of the code and supporting data is also available via the GigaScience repository, GigaDB [xx]".

done

2) Please also include the Zenodo repo in the bibliography in the same way as the gigaDb example above, and also cite the Zenodo repo by number from the availability section.

The citation for the Zenodo is:

[xx] Po Jui Shih, Hassaan Saadat, Sri Parameswaran, Hasindu Gamaarachchi. Efficient Real-Time Selective Genome Sequencing on Resource-Constrained Devices [Data set]. Zenodo 2022. https://doi.org/10.5281/zenodo.7314838

done

3) The artic network URL in the avail. section (https://community.artic.network/t/links-to-raw-fast5-fastq-data-for-artic-protocol/17) should also be moved to the bibliography and cited by number, analogous to the examples above.

done

4) For the NCBI accession number SRX11368475, please just mention the accession number in the avail. section (no URL).

done

5) Please add ORCIDs to the title page (we have the following on our files: ORCID iDs: Po Jui Shih [0000-0001-9088-4409]; Hassaan Saadat [0000-0003-3691-4130]; Sri Parameswaran [0000-0003-0435-9080]; Hasindu Gamaarachchi [0000-0002-9034-9905])

done

6) Please note that the article style does not allow footnotes - please incorporate them in the text, where applicable.

done

7) We only have a PDF at the moment, please submit editable source files to Editorial manager (word or LaTex - if you used LaTex, please also include style and bibliography files).

latex source attached

Close
