# Supplementary material for: Efficient real-time selective genome sequencing on resource-constrained devices
Source: Gigascience. 2023 Jul 3;12:giad046. doi: 10.1093/gigascience/giad046 (PMC10316692; doi:10.1093/gigascience/giad046)
Supplement: giad046_Reviewer_1_Report_Original_Submission — Jason Lau -- 12/22/2022 Reviewed [file giad046_reviewer_1_report_original_submission.pdf]

Reviewer Report

Title: Efficient Real-Time Selective Genome Sequencing on Resource-Constrained Devices

Version: Original Submission **Date:** 12/22/2022

Reviewer name: Jason Lau

Reviewer Comments to Author:

This submission is a solid application work to accelerate low-latency, signal-level genome alignment on FPGAs. It provides an end-to-end design for portable sequencers to analyze input genome and discard unnecessary reads in real-time. By providing an open-source RTL design with an integration example, the work brings 2.5Ã— performance with impressive energy efficiency to the community. I would expect this work could facilitate future research.This paper is well-written, with an extensive explanation of the background. I found it entertaining to read, even with little background knowledge of signal-level processing of genomes. The research goal of low-cost targets and low-latency processing are well motivated. The experimental results are solid and extensive, with an optimized baseline for comparison and end-to-end performance analysis, including overhead time. The authors combine knowledge from different fields and co-optimize the design, for example, the data scaling with acceptable accuracy reduction.Although well-written, my main concern about this work is that it seems incremental and needs to exhibit more research innovation. The authors successfully combine multiple optimizations, but most optimization approaches are well-known. For example, the 2D dynamic programming (DP) algorithm without backtrace requirement is well-studied for not only FPGA accelerations but also from the space reduction perspective. Pipelining and data reusing are both standard practices in the FPGA community. I would appreciate it if the authors could highlight the improvement and difference from the regular 2D DP problem and elaborate more on the novelty of their work at the system integration level.Another concern is that this work needs to discuss its related work sufficiently. Although there are a few paragraphs on the acceleration of signal alignment, only a few base-alignment approaches are referred to. Although different in the format and semantics of the input data, the algorithm is similar, and I believe the authors could reuse many of the existing accelerator designs. Besides, the discussion on Minimap2 on page 2 is not accurate, as there is at least one published FPGA acceleration (Hardware Acceleration of Long Read Pairwise Overlapping in Genome Sequencing: A Race Between FPGA and GPU), along with GPU implementations. This inaccuracy also undermines the motivation of processing at the signal level. I would appreciate it if the authors further discussed the reason for using a signal-based approach.There are a few minor comments I would appreciate if the authors could address before publication: (1) It seems that as M is implemented as the pipeline depth, it is set to a constant. Does M changes as the data changes? Or does the detection accuracy changes if M does not change with the data? (2) Does the data scaling method work for all data? How about using dynamic scaling instead of a set scale? (3) In Figure 7, "25" should be "256".Nevertheless, this work is publishable, and the audiences will benefit from its results.

Level of Interest

Please indicate how interesting you found the manuscript: Choose an item.

Quality of Written English

Please indicate the quality of language in the manuscript: Choose an item.

Declaration of Competing Interests

Please complete a declaration of competing interests, considering the following questions:

- Have you in the past five years received reimbursements, fees, funding, or salary from an organisation that may in any way gain or lose financially from the publication of this manuscript, either now or in the future?
- Do you hold any stocks or shares in an organisation that may in any way gain or lose financially from the publication of this manuscript, either now or in the future?
- Do you hold or are you currently applying for any patents relating to the content of the manuscript?
- Have you received reimbursements, fees, funding, or salary from an organization that holds or has applied for patents relating to the content of the manuscript?
- Do you have any other financial competing interests?
- Do you have any non-financial competing interests in relation to this paper?

If you can answer no to all of the above, write 'I declare that I have no competing interests' below. If your reply is yes to any, please give details below.

I declare that I have no competing interests

I agree to the open peer review policy of the journal. I understand that my name will be included on my report to the authors and, if the manuscript is accepted for publication, my named report including any attachments I upload will be posted on the website along with the authors' responses. I agree for my report to be made available under an Open Access Creative Commons CC-BY license (http://creativecommons.org/licenses/by/4.0/). I understand that any comments which I do not wish to be included in my named report can be included as confidential comments to the editors, which will not be published.

Choose an item.

To further support our reviewers, we have joined with Publons, where you can gain additional credit to further highlight your hard work (see: https://publons.com/journal/530/gigascience). On publication of this paper, your review will be automatically added to Publons, you can then choose whether or not to claim your Publons credit. I understand this statement.

Yes Choose an item.
