# Supplementary material for: Efficient real-time selective genome sequencing on resource-constrained devices
Source: Gigascience. 2023 Jul 3;12:giad046. doi: 10.1093/gigascience/giad046 (PMC10316692; doi:10.1093/gigascience/giad046)
Supplement: giad046_Reviewer_2_Report_Original_Submission — Mohammed Alser -- 12/25/2022 Reviewed [file giad046_reviewer_2_report_original_submission.pdf]

Reviewer Report

Title: Efficient Real-Time Selective Genome Sequencing on Resource-Constrained Devices

Version: Original Submission **Date:** 12/25/2022

Reviewer name: Mohammed Alser

Reviewer Comments to Author:

The authors observe that existing Read Until approaches that employ subsequence Dynamic Time Warping (sDTW) algorithm are computationally intensive such that a capable workstation with dozens of CPU cores struggles to keep up with the data rate of a mobile phone-sized MinION sequencer.The authors present Hardware Accelerated Read Until (HARU), a resource-efficient hardware-software co-design-based method that exploits a low-cost and portable heterogeneous Multiprocessor System-on-Chip (MPSoC) platform with on-chip Field-Programmable Gate Arrays (FPGA) to accelerate the sDTW-based Read Until algorithm. Experimental results show that HARU on a Xilinx FPGA embedded with a 4-core ARM processor is around 2.5Ã— faster than a highly optimized multi-threaded software version (around 85Ã— faster than the existing unoptimized multi-threaded software) running on a sophisticated server with 36-core Intel Xeon processor for a SARS-CoV-2 dataset.General Comments:I like this work. The paper appears to provide significant research contributions. It targets tackling an important problem in the genome sequencing pipeline. The following points need to be addressed.Specific Comments:* The authors make several optimization techniques that I appreciate and agree with. However, the effect of each of these techniques on the execution time, FPGA resource allocation, and more importantly the accuracy of Read Until decisions are not provided. Figure 7 is limited to only the scaling factor of the squiggle values. I would like to understand more about the effect of choosing 250 events to decide on Read Until. Is sampling more than 250 events needed? The length of read is also an important factor as the length of the ONT reads varies from tens to a few million bases.* The source code for both implementations of the optimized RUscripts in C and Python is not open nor available. I would highly encourage the authors to make them available on github.* The evaluation of how HARU scales with a different number of threads is not provided. Both the front software interface for HARU and the RUscripts are multithreaded, but the number of CPU threads that makes RUscripts faster than HARU needs to be examined.* I wonder if the authors also evaluate the benefits of using HARU for base-domain Read Until. The basecalling step contributes significantly to the read accuracy and performing operations in the signal space is still challenging. This is true for most of the Read Until applications. Showing the pros and cons through evaluation of discussion for using HARU for signal domain Read Until versus base domain Read Until can be very useful.* For Figure 4, the throughput of the target sequencing machine needs to be added to have a clear comparison with the current need for acceleration.* The authors mention that "Our implementation of HARU loads raw signal from BLOW5 file format because the slow5lib library is lightweight". As the default file format provided by ONT devices is FAST5, I wonder if the execution for format conversion is included in the total execution time.* The reference genome needs to be first loaded to FPGA's block RAM. What if the reference genome doesn't fit into the block RAM? What is the reference genome size that is used in practice for

Read Until applications?* I understand that the use of low-cost MPSoC is to run the software interface and the hardware accelerator on the same portable device. However, is it possible to use high-end FPGA devices with HBM memories? Is it needed to cope with the throughput of more capable sequencing machines such as PromethION?* The command lines for evaluating the existing tools are missing.* A few typos:* page 3, on the left down side, range of i and j have typo.* On Algorithm 1, first for loop has typo i and j

Level of Interest

Please indicate how interesting you found the manuscript: Choose an item.

Quality of Written English

Please indicate the quality of language in the manuscript: Choose an item.

Declaration of Competing Interests

Please complete a declaration of competing interests, considering the following questions:

- Have you in the past five years received reimbursements, fees, funding, or salary from an organisation that may in any way gain or lose financially from the publication of this manuscript, either now or in the future?
- Do you hold any stocks or shares in an organisation that may in any way gain or lose financially from the publication of this manuscript, either now or in the future?
- Do you hold or are you currently applying for any patents relating to the content of the manuscript?
- Have you received reimbursements, fees, funding, or salary from an organization that holds or has applied for patents relating to the content of the manuscript?
- Do you have any other financial competing interests?
- Do you have any non-financial competing interests in relation to this paper?

If you can answer no to all of the above, write 'I declare that I have no competing interests' below. If your reply is yes to any, please give details below.

I declare that I have no competing interests

I agree to the open peer review policy of the journal. I understand that my name will be included on my report to the authors and, if the manuscript is accepted for publication, my named report including any attachments I upload will be posted on the website along with the authors' responses. I agree for my report to be made available under an Open Access Creative Commons CC-BY license (http://creativecommons.org/licenses/by/4.0/). I understand that any comments which I do not wish to

be included in my named report can be included as confidential comments to the editors, which will not be published.

Choose an item.

To further support our reviewers, we have joined with Publons, where you can gain additional credit to further highlight your hard work (see: https://publons.com/journal/530/gigascience). On publication of this paper, your review will be automatically added to Publons, you can then choose whether or not to claim your Publons credit. I understand this statement.

Yes Choose an item.
