# Supplementary material for: Efficient real-time selective genome sequencing on resource-constrained devices
Source: Gigascience. 2023 Jul 3;12:giad046. doi: 10.1093/gigascience/giad046 (PMC10316692; doi:10.1093/gigascience/giad046)
Supplement: giad046_Reviewer_3_Report_Original_Submission — Daichi Fujiki -- 12/26/2022 Reviewed [file giad046_reviewer_3_report_original_submission.pdf]

Reviewer Report

Title: Efficient Real-Time Selective Genome Sequencing on Resource-Constrained Devices

Version: Original Submission **Date:** 12/26/2022

Reviewer name: Daichi Fujiki

Reviewer Comments to Author:

Summary:This paper proposes a hardware-assisted subsequence Dynamic Time Warping solver for selective genome sequencing from raw signal traces from hand-held nanopore sequencers. To improve the efficiency of ReadUntil framework, the proposed framework introduces a systolic array DTW accelerator deployed on an FPGA based SoC, which is integrated into and driven from a host and driver software. Avoiding implementing the full cost matrix allows their design to scale. Improved sDTW processing has achieved large speedup and energy efficiency over a server system with Xeon CPUs.Strength:+ The authors have performed a system-wide study with a full-stack proof-of-concept.+ Compact PE design that allows it to be implemented on a small FPGA or an edge device.Weakness:- Incremental work to SquiggleFilter [37]. The architecture itself is a deployment of a well-known systolic array architecture for dynamic programming including DTW and Smith-Waterman.- The design is only compared with a single type of target genome (SARS-CoV-2), and the sensitivity to the sample property is not explored deeply.- Comparisons with the state-of-the-art are not provided.Comments:In this paper, the authors propose a hardware software co-design for selective genome sequencing using sDTW deployed on FPGA. This paper is well written and easy to read. Notably, one of the strengths of this paper is that they demonstrated the full stack implementation of the proposed FPGA solution, and the hardware and software stacks are open-sourced. I guess non-trivial implementation and verification efforts have been made, and I think this can be one of the reasons for this paper to be accepted.The motivation for this paper lies in the fact that direct read mapping (or adaptive sampling) using Read Until cannot keep up with the sequencing throughput without a full-fledged GPU. While direct mapping using the raw current signals in fast5 admittedly has heavy computation loads, it is not clear to me why direct mapping from squiggles is a good option in the first place. MinION's fast basecalling can be done with an edge GPU or mobile devices (e.g., Mk1C can do fast realtime basecalling using its embedded GPU, and Mk1D is announced to work with iPad Pro's M1 processor and neural engines), and aligning basecalled reads looks much efficient as it needs to deal with less data (fastq is a lot smaller (1/5 ~ 1/10 in size), compared to fast5). While they try to "revitalize the direct signal approach," the motivation and the goal of this work, especially why it should start from the raw current signals, do not seem to be clear. Also, the accuracy is only compared with RUscripts with sDTW but not with a pipeline with basecalling that many MinION practices follow.Fundamentally, the sDTW based approach lacks the seeding step which is critical in the general sequence alignment to reduce search space in the reference genome, so every time all possible positions in the reference need to be searched, and the applicability of this approach looks limited.SquiggleFilter [37] has introduced a very similar approach for raw signal (squiggle) filtering, using a wide sDTW systolic array on ASIC (their work has also verified with FPGA), in order to quickly find viral genomes. The main difference between SquiggleFilter and this work lies in the

input granularity: SquiggleFilter uses 2000 sample points while this work uses 250 events, where each sample point or event needs one PE. I think the authors' approach of using a small number of systolic PEs makes sense to reduce resource amount, however, similar systolic array approaches using the partial diagonal PE band for dynamic programming algorithms such as sDTW and Smith Waterman have been well-known from decades ago [ref1, ref2], and I personally did not find their hardware design has much new insights or novelty compared to the prior works. I believe integrating them into one system with a host software for sure requires non-trivial engineering work, but as an academic paper I think this work is a bit too incremental.Recent work, SquiggleNet [ref3], also proposes a similar pre-filtering approach of raw squiggles using a machine learning model. This model is very light weight and shown to have good accuracy. It also needs a GPU for fast filtering, but because it is based on the well-known ResNet model, it can be easily accelerated by an accelerator such as an edge TPU. I wonder how the sDTW based approach compares with such ML based ones.The evaluation can be improved to have more depth. They only compared a single type of viral genome (SARS-CoV-2) and a narrow region of a human genome. The sensitivity or robustness to the property of input genome, such as repetitions, variants, mutations, and read length, and to the diversity of the mixes of the genome being sequenced (e.g. respiratory metagenome has about 1000:1 human to viral genome ratio) are not explored.The comparisons have been made based on the RUscripts (and sDTW), but it might be a bit narrow-scoped. It would be helpful if authors can include quantitative comparisons with the state-of-the-art work including SquiggleFilter and SquiggleNet, and other approaches not based on RUscripts or sDTW (like Minimap2 or UNCALLED). I would also make this paper insightful if the authors can provide a discussion on signal alignment and base alignment.Questions and minor comments:- The authors claim up to 5 parallel query processors can be supported by the tested board, but given the LUT utilization for a single processor, I doubt if 5 instances can actually be placed & routed.- What observation has led you to conclude that "250 events are adequate for mapping"?- What would you do if the input reads need more than 250 events (e.g. targeting only a specific variant of a virus) to do the selective read alignment?- Any quantification of the scalability?[ref1] F. Charot, P. Frison and P. Quinton, "Systolic architectures for connected speech recognition," in IEEE Transactions on Acoustics, Speech, and Signal Processing, vol. 34, no. 4, pp. 765-779, August 1986[ref2] Lipton and Lopresti, "A Systolic Array for Rapid String Comparison." January 1985[ref3] Bao, Y., Wadden, J., Erb-Downward, J.R. et al. SquiggleNet: real-time, direct classification of nanopore signals. Genome Biol 22, 298 (2021).

Level of Interest

Please indicate how interesting you found the manuscript: Choose an item.

Quality of Written English

Please indicate the quality of language in the manuscript: Choose an item.

Declaration of Competing Interests

Please complete a declaration of competing interests, considering the following questions:

- Have you in the past five years received reimbursements, fees, funding, or salary from an organisation that may in any way gain or lose financially from the publication of this manuscript, either now or in the future?
- Do you hold any stocks or shares in an organisation that may in any way gain or lose financially from the publication of this manuscript, either now or in the future?
- Do you hold or are you currently applying for any patents relating to the content of the manuscript?
- Have you received reimbursements, fees, funding, or salary from an organization that holds or has applied for patents relating to the content of the manuscript?
- Do you have any other financial competing interests?
- Do you have any non-financial competing interests in relation to this paper?

If you can answer no to all of the above, write 'I declare that I have no competing interests' below. If your reply is yes to any, please give details below.

I declare that I have no competing interests.

I agree to the open peer review policy of the journal. I understand that my name will be included on my report to the authors and, if the manuscript is accepted for publication, my named report including any attachments I upload will be posted on the website along with the authors' responses. I agree for my report to be made available under an Open Access Creative Commons CC-BY license (http://creativecommons.org/licenses/by/4.0/). I understand that any comments which I do not wish to be included in my named report can be included as confidential comments to the editors, which will not be published.

Choose an item.

To further support our reviewers, we have joined with Publons, where you can gain additional credit to further highlight your hard work (see: https://publons.com/journal/530/gigascience). On publication of this paper, your review will be automatically added to Publons, you can then choose whether or not to claim your Publons credit. I understand this statement.

Yes Choose an item.
