# Supplementary material for: Efficient real-time selective genome sequencing on resource-constrained devices
Source: Gigascience. 2023 Jul 3;12:giad046. doi: 10.1093/gigascience/giad046 (PMC10316692; doi:10.1093/gigascience/giad046)
Supplement: giad046_Reviewer_4_Report_Original_Submission — Bertil Schmidt -- 12/31/2022 Reviewed [file giad046_reviewer_4_report_original_submission.pdf]

Reviewer Report

Title: Efficient Real-Time Selective Genome Sequencing on Resource-Constrained Devices

Version: Original Submission **Date:** 12/31/2022

Reviewer name: Bertil Schmidt

Reviewer Comments to Author:

Subsequence Dynamic Time Warping (sDTW) is an important algorithm for processing signals arising from third-generation genomic sequencers. In particular, certain applications with real-time requirements can benefit from efficient computational platforms for this task. The paper adresses this need by presenting a new FPGA-based solution called HARU. It is demonstrated that the approach is energy-efficient and can outperform a self-written C implementation. In addition, HARU is made publicly available which can be beneficial to the bioinformatics community.A weakness of the paper is the limited comparison to the state-of-the-art in the DTW and sDTW area. There are several newer GPU-based and NVM-based approaches that should be considered, such as- Fernandez, et al. "Accelerating Time Series Analysis via Processing using Non-Volatile Memories." arXiv preprint arXiv:2211.04369 (2022).- Schmidt, et al. "cuDTW++: Ultra-Fast Dynamic Time Warping on CUDA-Enabled GPUs." European Conference on Parallel Processing. Springer, Cham, 2020.- Hundt, et al. "Cuda-accelerated alignment of subsequences in streamed time series data." 2014 43rd International Conference on Parallel Processing. IEEE, 2014.In particular, I suggest using a measure such as GCUPS (Giga Cell Updates per second) that is commonly used to compare the performance of dynamic programming algorithms on various platforms.

Level of Interest

Please indicate how interesting you found the manuscript: Choose an item.

Quality of Written English

Please indicate the quality of language in the manuscript: Choose an item.

Declaration of Competing Interests

Please complete a declaration of competing interests, considering the following questions:

- Have you in the past five years received reimbursements, fees, funding, or salary from an organisation that may in any way gain or lose financially from the publication of this manuscript, either now or in the future?
- Do you hold any stocks or shares in an organisation that may in any way gain or lose financially from the publication of this manuscript, either now or in the future?

- Do you hold or are you currently applying for any patents relating to the content of the manuscript?
- Have you received reimbursements, fees, funding, or salary from an organization that holds or has applied for patents relating to the content of the manuscript?
- Do you have any other financial competing interests?
- Do you have any non-financial competing interests in relation to this paper?

If you can answer no to all of the above, write 'I declare that I have no competing interests' below. If your reply is yes to any, please give details below.

I declare that I have no competing interests

I agree to the open peer review policy of the journal. I understand that my name will be included on my report to the authors and, if the manuscript is accepted for publication, my named report including any attachments I upload will be posted on the website along with the authors' responses. I agree for my report to be made available under an Open Access Creative Commons CC-BY license (http://creativecommons.org/licenses/by/4.0/). I understand that any comments which I do not wish to be included in my named report can be included as confidential comments to the editors, which will not be published.

Choose an item.

To further support our reviewers, we have joined with Publons, where you can gain additional credit to further highlight your hard work (see: https://publons.com/journal/530/gigascience). On publication of this paper, your review will be automatically added to Publons, you can then choose whether or not to claim your Publons credit. I understand this statement.

Yes Choose an item.
